# Prediction of PCR amplification from primer and template sequences using recurrent neural network

**DOI:** 10.1038/s41598-021-86357-1

**Published:** 2021-04-05

**Authors:** Kotetsu Kayama, Miyuki Kanno, Naoto Chisaki, Misaki Tanaka, Reika Yao, Kiwamu Hanazono, Gerry Amor Camer, Daiji Endoh

**Affiliations:** 1 Department of Radiation Biology, School of Veterinary Medicine, Rakuno Gakuen University, 582 Midori-machi Bunkyo-dai, Ebetsu, 069-8501 Japan; 2 University of Eastern Philippines, College of Veterinary Medicine, 6400 Catarman, Northern Samar Philippines

**Keywords:** Biological techniques, Biotechnology, Molecular biology, Mathematics and computing

## Abstract

We have developed a novel method to predict the success of PCR amplification for a specific primer set and DNA template based on the relationship between the primer sequence and the template. To perform the prediction using a recurrent neural network, the usual double-stranded formation between the primer and template nucleotide sequences was herein expressed as a five-lettered word. The set of words (pseudo-sentences) was placed to indicate the success or failure of PCR targeted to learn recurrent neural network (RNN). After learning pseudo-sentences, RNN predicted PCR results from pseudo-sentences which were created by primer and template sequences with 70% accuracy. These results suggest that PCR results could be predicted using learned RNN and the trained RNN could be used as a replacement for preliminary PCR experimentation. This is the first report which utilized the application of neural network for primer design and prediction of PCR results.

## Introduction

PCR primers have been traditionally designed by thermodynamic interaction with the desired templates^1,2^. Primers are designed to increase two respectively significant base sequence specificity and reasonable GC content indicators. The high specificity can prevent mispriming in regions other than the target region, and the GC content of a primer is a major factor in determining the annealing temperature (Tm). Maintaining the Tm value optimally affects the amplification efficiency of primers being used^3^. The reference scaffold for primers with high PCR success has been determined based on the result of trials of up to around 1990 thermodynamic calculations^1^. Indices such as discontinuity of the same base are also empirically determined. The Tm value, which explains the specificity of binding to the template and possibly with the primer dimer, among others, are evaluated to determine the appropriate primer pair on each template. With this, some of the proposed software has been designed. The most frequently used primer design software include PrimerSelect^4^, Primer Express (Applied Biosystems Primer Express Software Version 3.0 Getting Started Guide, 2004), Primer Premier (http://www.premierbiosoft.com/primerdesign/index.html), OLIGO 7^5^, Primer3^6^, and OMP^2^. Of these primer design softwares, Primer3 software provides both a primer design on the Web and a local program (Primer3_core) that corresponds to a large amount of primer design, that becomes a standard for PCR primer design. In particular, Primer3 added some thermodynamic findings in 2007 and 2012^2,7^. Its revision in 2012 provided an added knowledge about DNA duplex stability^8^ which incorporated an algorithm for designing primers on the target^9^. This enabled a primer design even in the boundary regions of exons.

Current primer design techniques allow the design of primers that amplify the subject sequence with high probability resultant of combining thermodynamic theory alongside the experience of many researchers. However, it has not been designed to make predictions about a nucleotide sequence that is said to be "not amplifying" a known template. In some previous cases where amplification was performed with an unexpected template in a PCR experiment, knowledge-feedback was unfortunately not documented. Earlier contributions on PCR primer designs have incorporated these into modifications of thermodynamic laws before being compacted to a primer design software^2,5^. To indicate the presence of a particular DNA or RNA sequence, there is always a need to predict that no PCR will occur at sequences that are not of interest or prime importance. In pathogen detection, PCR primers are selected based on several preliminary experiments to confirm that PCR can predictably occur only with a specifically targeted pathogen^10–13^**.** Since false positives pose a major problem in detecting many pathogens including COVID-19, it is important to develop a method for predicting false positives (https://www.biotechniques.com/covid-19/false-negatives-how-accurate-are-pcr-tests-for-covid-19/). Hence, if specificity of a primer pair can be predicted from nucleotide sequences of primers and templates, hindrances including false-positives can be readily corrected resulting to an accelerated research process.

To enable PCR results from the base sequences of primers and templates, it is necessary to comprehensively evaluate various relationships between primers and a template. We focused on machine learning as a technique for predicting a PCR result from various primer-template relationships. Machine learning has been achieving positive remarkable results particularly on data analysis^14,15^. In machine learning, the results of input data can be predicted based on various factors without assuming a unified theory, through optimizing the coefficients of the perceptron network. When a base sequence of a PCR primer and a template is set as a target of machine learning, it is expected that a character string anchored on the base sequence may be suitable for a target of learning. One of the methods for machine learning that has been successful in processing languages used in human research is a recurrent neural network (RNN)^16–19^. As a feature of natural language processing, sentences can be classified based on the frequency and arrangement of words. It has been considered that if the relationship between the primer and the template related to PCR could be replaced with a word, then, it could be a target of natural language processing.

When predicting PCR results, it is necessary to generate learning data for machine learning from the base sequences of the primer pair and the template. The PCR results obtained in the experiment can be used as the correct answer in supervised learning. Factors influencing PCR by primer pairs and templates consist of different relationships and their positions such as dimers, hairpin loops, and partial complementarity. To comprehensively evaluate atypical relationships of these different factors, it was inferred that the Recurrent Neural Network (RNN), which predicts the meaning of sentences from the frequency and arrangement of words, is optimal. With this, we aimed to build a supervised learning method. We generated a pseudo-sentence from a relationship of a primer pair and a template. The PCR results could be learned using RNN^20^ that is a supervised learning method of a natural language. When this learning method is used, it is expected that new supervised learning can be performed even when the results differ due to variable settings of annealing temperature, among others, for the combination of a specific primer pair and a template. In this study, we report the prediction of PCR results by supervised learning.

## Materials and methods

### Assumption

**Figure 1:**
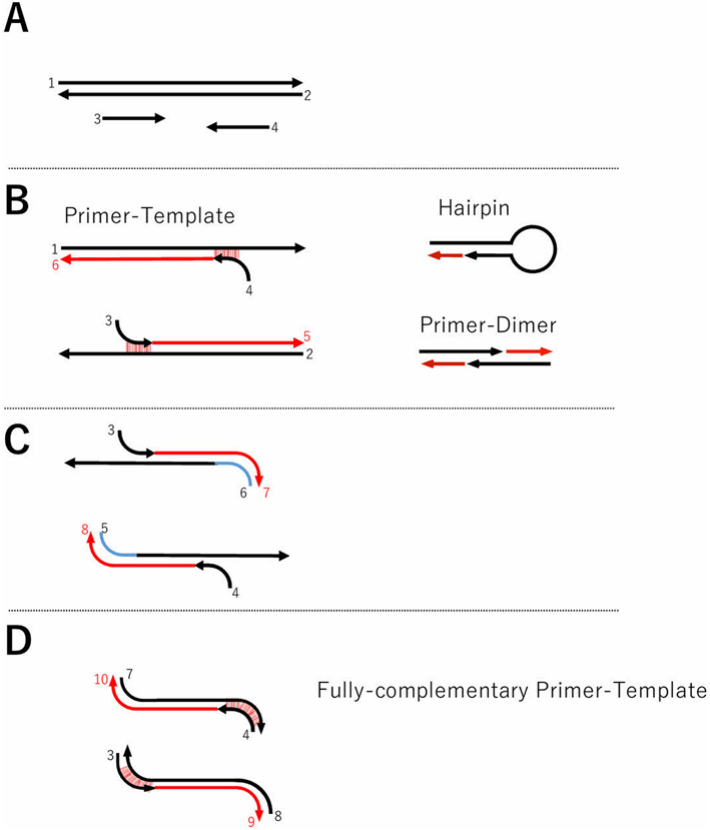
PCR process diagram of primers with incomplete homology with the template. Schematic diagram of the reaction assumed by PCR from partially matched primers. DNA elongation may start from primers on which partially 3′-end matches (**B**). On the end of second cycle, the 3′-end of the elongated DNA completely match with a primer (**C**). On the end of third cycle, synthesized DNA are completely matched on both ends of synthesized DNA (**D**).

To create training data on RNN, the entire PCR reaction was schematically planned. (Fig. 1). Primer binding to the template is not limited to its full length and is assumed that only a part of 3′ may bind (Fig. 1B). Hairpin structure of the primer and its dimer are assumed to be formed before binding the primer to the template (Fig. 1B). Thus, it is assumed that DNA synthesis occurs from some hairpin structures and dimers^21,22^. As DNA synthesis from partially bound primers proceeds, PCR products that are completely complementary to the primers began to be synthesized (Fig. 1C). Eventually, most PCR products become completely complementary to the primers (Fig. 1D).

To express the relationships of these schemas as words, we decided to express the hairpin, primer dimer, primer-template bond, and primer-PCR product bond as words. The strength of the primer-template bond on the forward and reverse flanks greatly influences the establishment of the PCR reaction. For combinations that are not of the original primer-template, the binding position needs to be determined by PCR from the possible binding of multiple primer-templates. With this, we constructed the words for the learning RNN.

### Templates for PCR

A part of the 16S rRNA nucleotide sequence (v6-v8) (Supplement 1 Table 1) was synthesized by OE-PCR (Supplement 1 Table 2) for 30 phyla as templates for PCR model experiments. Of the 30 phyla, 16S rRNA sequences in Firmicutes were synthesized into two genera, the Bacillus and the Calditerricola. These sequences were significantly different in v6-v8. Thirty-one double-stranded DNAs with 435 to 481 bases were prepared as a template for PCR model experimentation utilized the standard thermodynamic index.

### PCR experiment as basic data for primer design using RNN

#### Design of primer sets for preliminary learning of RNN

We designed 72 sets of PCR primers capable of amplifying 31 DNA templates, according to the specifics of the primer sequence to the specific template and the amplification size of about 100–150 (Table 1). In a preliminary trial when primers were designed using Primer3 primer-design software, all primers amplified all 31 templates (data not shown). From its result, we designed 72 sets of PCR primers at this stage ignoring some of the conventionally known annealing temperatures and some indicators such as avoiding single base repetition. The size of the primers was set to 19–22 bases. The most important index is high homology to the target template and low homology to others.

**Table 1:** Primer sets for the main experiment.

Primer no.	Primer name	Sequence
1	aim_1f	GTCCAGGGCTTCACACATGCTA
	aim_22r	TGTTACCAACTTTCATGACGTG
2	aim_71f	AGCGCAACCCTCACCTTATGTT
	aim_94r	GGGACCGGATTTTTGAGATTAG
3	aim_122f	TTCAGTTGGGCACTCGTAAGGA
	aim_94r	GGGACCGGATTTTTGAGATTAG
4	aim_94f	CTAATCTCAAAAATCCGGTCCC
	aim_143r	CCTTCACGAGTTTCACCTTAGT
5	aim_94f	CTAATCTCAAAAATCCGGTCCC
	aim_147r	CTTCACCCCCTTCACGAGTTTC
6	aim_161f	GAGGTGGAGCGAATCCCAGAAA
	aim_194r	CTTACCAAGCATACCTTAGGCA
7	aim_263f	CAAATCCCAGAAAGCCGCTCTC
	aim_250r	ACCAGCCCTGCCGTCGGCGCCT
8	aim_348f	GTGTTGCCTAGCAATAGGATCT
	aim_282r	TGCTGCCCTCTGTCTATGCCAT
9	aim_386f	GCTGAGGACTCTAATTGAACTG
	aim_394r	AGACAGCTTTTAAGGGATTTCC
10	aim_386f	GCTGAGGACTCTAATTGAACTG
	aim_461r	CCGATCCGGACTGAGACAGCTT
11	aim_394f	GGAAATCCCTTAAAAGCTGTCT
	aim_436r	CGAGCGTCTTTGGGTACTCCTG
12	aim_468f	GGCGGAGGAAATCCTAAAAACT
	aim_515r	CTTCAGATACTTCGGGTGCGAC
13	aim_555f	ACGGGACTGCCCGCGAAAGCGG
	aim_562r	GGGCCCACCTTTTTGCGATTAG
14	aim_599f	GTGCTACAACGGGTAGCGAAAC
	aim_652r	CCGCCGAGGCGGAGTTGGGTCA
15	aim_764f	CTTATCCTTTGTTGCCAGCGGT
	aim_744r	CGACATACTTTATGAGGTCCGC
16	aim_812f	CTGCCAGTGATAAACTGGAGGA
	aim_744r	CGACATACTTTATGAGGTCCGC
17	aim_842f	TCTCATAAAACCGTTCTCAGTT
	aim_848r	TGTTACAAACTCTCGTGGTGTG
18	aim_1016f	CTAATCGGAAAAAGCCGGCCTC
	aim_1036r	ATGAATTACACCTTGGGCGGCT
19	aim_1209f	GTGTCGGTAGTTACAGGTGTCT
	aim_1159r	ATTGTCGTGGCCATTGTAGCGT
20	aim_1248f	CGCCGTGACCGGCGGAGGAAGG
	aim_1193r	CCGCGCCATGGCTGATACGCGG
21	aim_1175f	TCGCCTAAACGTGGTCTCAGTG
	aim_1264r	TCCCAGTCGCGGCCCCTGCCCT
22	aim_1177f	GCCTAAACGTGGTCTCAGTGCA
	aim_1264r	TCCCAGTCGCGGCCCCTGCCCT
23	aim_1316f	CTAGTGGGACAGCCGGAGTAAT
	aim_1285r	TGCAATCCGAACTAAGACAAGG
24	aim_1332f	CCGGAGTAATCCGGAGGAAGGT
	aim_1276r	AGGTTTTTGAGGTTGGCTCACT
25	aim_1285f	CCTTGTCTTAGTTCGGATTGCA
	aim_1293r	GCTTCTGGCAAAACCGACTTTC
26	aim_1401f	TGAGGTGTCGGCTTAAGTGCCA
	aim_1368r	GCTAGCTGCCTTCTGTACCCCC
27	aim_1401f	TGAGGTGTCGGCTTAAGTGCCA
	aim_1383r	TTTGGGATTAGCATACGGTCAC
28	aim_1415f	GAGGTGTCGGCTTAAGTGCCAT
	aim_1368r	GCTAGCTGCCTTCTGTACCCCC
29	aim_1447f	AGGTCATGCTGAGGACTCTGGA
	aim_1368r	GCTAGCTGCCTTCTGTACCCCC
30	aim_1447f	AGGTCATGCTGAGGACTCTGGA
	aim_1391r	TTCGATCCGAACTGAGAGAGGA
31	aim_1391f	TCCTCTCTCAGTTCGGATCGAA
	aim_1465r	CCCTAGGACGATCCTTGCGGTT
32	aim_1549f	GGGTAATGCCGGGTACTCACAG
	aim_1504r	CATTGTCCCTGCCACTGTAGCG
33	aim_1504f	CGCTACAGTGGCAGGGACAATG
	aim_1550r	TAGCTCGGGGACTTCCGATGAA
34	aim_1758f	GCCAATACAAACAGTTGCAAAT
	aim_1775r	TACCAGCTCTCATAGTTTGACG
35	aim_1770f	CTGTAAAGTTGGTCTCAGTTCG
	aim_1775r	TACCAGCTCTCATAGTTTGACG
36	aim_1770f	CTGTAAAGTTGGTCTCAGTTCG
	aim_1811r	CTACCCTAGACATGCGCTTCCT
37	aim_1948f	CAAAGGGCAGCGACATAGTGAT
	aim_1984r	ATGAGCCGTAGCTGATGCCCAT
38	aim_2085f	AGTACAGAAGGTAGCAAGATCG
	aim_2138r	AACGTATTCACGGCGTTATGGC
39	aim_2109f	GATGGAGCAAATCCTTAAAGCT
	aim_2164r	TCAACGACTTAAGGTAAAACCA
40	aim_2292f	GGTTAAGTCCCCTAACGAGCGA
	aim_2247r	ATGACTTTGCAGCCTAGCAACG
41	aim_2547f	TCGAGTACATGAAGTTGGAATC
	aim_2581r	TACGGTTAGGCCTGCTACTTCA
42	ai2_1242_f	TACTTTGTCTAACGAGACTGCC
	ai2_1242_r	CGAACTGAGACCAACTTTACAG
43	ai2_100_f	ACGAGCCGGAGGAAGGAGG
	ai2_100_r	ACCCCGGGAACGTATTCACC
44	ai2_1213_f	CCTAAACCCTGTCGTGGTGCAG
	ai2_1213_r	TAGCTCGGGGACTTCCGATGAA
45	ai2_1325_f	TAAGGGGACTGCCCCGGATAAC
	ai2_1325_r	GCGCTTTCTGAGATTCGCTCAG
46	ai2_6_f	CAAGTCGAGCGGAGAAGATTT
	ai2_6_r	GGTATTACCCATCCTTTCGGAT
47	ai2_1194_f	GCGGGTGACCGTATGCTAATCC
	ai2_1194_r	CTTGCGGTTACGTACTTCAGGT
48	ai2_1315_f	CGTTGCTAGGCTGCAAAGTCAT
	ai2_1315_r	GCGGCTCCGGCGACTTCGGATG
49	ai2_1147_f	CGCCGTGACCGGCGGAGGAAGG
	ai2_1147_r	CACTGAGACCACGTTTAGGCGA
50	ai2_23_f	GAGACTGCCGGTGACAAACC
	ai2_23_r	AGTTGCAGACTCCAATCCGGA
51	ai2_1244_f	GCCAATACAAACAGTTGCAAAT
	ai2_1244_r	TACCAGCTCTCATAGTTTGACG
52	ai2_1125_f	ACCGCTGCAACCCCGCGAGGGT
	ai2_1125_r	TGGGCGGCTGCTCCCTTGCGGT
53	ai2_1238_f	GGCACAGGTGGTGCACGGCCGT
	ai2_1238_r	GGCATAAGGGGCACGAGTACCT
54	ai2_1166_f	CCGGAGTAATCCGGAGGAAGGT
	ai2_1166_r	TGCAATCCGAACTAAGACAAGG
55	ai2_1143_f	TGCCGCCGTGACCGGCGGAGGA
	ai2_1143_r	CACTGAGACCACGTTTAGGCGA
56	ai2_1124_f	ACCGCTGCAACCCCGCGAGGGT
	ai2_1124_r	AGCGCACCGACTTCTAGTGCAA
57	ai2_1284_f	ACGAGACTGCCTGGGTTAACCA
	ai2_1284_r	AGCTTTAAGGATTTGCTCCATC
58	ai2_1288_f	CTGCCTGGGTTAACCAGGAGGA
	ai2_1288_r	GAACTGGGGCCAGCTTTAAGGA
59	ai2_1090_f	AAAGGAGACTGCCAGTGATAAA
	ai2_1090_r	TCCAATCCGGACTACGACATAC
60	ai2_1142_f	GTGTCGGTAGTTACAGGTGTCT
	ai2_1142_r	CAACTCCGCCTTCACGGGGGCG
61	ai2_1195_f	GCGGGTGACCGTATGCTAATCC
	ai2_1195_r	CCCTAGGACGATCCTTGCGGTT
62	ai2_101_f	GTCGTCGTCAGCTCGTGCC
	ai2_101_r	CTCCTTCCTCCGCCTCGTC
63	ai2_1189_f	CGTCGTAAGATGTGAGGAAGGT
	ai2_1189_r	TTCGATCCGAACTGAGAGAGGA
64	ai2_1088_f	CTTATCCTTTGTTGCCAGCGGT
	ai2_1088_r	TCCAATCCGGACTACGACATAC
65	ai2_1192_f	GGGGGTACAGAAGGCAGCTAGC
	ai2_1192_r	CTTGCGGTTACGTACTTCAGGT
66	ai2_1303_f	GCCATAACGCCGTGAATACGTT
	ai2_1303_r	CTTCATCCTAGTCATCAGCCTC
67	ai2_1102_f	AAGTTGGGCAGTCTAAGGTGAC
	ai2_1102_r	TCTTGCAGCTCTTTGTACCGTC
68	ai2_54_f	CGGGTGAGTAACACGTATCTAA
	ai2_54_r	TCTCAGTTCGGCTACGTATCAT
69	ai2_1275_f	TGATATGGAGCGAATCCCCAAA
	ai2_1275_r	GTCTGCCTCCTGCAAGCAGGTT
70	ai2_1327_f	CTGAGCGAATCTCAGAAAGCGC
	ai2_1327_r	TTGCCTGGGTTGGGCCACCGGC
71	ai2_10_f	CTGGCGGCGTGGATAAGACA
	ai2_10_r	ATGGGCTATTCCCCACTTCAG
72	ai2_1071_f	AGCGATGCCACCCGGCAACGGG
	ai2_1071_r	CCTGCCCGTAGGCTCCCGGCGA

Primer pair number, primer name and base sequence (5′ → 3′) used in the experiments for RNN-training are shown. Primers with the same primer pair number are used as a set of primers.

We also designed 54 sets of phylum-specific primers, which were designed based on analysis with preliminary test primers (Table 2). As a design method, a plurality of primer candidates was firstly extracted from the template sequence, and a combination of the extracted primer candidates was used as a primer pair candidate. A primer pair for which PCR is expected to occur only in a specific bacterial phylum by RNN was determined as a primer set for a test experiment.

**Table 2:** Primer sets for the test.

Primer set no.	Primer name	Primer sequence
1	1_f_180	AACGCGCTGCGAGCCTGTGA
	1_r_369	CCCACAAGGGTTAGGCCACT
2	1_f_180	AACGCGCTGCGAGCCTGTGA
	1_r_397	ATCGCCGATCCCACCTTCGA
3	1_f_180	AACGCGCTGCGAGCCTGTGA
	1_r_400	CCAATCGCCGATCCCACCTT
4	1_f_180	AACGCGCTGCGAGCCTGTGA
	1_r_408	ACTTCGTCCCAATCGCCGAT
5	1_f_186	CTGCGAGCCTGTGAGGGTGA
	1_r_369	CCCACAAGGGTTAGGCCACT
6	1_f_186	CTGCGAGCCTGTGAGGGTGA
	1_r_397	ATCGCCGATCCCACCTTCGA
7	1_f_186	CTGCGAGCCTGTGAGGGTGA
	1_r_400	CCAATCGCCGATCCCACCTT
8	1_f_186	CTGCGAGCCTGTGAGGGTGA
	1_r_408	ACTTCGTCCCAATCGCCGAT
9	2_f_50	TCAGTTGGGCACTCGTAAGG
	2_r_342	TGGCAAAGACCACTTCGGGT
10	4_f_251	CTAAAGCCACCCCCAGTTCA
	4_r_395	CTCTTCGCCTGACTTCGGGT
11	4_f_251	CTAAAGCCACCCCCAGTTCA
	4_r_403	TCGGCAGGCTCTTCGCCTGA
12	5_f_0	TGCCTGGGAGCCCTAGCACA
	5_r_228	CCCCTTACGGGTTCGCTTCC
13	5_f_223	CAGAGGGAAGCGAACCCGTA
	5_r_410	TCCGGGGGTTGGGATAGCGA
14	6_f_66	GCCTAGCAATAGGATCTCTC
	6_r_211	GGGCATAGTTTAGGGATTGG
15	6_f_211	CCAATCCCTAAACTATGCCC
	6_r_363	AGACGACCTGAGCACTTCTG
16	6_f_211	CCAATCCCTAAACTATGCCC
	6_r_385	TACTAATCACAACTTAGGGC
17	7_f_205	AATCCCTTAAAAGCTGTCTC
	7_r_347	AGCGTCTTTGGGTACTCCTG
18	8_f_78	ACTGCCCAGATCAACTGGGA
	8_r_349	TGGCTTCAGATACTTCGGGT
19	8_f_78	ACTGCCCAGATCAACTGGGA
	8_r_359	TCCTTGCGGTTGGCTTCAGA
20	9_f_87	GACTGCCCGCGAAAGCGGGA
	9_r_181	GTTGCCGGGTGGCATCGCTT
21	10_f_226	TCCCTAAAAAGCATCCTCAG
	10_r_381	AGGCGGAGTTGGGTCACTGA
22	12_f_190	GGCATATACAAAGAGAAGCG
	12_r_395	TAAGCGCCCTCCCGAAGGTT
23	12_f_216	CGAGAGCAAGCGGACCTCAT
	12_r_395	TAAGCGCCCTCCCGAAGGTT
24	12_f_236	AAAGTATGTCGTAGTCCGGA
	12_r_395	TAAGCGCCCTCCCGAAGGTT
25	13_f_203	GGTACAAAGAGCTGCAAGAC
	13_r_397	CTCCAAAAAGGTTACCCCAC
26	14_f_64	GCAAGGGGGCCCTCTGGAGA
	14_r_341	TAGAGCACTCCCTTCTCCCA
27	15_f_60	TGGCGAAACCGCCTCGGATA
	15_r_349	CTCCCTTGCGGTTAGCGCAC
28	18_f_17	TGTCGGTAGTTACAGGTGTC
	18_r_177	GATCTGCACTGAGACCACGT
29	18_f_173	CTAAACGTGGTCTCAGTGCA
	18_r_330	TCCCCGACTGGGGTTAGCAC
30	19_f_83	TAGTGGGACAGCCGGAGTAA
	19_r_177	AGGTCGCATCCCGTTGTCCT
31	19_f_90	ACAGCCGGAGTAATCCGGAG
	19_r_371	CTATCCGAAGATTCGGTCAC
32	19_f_196	TCGCGAGAGTGAGCCAACCT
	19_r_346	TCTGGCAAAACCGACTTTCG
33	19_f_196	TCGCGAGAGTGAGCCAACCT
	19_r_371	CTATCCGAAGATTCGGTCAC
34	20_f_225	ATCCCAAAATCCTCTCTCAG
	20_r_382	GACGATCCTTGCGGTTACGT
35	21_f_61	GGGTAATGCCGGGTACTCAC
	21_r_211	ACCACGACAGGGTTTAGGGG
36	21_f_61	GGGTAATGCCGGGTACTCAC
	21_r_217	ATCTGCACCACGACAGGGTT
37	21_f_61	GGGTAATGCCGGGTACTCAC
	21_r_227	GCAACCCTCAATCTGCACCA
38	21_f_179	AATGGGCTGCAACGCCGTAA
	21_r_359	GTTAGCTCGGGGACTTCCGA
39	21_f_216	AAACCCTGTCGTGGTGCAGA
	21_r_348	GACTTCCGATGAACCCGACT
40	23_f_151	ATGACGTCAGGTACTCGTGC
	23_r_437	CCCCCCTCACCAGGTTCTCC
41	24_f_84	CTGCCAACGTAAGTTGGAGG
	24_r_360	CTTGCGGTTAGCAACACGGT
42	26_f_74	AGACTGCCCGTGTTAAGCGG
	26_r_169	TCACTATGTCGCTGCCCTTT
43	26_f_74	AGACTGCCCGTGTTAAGCGG
	26_r_358	CTGCAAGCAGGTTGGCGCAA
44	28_f_165	AATGGGGCGGACAGAGCGTT
	28_r_333	CCCCCGCTTTGGTGGCTTGA
45	28_f_165	AATGGGGCGGACAGAGCGTT
	28_r_397	ACTTAGTCCCCATCACGGGT
46	28_f_170	GGCGGACAGAGCGTTGCTAG
	28_r_339	GGATGCCCCCCGCTTTGGTG
47	28_f_173	GGACAGAGCGTTGCTAGGCT
	28_r_333	CCCCCGCTTTGGTGGCTTGA
48	28_f_192	TGCAAAGTCATGCTAATCGC
	28_r_333	CCCCCGCTTTGGTGGCTTGA
49	28_f_192	TGCAAAGTCATGCTAATCGC
	28_r_397	ACTTAGTCCCCATCACGGGT
50	28_f_204	CTAATCGCAAAAACCGTTCC
	28_r_397	ACTTAGTCCCCATCACGGGT
51	29_f_237	GCGAATCTCAGAAAGCGCTC
	29_r_437	CCCAGTCGCCAGCCATACCA
52	30_f_128	CAATGCTACGGACAAAGGGC
	30_r_306	TTCGGGCGTGGCCAACTTCC
53	30_f_128	CAATGCTACGGACAAAGGGC
	30_r_328	CCACAAGGGTTGGAGTAACG
54	30_f_128	CAATGCTACGGACAAAGGGC
	30_r_342	TTCGGCGTCCTCCTCCACAA

Primer pair number, primer name and base sequence (5′- > 3′) used in the experiments for RNN-test are shown. Primers with the same primer pair number are used as a set of primers.

#### PCR amplification experiments

Using the 72-primer sets for learning and validation of RNN and 54-primer sets for testing RNN, we tried to amplify all 31 templates. PCR was carried out using 2× GoTaq Green Hot Master Mix (Promega) for a total of 3,906 PCRs with 31 templates and 126 (72 plus 54) sets of primers. The PCR solution contained 0.5 µM primer, 100,000 copies of the template, and was adjusted to 1× GoTaq Green Hot Master Mix by adding water and 2× GoTaq Green Hot Master Mix. After adjustment, the PCR solution was subjected to denaturation at 95 °C for 2 min and followed by 33 cycles at 95 °C for 30 s, 56 °C for 30 s, 72 °C for 30 s, and followed by incubation at 72 °C for 2 min. After cooling to 8 °C, it was stored at 4 °C until processed in agarose gel electrophoresis. The PCR products were electrophoresed using 1.5% agarose in 1× TBE buffer at 100 V for 40 min. The agarose gel was stained in 1 µg/ml Ethidium Bromide solution and photographed under UV.

### Symbols for RNN learning

**Figure 2:**
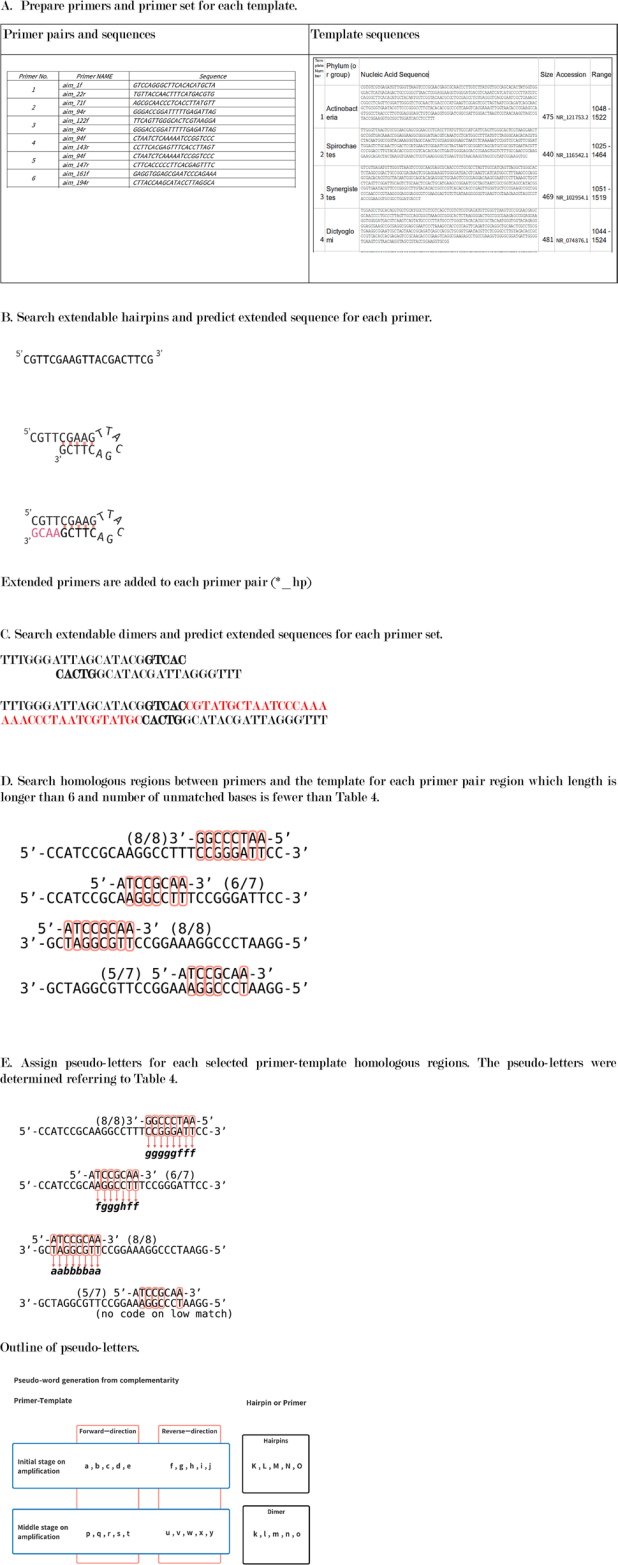

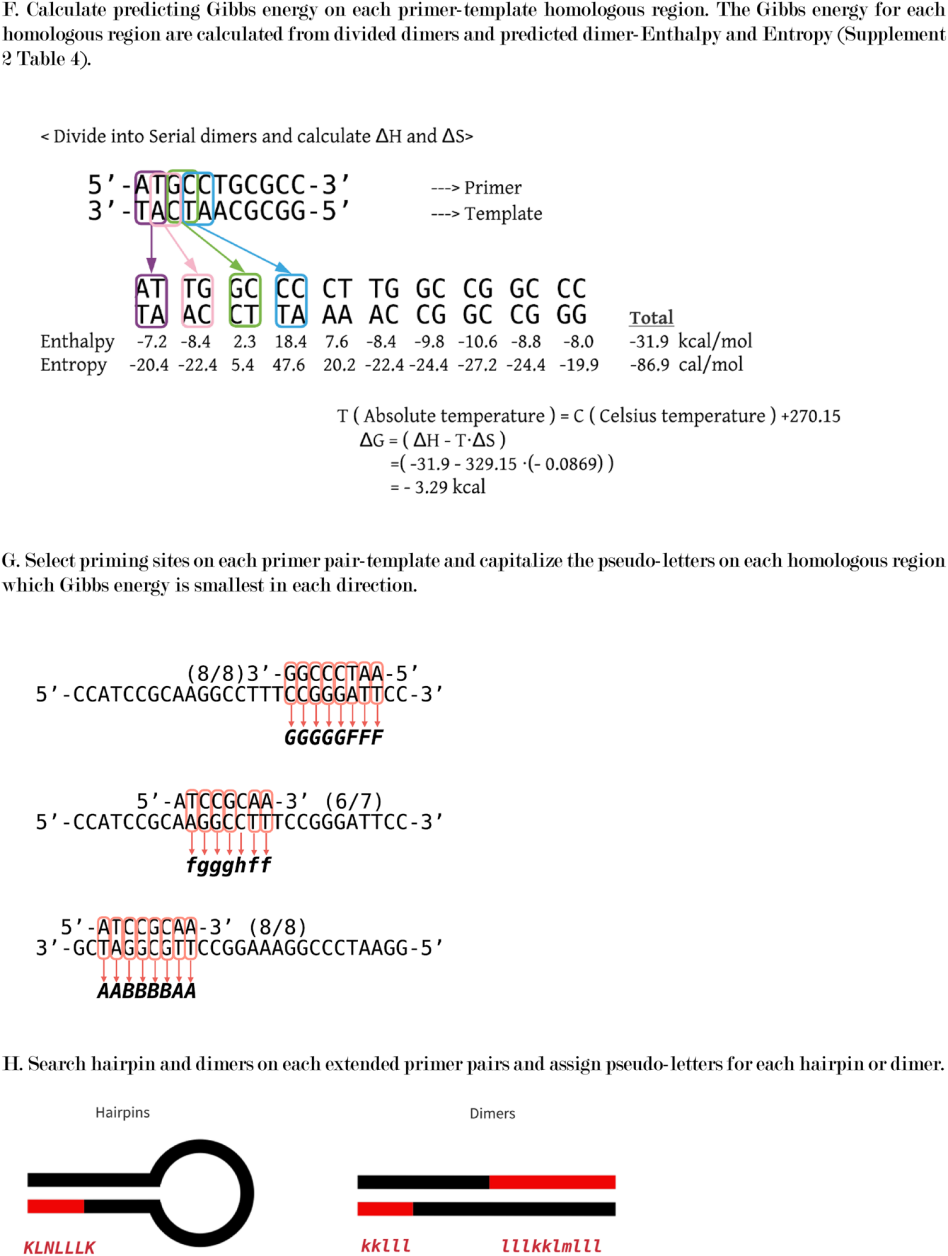

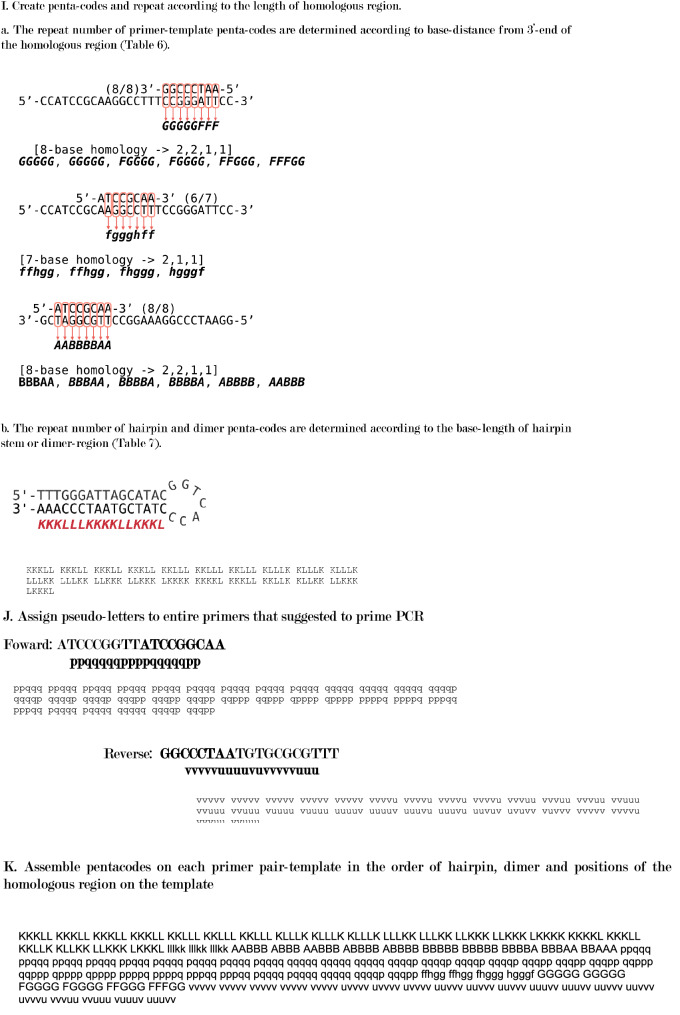
The process of generating pseudo-words and pseudo-sentences is shown. Pseudowords are generated in relation to a particular primer pair and template. First, prepare the primer pair and template data in a format that can be read by the analysis program (**A**). Then, the base sequence alternatives which synthesized on the primer hairpin (**B**) and dimer (**C**) are added to the original primer sequences. The plausible double-strand formation which is expected between the primer sets and template is assumed and expressed as letters (**D**–**E**). First, a part of the complementary primer including a part of the primer and the template and the position of the template are listed (**D**), and their interaction is expressed by a letter for each base-pair (**E**). The one-character code used to express the interaction used at that time is shown in **E**. In order to do machine learning with RNN, it is necessary to predict the primer-binding position on the template, which is the source of the PCR product production. On the prediction other primer-binding positions are classified to unrelated binding positions the PCR product production. In this study, the free energy of each plausible primer binding position on the template was calculated for all possible primer binding positions. Referring to the free energy of binding positions, two primer binding positions, which have minimum free energy, were identified as the PCR-amplifiable primer binding positions. For these determinations, the free energy was calculated on nested dimers and sum free energies on the primer-template binding positions (**F**). The free energies are calculated from Enthalpy, Entry, and absolute temperature of the nested dimers. According to the free energies on the primer-template binding positions, we determined two primer-template binding sites, from which PCR is most likely to proceed, and capitalize nucleotide-interaction-letters (**G**). Similar to primer-template interactions, the program searches hairpin or dimer formation in a primer and primers. One-letter codes are generated for each base pair in these hairpin and dimer (**H**). Strings of interactions between primers or between primers and templates were broken down into 5 letters (five-character codes) as words and duplicated to reflect their importance depending on their length and position from the 3'end (**I**). Similarly, the interaction is predicted for the PCR product and primers shown in Fig. 1D, and characters different from the interaction assumed in the middle of the process are assigned (**J**). A pseudo-sentence is generated by arranging all the five-character codes assigned in this way at positions based on the array of templates (**K**).

The data for RNN learning consisted from a symbol (Table3) generated from the hairpin structure of the primer, the primer dimer, and the homology between the primer and the template, and multiple 5-character codes (pentacode) generated from the symbol (Fig. 2). The correct answer data for RNN was the PCR result for each primer set and template. Since the RNN is optimized for learning natural language sentences, which were composed of words, the generated pentacode is called a pseudo-word, and the pentacode listed according to the nucleotide sequence of the template is called a pseudo-sentence. Specific design methods are described in the creating pseudo-words and pseudo-sentences section.

**Table 3:** Base pair characters for sense or antisense direction.

Base pair (primer base–template base)	Primer-template				Primer hairpin or dimer
	Initial stage		Middle stage		
	Forward	Reverse	Forward	Reverse	
A-T, T-A	a	f	p	u	k
C-G, G-C	b	g	q	v	l
A-A, A-G, G-A, G-G, C-C	c	h	r	w	m
T-T, T-C, C-T	d	i	s	x	n
C-A, A-C, G-T, T-G	e	j	t	y	o

A symbol for generating pseudo-words for RNN learning. The codes are set in the nucleotide duplex on each base pair at the complementary position. Mismatched base pairs such as A-A, T-T and C-A may appear within the partially complementary region. Base-pairs are grouped based on influence for stability of partially complementary strands.

### Creation of pseudo-words and pseudo-sentences from the relationship between primers and templates

For hairpins and dimers, DNA synthesis from the complementary region was predicted and the synthesized primers were added to the primer set. For the complementary region between the hairpin, dimer, primer-template, and primer-PCR product, characters corresponding to the complementary base pair were set for the entire complementary region, and a pseudo-code sequence was generated. The corresponding character string was divided into 5 bases in order from the 3′ end, and 5 bases were repeatedly generated according to the length of the complementary region between the primer-template and the primer-PCR product (pseudo-word). The final pseudo-words were generated in the order of hairpin, dimer, and template forward strand positions.

Hairpin was searched on each primer. Dimers were searched also on possible combinations of primers included in the primer set. The hydrogen bond between primer and template was sought for by any combination of primer-template, primer-primer and 5′-end and 3′-end of a primer.

In probing assumed primer set, the search was performed for both the primer set, and the double-stranded template (Fig. 2A). A complementary region with 5 or more bases was assumed to form a hairpin or dimer, and the relevant region is searched. If present, a 3′-end terminal of the partial duplex was searched. Assuming that complementary strands were synthesized from the partial duplex. When the synthesis of DNA from the partial duplex primers, the additional primers were sequentially incorporated into the primer set (Fig. 2B,C).

As a general rule, the homology between the primer sequence divided into 5 to 22 bases and the template sequence was confirmed, and when the number of bases in Supplement 2 Table 1 was the same (about 80%), a pseudo-code was generated (Fig. 2D). Regarding the homology, area to be generated as a pseudo-code, the pseudo-code was determined by referring to Table 3 for the entire homology, and all lower-case pseudo-codes were generated (Fig. 2E).

Many primer set-template combinations have multiple complementary regions that require priming positions to be determined. Since the complementary region for which such a priming position needs to be determined is short enough, the most stable combination of complementary regions is expected to be the priming position. To determine the most stable complementary region, the combination of complementary regions with the minimum Gibbs energy was set as the priming position (Fig. 2F). The Gibbs energy was calculated according to the formula of DG = DH-TDS by sequentially calculating the entropy and enthalpy of the two bases of the primer and the two bases of the template at the complementary position, assuming an annealing temperature of 56 °C. Therefore, after calculating for all combinations of two complementary bases, the total value was minimized, and the complementary positions of forward and reverse, which are separated by 100 bases or more, were set as the priming positions. Using reference numerical values^23^, complementary dimer set calculations for entropy and enthalpy were done where their original and our extrapolated values were used (Supplement 2 Table 4). The pseudo-code for the complementary position, which was predicted to be the priming position, was converted to uppercase (Fig. 2G). Homologous positions of 6 bases or more were searched for hairpins and dimers, and pseudocodes were generated for the corresponding homologous regions (Fig. 2H). For the pseudo-code sequence generated between the primer and the template, 5 characters were sequentially extracted from the 3′end of the primer to obtain a pentacode. The pseudo-code was generated by repeating a part of the pentacode according to the length of the homologous region to express the strength of the binding between the primer and the template (Fig. 2I).

As for the PCR product, the complementary region of the primer is also completely complementary to the primer because the synthesis proceeds using the primer as a template in the extension reaction (Fig. 1D). For the pseudo-code in this region, a pseudo-code different from the relationship between the template and the primer was set, and a pseudo-code was generated in the same manner as in the complementary region of the primer-template (Fig. 2J). The pentacodes generated from hairpins, dimers were placed first, followed by the primer-templates, and the pentacodes generated from the primer-PCR products in the order of the forward strands of the template. The pentacode was generated and placed from a set of primers and a template was used as pseudo-sentences of the primer set-template (Fig. 2K). Pseudo-sentences were generated for all primer and template combinations and used as learning data during machine learning.

### Scripts for pseudo-sentence generator

A Ruby and Python scripts were used to generate pseudo-sentences in the order shown in Fig. 2 (Supplement 3, List 1–9). The Ruby script read the structure of the template base sequence, primer base sequence, and primer set, and generated pseudo-sentences according to the order shown in Fig. 2. SeqKit (https://bioinf.shenwei.me/seqkit/, v0.14.0) was used to search for homology between the primer and the template. The pseudo-sentences generated for each template-primer set were first categorized by PCR results, and each was categorized into 5 groups. One of the five groups was not used for learning as a group to verify RNN learning but was used to predict the prediction accuracy for each epoch.

We noted that a particular primer set produced many positive PCR results and organized the group to disperse its effects. Five groups were randomly constructed for each PCR positive and negative results after collecting the results for each template. To divide the overall result into 5 groups, the primer-pair template data, which is the unit of data, was combined so that the total number was even for each group. When we equalize the ratio of PCR positives and negatives, the acquired data is adjusted so that the numbers are even at the stage of collecting the results for each template (undersampling).

Axlsx (https://github.com/randym/axlsx, v3.0.0) was used for colorizing spread sheets (Tables 4, 7). MatPlotLib (https://matplotlib.org/, v3.3.3) was used for creating line-graphs on epochs-accuracy (Fig. 4). GnuPlot (http://www.gnuplot.info/, v5.4) was used to create the scatter plot for Gibbs energies (Fig. 5).

**Table 4:**
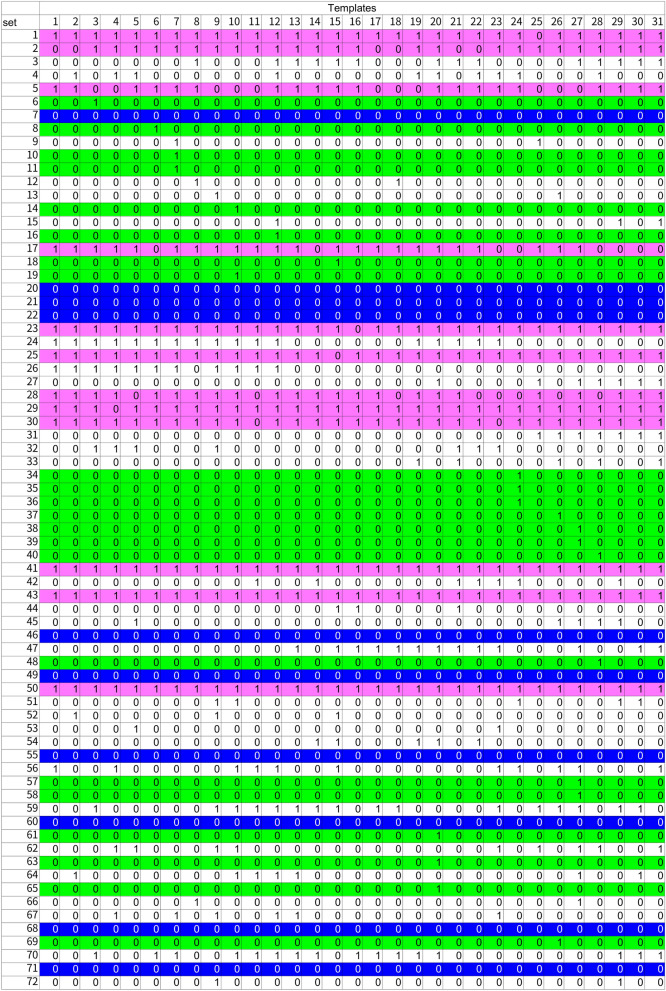
PCR results with a combination of 72 primer sets and 31 templates.

PCR results are expressed by the following numerical values based on observation on agarose gels. Number 0: No PCR product, 1: PCR product can be confirmed. Results on the primer pairs which have positive results on more than 20 templates are shown with pink background. Similarly, primer pairs which have results with single or no positive on 31 templates are shown with lime or blue background, respectively.

### Learning results

**Figure 3:**
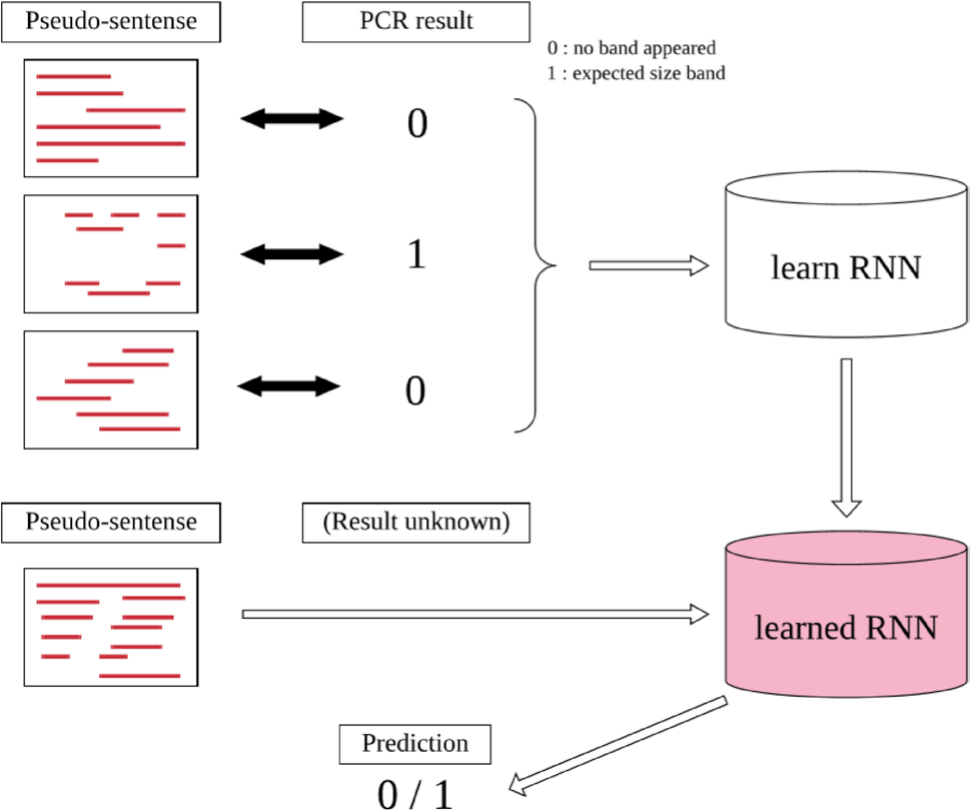
Learning and prediction by RNN. Schematic diagram of how to learn pseudo-sentences by RNN. The upper row shows the processing during learning, and the lower row shows the processing during testing. The learning results are saved in the file specified by PyTorch, read during the test, and used for prediction step.

The PCR results performed with the annealing temperatures set at 56 °C were set as pseudo-texts generated from each primer-template set and were trained by RNN. For its learning, the pseudo-sentence created for the combination of primer and template was used as input data, and the PCR results were arranged as a teacher. For the RNN, an RNN-Long short-term memory (LSTM) module of PyTorch (https://pytorch.org/, v1.7.1) was used. Python scripts for learning pseudo-sentences and extracting prediction results were written based on the scripts published in a book (Shinqiao Du, "Can be used in the field! Introduction to PyTorch development Creation of deep learning model and implementation in application", Shosuisha; 2018/9/18 in Japanese). After reading the pseudo-sentences and PCR results of each primer-pair template, RNN generated a decision algorithm that matched the output results for all input pseudo-sentences (learned algorithm) (Fig. 3). As the negative control of sentences, randomly selected nucleotide pentamers were aligned as nonsense pseudo-sentences.

The prediction accuracy of the generated trained algorithm was confirmed by split verification (cross validation). The primer pair-template sets were divided into five groups, and the RNN was learned using four groups among them and the learning. The remaining one group was not used as learning data but was utilized as verification data. Verification was made during the learning steps.

When evaluating the prediction by RNN, whether the expected PCR band was found on agarose-gel electrophoresis was treated as the true conditions, and the prediction by RNN was treated as the predictive conditions. A true positive, false negative, false positive, true negative, sensitivity, specificity, and accuracy were calculated accordingly. Significant differences in sensitivity, specificity, and accuracy between conditions were made based on Student's and Welch’s t-test^24^.

## Results

### PCR results of the primer sets and templates

PCR amplification with 72-sets of primer pairs on 31-templates was shown as 0 (no PCR amplification) or 1 (PCR product is visible) (Table 4). On 12 sets of primer pairs (Numbers 1, 2, 5, 17, 23, 25, 28–30, 41, 43 and 50), a PCR product was visible with more than 22 templates (Numbers 6, 8, 10, 11, 14, 16, 18, 19, 34–40, 48, 57, 58, 61, 63, 65 and 69). On the other hand, on 10 primer pairs (Numbers 7, 20, 21, 22, 46, 49, 55, 60, 68 and 71), no PCR product was shown. On the learning of the results, pseudo-sentences were created on each primer pair-template set. The pseudo-sentences were firstly classified by template and then randomly grouped into 5 groups to suppress the influences of a particular primer pair.

### PCR prediction by recurrent neural network (RNN)

**Figure 4:**
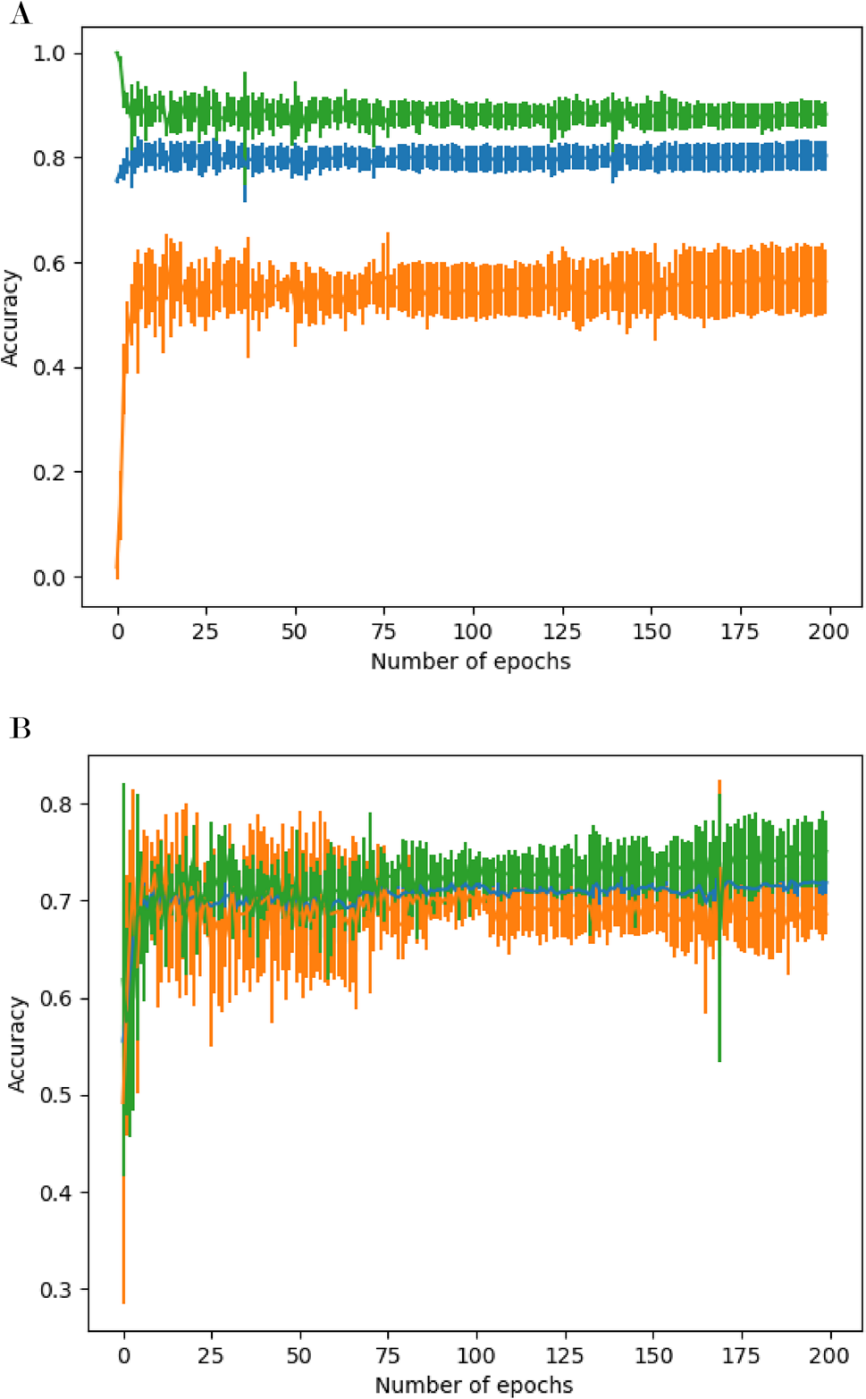
Average prediction accuracy on validation groups in cross-validation. Average of prediction accuracy was calculated on 5 validation groups in cross-validation. (**A**) Whole sets in 72-primer-31template sets were used for learning or validation. (**B**) The number of primer pair-template sets in 72-primer-31template was controlled to 1:1 by undersampling. Groups are Orange: PCR positive primer pair-template sets, Green: PCR negative primer pair-template sets, and Blue: all primer pair-template sets. Standard deviation within validation groups was shown as error bars.

As can be gleaned from the PCR results, the whole combination of primer pair and templates were divided into 5 groups (RNN-learned PCR results of 4 in 5 groups). Prediction accuracy on a verification group after learning on 4 groups was a plot against epochs (Fig. 4). Alteration of accuracies were plotted with PCR-positive, PCR-negative and all sets on whole primer pair-template sets (Fig. 4A) or undersampling sets (Fig. 4B). Since RNN predicted all sets as "negative", the prediction accuracy of PCR-negative sets was 1.0 at the start of learning. Conversely, the prediction accuracy of PCR-positive sets was 0.0 at the start of learning. After 15 epochs of learning, prediction accuracies became 0.85 and 0.58 for PCR-negative and positive sets respectively. Prediction accuracies were not much altered after 15 epochs. The accuracy remained within the standard deviation range after 200 epochs, similar to 25–200 epochs (results not shown).

For the undersampling sets, the variance between the validation groups was large from the start to 75 epochs, but after that, the variance became small, and after 100 epochs, the accuracy of the PCR-negative group became stable to be higher than those of PCR-positive groups (Fig. 4B).

### Sensitivity of learned RNN

The RNN used four of the five divided groups for learning and one group as validation. Since these validation groups alternate sequentially, when the validations for the five types of learning are combined, all the data used for the RNN became one cross table (Table 5A). When all the data were used for learning, the number of the PCR negative and positive sets were 1678 and 554, respectively. From this cross table, sensitivity, specificity, and accuracy were calculated to 0.56, 0.88, and 0.80, respectively. In the same manner, sensitivity, specificity, and accuracy were calculated to 0.71, 0.73, and 0.72, respectively when the number of negative and positive samples was adjusted to be the same (Table 5B).

**Table 5:** Cross table of PCR results and predictions.

Prediction on RNN	PCR-result	
	Negative	Positive
**A. Validation of whole data**		
Negative	1481	242
Positive	197	312
**B. Validation of undersampling data**		
Negative	380	174
Positive	138	416
**C. Cross table on the test data predicted by whole data-learned RNN**		
Negative	1419	73
Positive	176	6
**D. Cross table on the test data predicted by whole data-learned RNN**		
Negative	1051	42
Positive	544	37

Cross tables on PCR-results and RNN-predictions are made on the RNN-predictions on 200-epochs. Set numbers on RNN-prediction of the test data (C, D) were shown on each learned group on which prediction algorithm was created by learning. Data from prediction on group 1 is shown on C and D, average and standard deviations on prediction from 5 groups are listed in Table 6.

In the division verification, sensitivity, specificity, and accuracy were calculated for the validation data in each division group. On the test data, the prediction was performed by learned RNN on each division verification. Thus, there were 5 predictions and sensitivity, specificity, and accuracy on test data. As a result, 5 sets of data were obtained under each condition, and a significant difference could be detected between those data (Table 6). In comparing whole-data and undersampling predictions, the sensitivity was significantly higher with undersampling. Moreover, in the specificity of undersampling, data were lower than those of whole data. Similarly, in the test, the sensitivity was significantly higher in the undersampling data, and the specificity was significantly higher in the whole data. These results suggest that the prediction rate of PCR-positives decreases when the number of PCR negative sets is large in RNN learning. In the test sets, the particularly low sensitivity is seen in prediction using whole-data-learned RNN (Table 5C). These results suggest that the current prediction method may depend on the base sequence of the primer itself. Thus, the coding method described in this study may not be perfectly suitable for predicting PCR-positive results. On the other hand, an increase in the possibility of detecting PCR positive through undersampling-data-learned RNN has indicated that prediction also depends on the number of negative and positive samples during learning (Table 5D).

**Table 6:** Sensitivity, specificity and accuracy in split verification data and test data by learned RNN.

	Sensitivity	Specificity	Accuracy
**Validation**			
Whole data	0.563 ± 0.062	0.882 ± 0.024^1^	0.803 ± 0.029^1^
Undersampling	0.751 ± 0.031^2^	0.686 ± 0.021^3^	0.718 ± 0.022^2^
**Test**			
Whole data	0.114 ± 0.092	0.899 ± 0.022^1^	0.862 ± 0.020^1^
Undersampling	0.471 ± 0.092	0.661 ± 0.056^3^	0.652 ± 0.052^3^

Sensitivity, specificity and accuracy were calculated for RNN predictions and PCR results from 5 split validation groups for the whole data and undersampling sets respectively. For the Test data, the mean value and standard deviation were calculated for the results of prediction by the RNN that independently learned with each verification group. Numbers on superscript show groups in which no significant difference are detected. On the other combinations, significant differences were shown with the Student's and Welch’s t-tests.

### Color summarization of prediction and result

To depict how PCR was predicted with individual primer-template combinations, we colored and displayed the individual PCR results as shown in Table 3 concerning the PCR-result and predictions in validation-data (Table 7). In this color display, no template strongly affected the prediction. On the other hand, several primer pairs suggested affecting PCR prediction (primer set numbers 17, 23, 41, and 43) (Table 7A). On the other hand, primer pair numbers 5, 24, 26, 50 and 70 showed relatively low accuracies through the templates. For this primer pair, it is suggested that RNN did not use much of this primer set information when making a prediction.

**Table 7:**
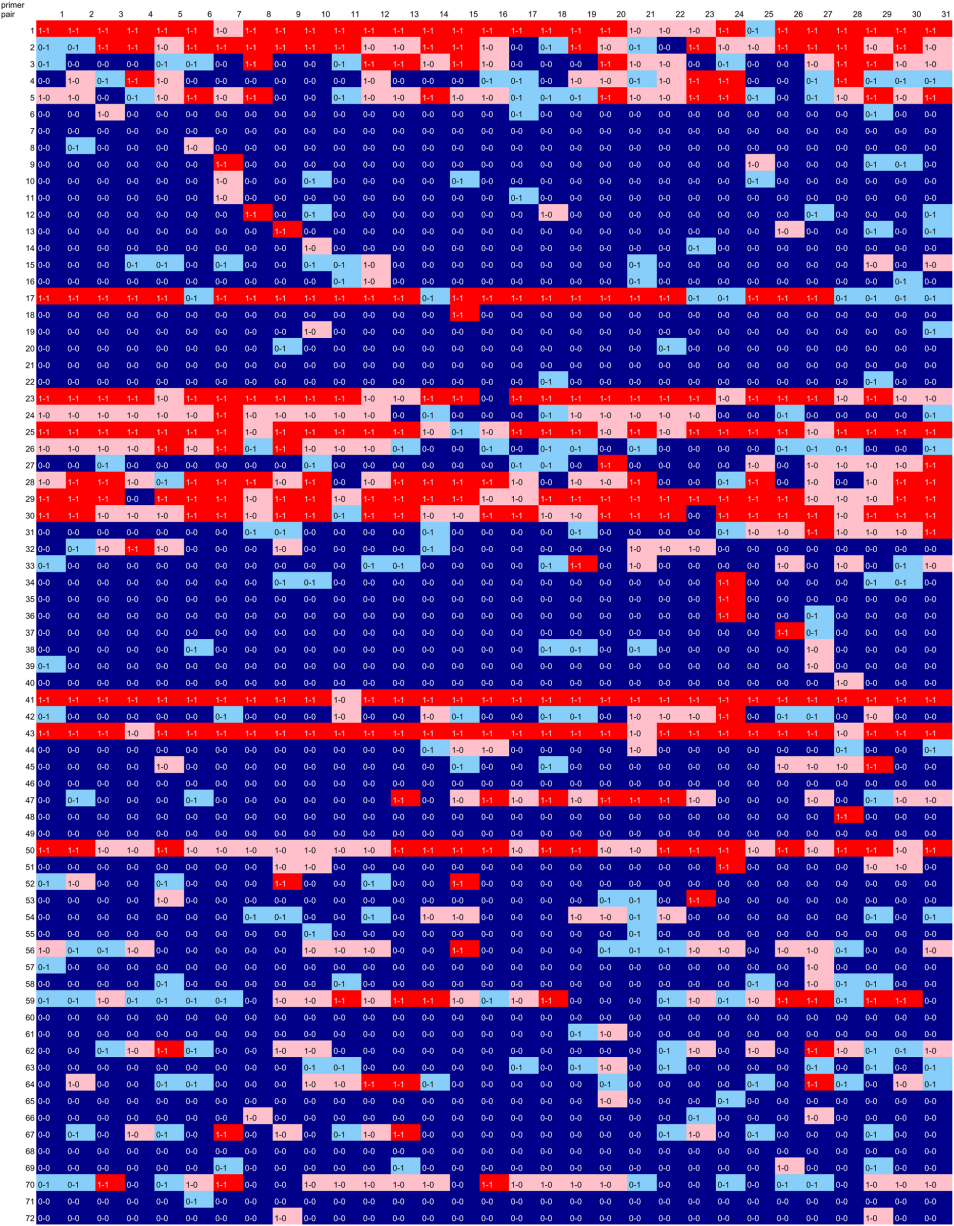

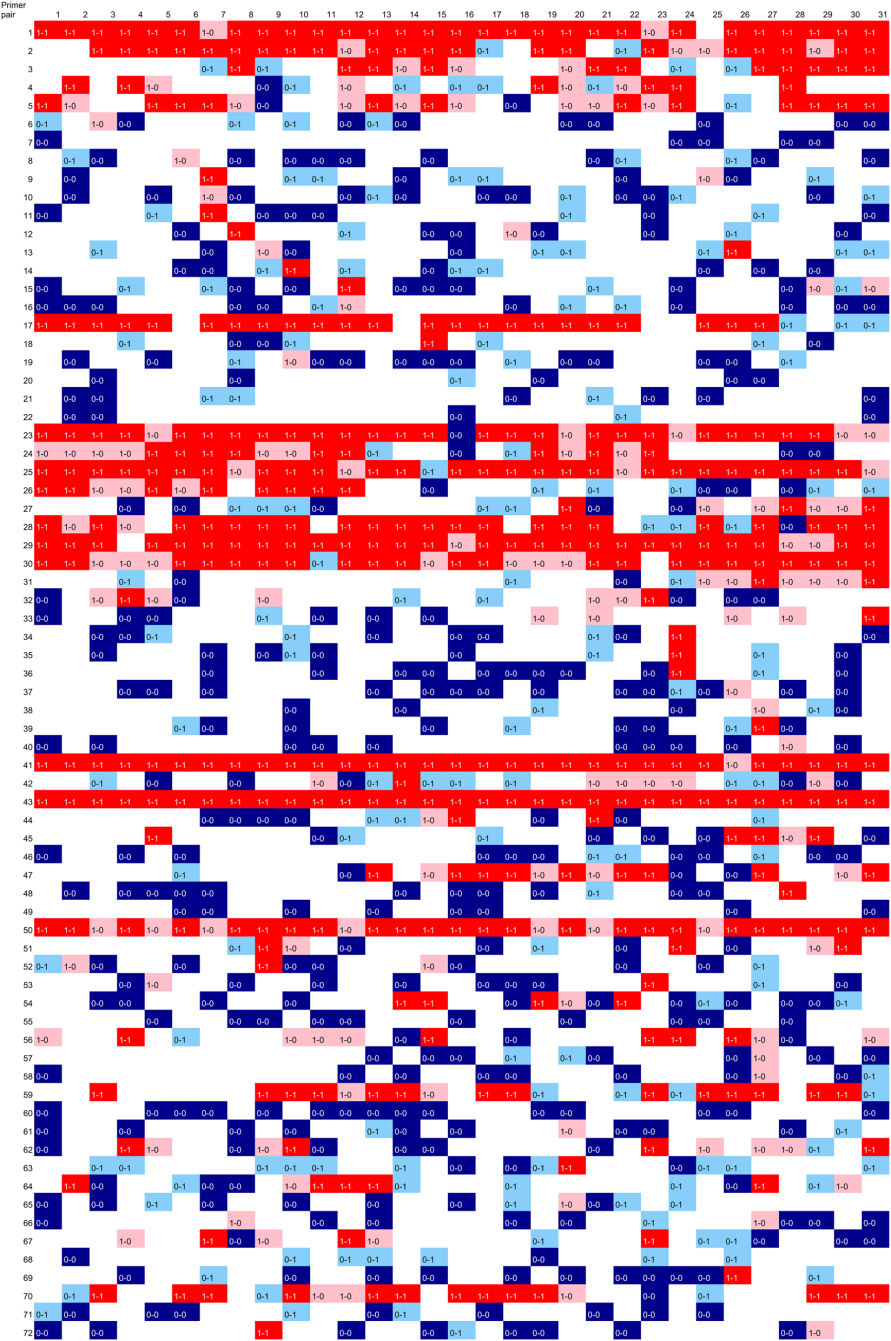
Color summarization of PCR results and predictions, (A) predictions from whole-data, (B) predictions from undersampling data.

Color presentations for PCR-result and RNN-prediction. Colors show the following results and predictions; red: PCR-positive-prediction-positive, pink: PCR-positive-prediction-negative, blue: PCR-negative-prediction-negative, light blue: PCR-negative-prediction-positive, white: primer pair-template sets excluded from predictions during undersampling.

In undersampling data, two-thirds of the negatives were excluded from the prediction, so white squares are shown (Table 7B). For primer pairs that were PCR positive for many templates in this group, RNNs were often predicted to be correctly positive. From this result, it was suggested that RNNs whose degree of positive learning was increased by undersampling.

### Gibbs scatter plot

**Figure 5:**
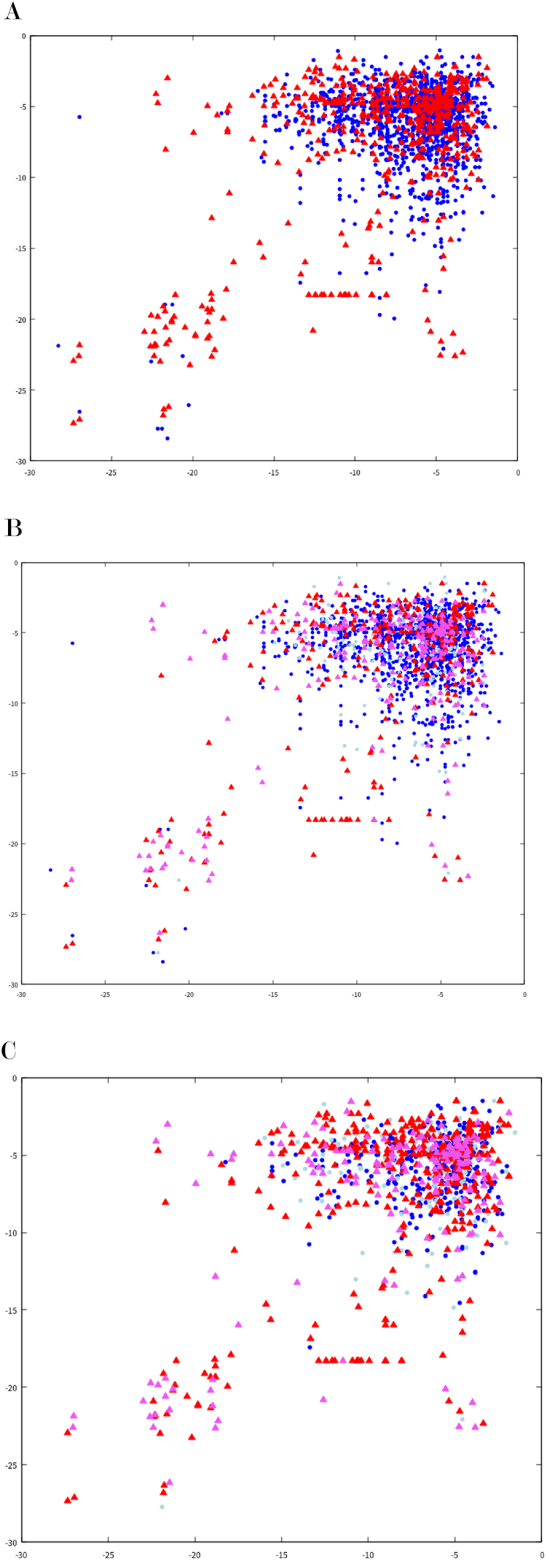
Scatter plot of PCR-results and predictions. Plot the PCR results and RNN-predictions against the Gibbs energy at the hydrogen bond at the forward (horizontal axis) and reverse (vertical axis) priming positions determined at the time of pseudo-sentence determination (Fig. 2). Colors and shape show the following results and predictions; red triangle: PCR-positive-prediction-positive, pink triangle: PCR-positive-prediction-negative, blue circle: PCR-negative-prediction-negative, light blue circle: PCR-negative-prediction-positive. Primer pair-template sets excluded from predictions during undersampling were not plotted.

When we created the pseudo-word, binding to template at the 3′end of the primer sequence was recorded as the binding of the primer that could develop into PCR and used for prediction. At that time, the Gibbs energy was calculated for most of the bonds to the template at the 3′end, and the primer at the position where the Gibbs energy was low and the PCR product was produced was used as a predictive primer-position for PCR prediction. Using this result, a scatter plot was created for forward and reverse with the assumed primer binding Gibbs energy on the horizontal and vertical axes (Fig. 5). When the set data with a positive PCR result is marked with a red triangle and those with a negative PCR result is marked with a blue circle, the set with the full length of the primer homologous to the template plot in the lower left. The set with the only weak binding plot in the upper right displayed (Fig. 5A). We predicted that PCR would occur only with strong interactions in the lower left region and not with weak interactions in the upper right region. While, in our PCR experiments, many PCR positives were found in the upper right region.

We also showed the prediction results were superimposed the Gibbs plot on the PCR-results (Fig. 5B,C). The primer pair-template sets for which the PCR-positive-RNN-negative result shown in pink were found not only in the upper right region but also in the lower left region in a similar ratio. If the prediction is based on the strength of hydrogen bonds, the prediction accuracy in the lower left region is expected to be high, but the prediction in this study did not show such a tendency. Even for the undersampling data with improved PCR-positive prediction accuracy, no improvement in prediction accuracy was observed in the lower left region (Fig. 5C).

Regarding the prediction of PCR-negative, many prediction errors related to PCR negative were observed in the peripheral region where the Gibbs energy of sets were slightly lower than those in the most upper right region (Fig. 5B). This trend did not change with the undersampling data (Fig. 5C). We expected that the PCR-negative prediction would use the same mechanism as the PCR-positive prediction, but in the lower left region of the whole sample prediction, the RNN correctly predicted several PCR-negatives in the lower left region (Fig. 5B). At the time of undersampling, these sets were not selected on the random selection and did not plotted (Fig. 5C). Considering that the prediction accuracy for PCR-positive in this region was not high, it is suggested that the PCR-negative prediction in the lower left region uses different criteria from the positive prediction.

## Discussion

PCR is one of the basic technologies commonly utilized for genetic as well as pathogen-detection testing^25,26^. Because of its declining cost, determining the base sequence of DNA or RNA subjected to PCR has now considerably increased^27–29^. Furthermore, the development of applied technologies such as real-time and droplet PCRs and the application range of PCR has been expanded even further^30–33^. When PCR is used to detect pathogens, specific detection is required. Moreover, such consideration for specific detection can be affected by some base sequence contaminants in processed samples. It is expected that such cases will likely increase if not rectified.

One of the strengths of PCR is that once a DNA is known, a highly sensitive test or method^34^ can be developed. This can be applied to various test targets over a very short period. It means that a highly sensitive detection becomes possible in a shorter time compared with developing an immunological test or technique. The only disadvantage of PCR method is, when there is a similar sequence between them, there is always a possibility that non-specific bands may be generated^35^. This can happen as in the case of distinguishing bacteria by targeting a specific molecule that is contained only in ribosomal RNA. In this instance, it is difficult to design primers that enable specific detection because 16s ribosomal RNAs can have similar base sequences with each other^36–39^. Thus, a test is required in the presence of a similar nucleotide sequence such as when a specific pathogen is found in a sample in which many other species are mixed.

Major parts of PCR primer design technologies were almost completed in the 1990s^40^. The primer design technique is based on the stability of the hydrogen bond between the primer and the template based on the nucleotide sequence, and the PCR experiments conducted while examining its stability. Its hydrogen bond stability can be predicted by the free energy calculated from enthalpy, entropy, and absolute temperature^41^. Early basic experiments^42^ have proven that one base at 3′ greatly affects the PCR reaction, and primers are designed based on 3′ several bases. Software for verifying the easiness of primer application and for designing primers by extracting the susceptible base sequence from the target base sequence was also developed^5,6,43^. This primer design software, especially the Primer3, has a very large tracking record. Primers designed with Primer3 can amplify target DNA with an accuracy of 80% to 90%. However, even if the conventional primer design algorithm can design the primer that is most likely to cause PCR in the target template, it does not predict the amplification in the template DNA other than the intended one present in the sample. In our preliminary experiment, several Primer3-designed primer pairs amplify all 16sRNA templates regardless of the target DNA on the design of primers. Therefore, for a design of a primer pair that amplifies only the target template with the existence of similar sequences, it is necessary to consider a method different from the previous optimum design.

In the design of PCR primers, it is difficult to compare primer sets relative to each other by the method of selecting the optimum set. When selecting the optimum primer with Primer3 etc., 30 or more indexes are calculated, but a formula that uniformly shows the relationship between those indexes and the actual PCR is not provided^7^. It is expected that various DNAs in PCR tubes, including templates and primers, and PCR reaction conditions will contribute to the PCR results in different proportions under each condition. For example, the 3′end of the primer is known to have a very large effect on PCR with just a few bases. Although DOP-PCR and similar arbitrary methods are known to nonspecifically amplify a wide range of DNA by matching several bases^44^. Experiments in the artificial gene synthesis from oligomers have also suggested that the primers are easily elongated when they form dimers^21^. Not only the binding position of the primer but also the base sequence of the PCR target region may have an influence depending on the annealing temperature. Of course, the base sequences of the primers and templates themselves also affect the results as factors other than mere stability. Thus, to design a primer that performs PCR only on a specific template, not on similar template sequences, it is necessary to consider the unknown number of factors without information about any contribution.

In recent years, supervised machine learning^14^ has been developed as a method of making predictions without determining the number or combination of factors that contribute to the results. In this method, after preparing data with correct answers, a large number of perceptrons are connected (perceptron network), and the serial adjustment of connection is optimized to form the perceptron network with the highest accuracy rate^14–17^. Since the substance of the prediction is a set of coefficients of the perceptron and its network, it is not necessary to analyze the factors for increasing the accuracy rate. Instead, analyzing learned machine learning often does not reveal the factors. Based on the discussion in the previous paragraph, it was expected that supervised machine learning would be suitable, as it does not require the number or combination of factors that contribute to the results to predict the success or failure of PCR.

In this study, PCR results were predicted from the base sequences of primers and templates using natural language processing that examines text trends. The PCR reaction is affected not by the base sequence of the primer or the template alone, but by the combination of complementary strands when they form a complementary strand. Therefore, we decided to generate the code from a combination of PCR primer pairs and complementary strand bases formed by the template. The generated code was split into words so that a sentence was formed from a set of primers and templates. Since a sentence can be created for each primer set and template, if there is a PCR experimental result, the PCR experimental result can be linked as a correct answer to each sentence. In natural language processing, a machine-learning network is made to learn a sentence whose evaluation is confirmed, and the learned network predicts an unidentified sentence. In RNN, which is a typical natural language processing machine learning, RNN is trained in movie criticisms with positive evaluation and negative movie criticism, and the evaluation is predicted for unidentified movie criticism^6,45,46^. By generating pseudo-sentences using the primer set and template proposed in this paper as a unit, it is possible to associate PCR results with each pseudo-sentence in the same way as Positive/Negative in film criticism. Since the generation of pseudo-words from the complement set alone could not reflect that the complementarity of the 3′end was greater than that of the 5′end, it was emphasized as a word iteration. Therefore, for the learning of pseudo-sentences in this study, the same RNN as the one learned for the evaluation of film criticism was used. This is the first paper to use a neural network application to design primers and predict PCR results. Supervised machine learning was used to learn the PCR results. Since we created pseudo-words and pseudo-sentences as input information, we selected RNNs to learn the relationship between primer and template sequences and PCR results. RNNs can interpret sentences while analyzing the context of words in the sentence. In this study, in a test experiment conducted by actually creating a new primer, prediction was made with an accuracy of 70% or more (Table 5). These results suggest that the interaction between the primer and the template is also effective when the interpreted data of the RNN is returned to the previous layer and used for further interpretation. They also suggest that the effect of primer-template interaction on PCR is similar to the effect of natural language word placement in semantic interpretation. The LSTM used the word context in the sentence to change the retention of the word's effect for each word and make a comprehensive judgment of its effectiveness^47,48^.

We created our pseudo-words for RNN analysis for this study (Fig. 2). All of the letters that make up a word were determined based on the primer-template interactions that are important in previous studies (Fig. 2E). Natural language processing by RNN uses all the words used in a specific language, so the vocabulary is about 30 to 100,000 words (RNN literature). In this study, the data was as small as 2,000, so it was necessary to have a small vocabulary. Therefore, the original 16 base combinations are summarized in 5 based on the effect of Taq polymerase on DNA synthesis. However, considering that the primers face each other in the opposite direction during PCR, the direction of homology was reflected in the letters. Besides, different character sets were prepared for dimers and hairpins. Also, uppercase and lowercase letters were set for the evaluation target as the starting point of PCR and other parts. As a result, the vocabulary of the 5-letter pseudoword (pentacode) code was 5 to the 5th power × 5 × 2 = 31,250. In RNN, the characteristics of each sentence are expressed by the amount of words used (word vector) with the vocabulary as the number of dimensions. If the vocabulary is large, the frequency of occurrence of words is low, so the word vector becomes a sparse vector and may not sufficiently show the characteristics of the sentence. On the other hand, when the size of the vocabulary is small, detailed features may not be expressed, which suggests that the prediction accuracy is limited. In the method of this study, the number of characters in a word was shortened to 5 as another method to reduce the size of the vocabulary. It is suggested that extending this to 6 or 7 bases will increase the vocabulary and enable more accurate predictions. In the future, it is thought that this code setting method can be improved by accumulating more data.

In this study, pseudo-words were created based on primer hairpins, dimers, primer-template homology, and primer-PCR product homology. Predicting the priming position is expected to be particularly important among pseudo-words. This is because PCR is established based on the elongation of DNA from the priming position (Fig. 1). When designing the optimum primer as in the conventional case, the binding position of the primer has a long complementary region and high stability as compared with other positions. However, when comparing the complementarity between the template and the primer sequence, which was not originally designed, it is necessary to determine the priming position from a large number of candidates having similar length and stability of the complementary strand. Also, the effect of priming position was conveyed by expressing the priming position in capital letters. The accuracy of this pumping position affects the accuracy of the overall prediction, whereas, in addition to the complementarity with the base sequence and template of the primer, it becomes an amplified sequence or set (reverse for forward, forward for reverse). Thus, its relationship with the priming position is also affected. Therefore, it is ideally desirable to learn and predict this priming position by artificial intelligence. However, since the basic data is not available in this study, the stability of the complementary strand is predicted by the nearest neighbor method. The priming position that maximizes stability was predicted with the set of priming positions. For the prediction of free energy by the nearest neighbor method, in addition to the values reported so far, values extrapolated from those values were set and used. Since some of these numbers are simple extrapolations from the reported numbers, their accuracy is not yet guaranteed, hence, future improvements are still needed.

Improvement in prediction accuracy in RNN is enhanced in the process of repeating epochs (Fig. 4). When all the data were used, the prediction was stable at about 25 epochs, and no significant change occurred. After which, when the number of PCR positive and negative data was matched by undersampling, the error was up to 75 epochs larger. Later transition period of up to 100 epochs made the prediction accuracy become stable. This indicates that the structure of data affects the learning steps of RNN. When the number of data or composition is changed in the future, we proposed to first investigate the changes in epoch and prediction accuracy.

The PCR results used in this study include those that were greatly influenced by primer pairs (Table 4). In 12 of 72 primer pairs, PCR was observed in 20 or more of 31 templates. In 22 primer pairs, PCR was observed in only 1 template. No PCR was observed for 10 primer pairs. Perspectively, these primer pair-template data combinations showed that the predictions were relatively correct when only one of the templates was amplified or when PCR was not applied to any of the templates (Table 7A). This suggests that PCR was successful to primers with high specificity, and conversely, RNNs made highly accurate predictions for primer sets with low PCR characteristics. On the other hand, in the primer pairs in which PCR was observed in a large number of templates, the prediction was relatively wrong, suggesting that it was difficult to predict the RNN for such primers in where false positives frequently appear. The relationship between primer binding to the template and prediction is shown in a scatter plot made with Gibbs energy at the optimal binding position of the primer (Fig. 5). In this scatter plot, the primer and template set specifically designed for lower left area are shown, and the results for the primer pair and template set that do not assume PCR are shown in the upper right region (Fig. 5A). Surprisingly, the prediction did not always hit lower left region, but to the same extent in the upper right (Fig. 5B,C). This tendency was the same for undersampling, suggesting that improvement in prediction accuracy for PCR positive was influenced by improvement in the accuracy rate in the upper right region. For PCR-negative predictions, it is noteworthy that the RNN hit the predictions for multiple PCR-negative sets in the lower left region of the scatter plot created from the predictions of all the data. These results show that the RNN described in this study does not have high accuracy at present, but it is expected that the prediction accuracy will be improved by improving the number of data and reviewing pseudo-words in the future.

It is challenging for RNNs to simplify which of the pseudo-words and their repetitions can have a great influence on the characteristics of supervised machine learning. The correctness of the prediction does not guarantee the correctness of the setting like the pseudo-word. Moreover, through this paper, researchers may now find it useful to reconstruct the prediction method. Pseudo-word generation and pseudo-sentence prediction do not provide the theoretical justification of algorithms based on unified theory, but databased reproducibility can be provided to the user.

In conclusion, it is indicated that PCR design by natural language processing system using RNN be utilized in enabling a primer design to detect a specific template in the presence of multiple templates. Method accuracy is improved by learning the base sequence of the primer pair, the template, and the PCR result. Design can be upgraded by using discarded negative data.

## Supplementary Information

Supplementary Information 1.

Supplementary Information 2.

Supplementary Information 3.
