# Supplementary material for: Prediction of PCR amplification from primer and template sequences using recurrent neural network
Source: Sci Rep. 2021 Apr 5;11:7493. doi: 10.1038/s41598-021-86357-1 (PMC8021588; doi:10.1038/s41598-021-86357-1)
Supplement: Supplementary file 1 — Supplementary Information 1. [file 41598_2021_86357_MOESM1_ESM.docx]

Supplement 1 for “Prediction of PCR amplification from Primer and Template Sequences using Recurrent Neural Network” by Kotetsu Kayama, Miyuki Kanno, Naoto Chisaki, Misaki Tanaka, Reika Yao, Kiwamu Hanazono, Gerry Amor Camer and Daiji Endoh

Data for templetes used in the experiments.

Table 1 Template name, nucleotide sequence, nucleotide sequence length, 16S rRNA sequence and accession number

| Template Number | Phylum (or group) | Nucleic Acid Sequence | Size | Accession | Range for template |
| --- | --- | --- | --- | --- | --- |
| 1 | Actinobacteria | CGTGTCGTGAGATGTTGGGTTAAGTCCCGCAACGAGCGCAACCCTTGTCTTATGTTGCCAGCACACTWTGGTGGGGACTCATGAGAGACTGCCGGGGTTAACTCGGAGGAAGGTGGGGATGACGTCAAATCATCATGCCCCTTATGTCCAGGGCTTCACACATGCTACAATGGTCGGTACAACGCGCTGCGAGCCTGTGAGGGTGAGCGAATCGCTGAAAGCCGGCCTCAGTTCGGATTGGGGTCTGCAACTCGACCCCATGAAGTCGGAGTCGCTAGTAATCGCAGATCAGCAACGCTGCGGTGAATACGTTCCCGGGCCTTGTACACACCGCCCGTCAAGTCACGAAAGTTGGTAACACCCGAAGCCAGTGGCCTAACCCTTGTGGAGGGAGCTGTCGAAGGTGGGATCGGCGATTGGGACTAAGTCGTAACAAGGTAGCCGTACCGGAAGGTGCGGCTGGATCACCTCCTTT | 475 | NR_121753.2 | 1048 - 1522 |
| 2 | Spirochaetes | TTGGGTTAAGTCCCGCAACGAGCGCAACCCTCACCTTATGTTGCCATCATTCAGTTGGGCACTCGTAAGGAACTGCCGGTGACAAACCGGAGGAAGGCGGGGATGACGTCAAATCCTCATGGCCTTTATGTCTAGGGCAACACACGTGCTACAATGGCCGGTACAAAGGGTAGCCAACTCGCGAGGGGGAGCTAATCTCAAAAATCCGGTCCCAGTTCGGATTGGAGTCTGCAACTCGACTCCATGAAGTCGGAATCGCTAGTAATCGCGGATCAGCATGCCGCGGTGAATACGTTCCCGGACCTTGTACACACCGCCCGTCACACCACCTGAGTGGGGAGCACCCGAAGTGGTCTTTGCCAACCGCAAGGAAGCAGACTACTAAGGTGAAACTCGTGAAGGGGGTGAAGTCGTAACAAGGTAGCCGTATCGGAAGGTGC | 440 | NR_116542.1 | 1025 - 1464 |
| 3 | Synergistetes | GTCGTGAGATGTTGGGTTAAGTCCCGCAACGAGCGCAACCCCTGCGCCTAGTTGCCATCAGTTAGGCTGGGCACTCTAGGCGGACTGCCGGCGACAAGTCGGAGGAAGGTGGGGATGACGTCAAGTCATCATGGCCTTTAAGCCCAGGGCGACACACGTGCTACAATGGCCAGCACAGAGGGCTGCAAGTCCGCGAGGACAAGCGAATCCCTTAAAGCTGGTCTCAGTTCGGATTGCAGTCTGCAACTCGACTGCATGAAGCCGGAATCGCTAGTAATCGCCGGTCAGCCATACGGCGGTGAATACGTTCCCGGGCCTTGTACACACCGCCCGTCACACCACCCGAGTTGGGTGCTCCCGAAGCCGCCGGCCCAACCCCGTAAGGGGAGGGAGGCGTCGAAGGAGTGTCTGATAAGGGGGGTGAAGTCGTAACAAGGTAGCCGTACCGGAAGGTGCGGCTGGATCACCT | 469 | NR_102954.1 | 1051 - 1519 |
| 4 | Dictyoglomi | TGGAGCCTGCACAGGTGGTGCATGGCTGTCGTCAGCTCGTGTCGTGAGATGTTGGGTTAAGTCCCGCAACGAGCGCAACCCCTGCCCTTAGTTGCCAGCGGGTAAAGCCGGGCACTCTAAGGGGACTGCCGGCGAAGAGCCGGAGGAAGGTGGGGATGACGTCAAGTCAGTATGCCCCTTATGCCCTGGGCTACACACGCGCTACAATGGGTGGTACAGAGGGGAGCGAAGCCGCGAGGCGGAGCGAATCCCTAAAGCCACCCCCAGTTCAGATCGCAGGCTGCAACTCGCCTGCGTGAAGGCGGAATCGCTAGTAACCGCAGATCAGCCACGCTGCGGTGAATACGTTCTCGGGCCTTGTACACACCGCCCGTCACACCACGAGAGTCCGCAACACCCGAAGTCAGGCGAAGAGCCTGCCGAAGGTGGGGCGGATGATTGGGGTGAAGTCGTAACAAGGTAGCCGTACCGGAAGGTGCGG | 481 | NR_074876.1 | 1044 - 1524 |
| 5 | Thermodesulfobacteria | TGCCTGGGAGCCCTAGCACAGGTGCTGCATGGCTGTCGTCAGCTCGTGTCGTGAGATGTTGGGTTAAGTCCCGCAACGAGCGCAACCCTTGCCCTTAGTTGCCAGCGGGTAGAGCCGGGCACTCTAGGGGGACTGCCGGGGACAACCCGGAGGAAGGGGGGGATGACGTCAAGTCATCATGGCCCTTATGCCCAGGGCTACACACGTGCTACAATGGGCGGTACAGAGGGAAGCGAACCCGTAAGGGGGAGCAAATCCCAGAAAGCCGCTCTCAGTACGGATCGGGGTCTGCAACTCGACCCCGTGAAGCCGGAATCGCTAGTAACGGCGGATCAGCATGCCGCCGTGAATACGTTCCCGGGCCTTGTACACACCGCCCGTCACACCACGGGAGCTGGCTCTGCCCGAAGTCGCTATCCCAACCCCCGGAAGGGGGAGGGAGGCGCCGACGGCAGGGCTGGTGACTGGGGTGAAGTCGTAA | 481 | NR_075021.1 | 1045 - 1525 |
| 6 | Elusimicrobia | GTCGTCAGCTCGTGTCGTGAGATGTTGGGTTAAGTCCCGCAACGAGCGCAACCCCTATTCTGTGTTGCCTAGCAATAGGATCTCTCAGAAGACTGCCGCGGATAACGTGGAGGAAGGTGGGGATGACGTCAAATCATCATGGCCTTTATGTCCAGGGCTACACACGTAATACAATGGCATAGACAGAGGGCAGCAATATCGCGAGATGGAGCCAATCCCTAAACTATGCCCCAGTTCAGATTGCAGGCTGCAATTCGCCTGCATGAAGCCGGAATCTCTAGTAATCGCAGATCAGCACGCTGCGGTGAATACGTTCCCGGGTCTTGTACACACCGCCCGTCACACCACGAAAGTTAATTGCAACAGAAGTGCTCAGGTCGTCTGGGCCCTAAGTTG | 396 | NR_115046.1 | 1000 - 1395 |
| 7 | Chlorobi | GTGTCGTGAGATGTTGGGTTAAGTCCCGCAACGAGCGCAACCCCTACAATTAGTTACTAACAGGTTAAGCTGAGGACTCTAATTGAACTGCCTACGCAAGTAGTGAGGAAGGAGGGGATGACGTCAAGTCCTCATGGCCCTTACGCCCAGGGCCACACACGTGATACAATGGTAGCTACAGAGGGCAAAGCCGCGAGGCAGAGGAAATCCCAAAAAAGCTATCTCAGTCCGGATCGGAGTCTGCAACTCGACTCCGTGAAGTTGGAATCGCTAGTAATCGCAGATCAGCACGCTGCGGTGAATGTGTTCCCGGGCCTTGTACACACCGCCCGTCAAGTCATGGAAGTCAGGAGTACCCAAAGACGCTCGCGCGTTTAAGGTAAGACTGGTAACTGGGACTAAGTCGTAACAAGGTAGCCGTACCGGAAGGTGCGGCTGGATCACCTCCTTT | 451 | NR_074355.1 | 1044 - 1494 |
| 8 | Verrucomicrobia | AGATGTTTGGTTAAGTCCAGCAACGAGCGCAACCCCTGTTGCCAGTTACCAGCACGTGAAGGTGGGGACTCTGGCGAGACTGCCCAGATCAACTGGGAGGAAGGTGGGGACGACGTCAGGTCAGTATGGCCCTTATGCCCAGGGCTGCACACGTACTACAATGCCCAGTACAGAGGGGGCCGAAGCCGCGAGGCGGAGGAAATCCTAAAAACTGGGCCCAGTTCGGACTGTAGGCTGCAACCCGCCTACACGAAGCCGGAATCGCTAGTAATGGCGCATCAGCTACGGCGCCGTGAATACGTTCCCGGGTCTTGTACACACCGCCCGTCACATCATGGAAGCCGGTCGCACCCGAAGTATCTGAAGCCAACCGCAAGGAGGCAGGGTCCTAAGGTGAGACTGGTAACTGGGATGAAGTCGTAACAAGGTAGCCGTAGGGGAACCTGCGGCTGGATCACCTCCTTT | 465 | NR_074436.1 | 1041 - 1505 |
| 9 | Deinococcus-Thermus | CTCGTGCCGTGAGGTGTTGGGTTAAGTCCCGCAACGAGCGCAACCCCCGCCGTTAGTTGCCAGCGGTTCGGCCGGGCACTCTAACGGGACTGCCCGCGAAAGCGGGAGGAAGGAGGGGACGACGTCTGGTCAGCATGGCCCTTACGGCCTGGGCGACACACGTGCTACAATGCCCTACAAAGCGATGCCACCCGGCAACGGGGAGCTAATCGCAAAAAGGTGGGCCCAGTTCGGATTGGGGTCTGCAACCCGACCCCATGAAGCCGGAATCGCTAGTAATCGCGGATCAGCCATGCCGCGGTGAATACGTTCCCGGGCCTTGTACACACCGCCCGTCACGCCATGGGAGCGGGCTCTACCCGAAGTCGCCGGGAGCCTACGGGCAGGCGCCGAGGGTAGGGCCCGTGACTGGGGCGAAGTCGTAACAAGGTAGCTGTACCGGAAGGTGCGGCTGGATCACCTCCTTT | 467 | NR_037066.1 | 1049 - 1515 |
| 10 | Ignavibacteriae | TGGCTGTCGTCAGCTCGTGCCGTGAGGTGTTGGGTTAAGTCCCGCAACGAGCGCAACCCCTACCATTAGTTGCCATCAGGTTAAGCTGGGCACTCTAATGGGACTGCCTACGCAAGTAGTGAGGAAGGTGGGGATGACGTCAAGTCAGCATGGCCCTTACGCCTAGGGCTACACACGTGCTACAATGGGTGCTACAACGGGTAGCGAAACCGCGAGGTGGAGCCAATCCCTAAAAAGCATCCTCAGTTCGGATTGGAGTCTGCAACCCGACTCCATGAAGCTGGAATTGCTAGTAATCGCGCATCAGCACGGCGCGGTGAATACGTTCCCGGGCCTTGTACACACCGCCCGTCAAGCCATGGAAGCCGGGGGTACCCGAAGTCAGTGACCCAACTCCGCCTCGGCGGAGAGGGAGCTGCCGAAGGTAAAACCGGTGACTGGGGCTAAGTCGTAACAAGGTAGCCGTACCGGAAGGTGCGGC | 481 | NR_074796.1 | 1045 - 1525 |
| 11 | Gemmatimonadetes | GTGTCGTGAGATGTTGGGTTAAGTCCCGCAACGAGCGCAACCCTTGCCCTTAGTTACCAGCGAGTAAAGTCGGGGACTCTAGGGGGACTGCCGGTGCCAAACCGGAGGAAGGTGGGGACGACGTCAAGTCATCATGGTCCTTACGTCTGGGGCTACACACGTGCTACAATGGCCGGTACAGAGGGCTGCGAAAGAGCAATCTGGAGCCAATCCCTAAAGCCGGCCTCAGTTCGGATTGTCGTCTGCAACTCGACGGCATGAAGCTGGAATCGCTAGTAATCGCGGATCAGCGACGCCGCGGTGAATACGTTCCCGGGCCTTGTACACACCGCCCGTCACGCCATGGAAGCTGTGAGCGCCCGAAGTCGGTGCAGGAACCCGCAAGGGGCCAAGCCGCCTAAGGCGAGCGCAGTGACTGGGGCGAAGTCGTAACAAGGTAGCCGTAGGGGAACCTGCGGCTGGATCACCTCCTTT | 474 | NR_074708.2 | 1050 - 1523 |
| 12 | Proteobacteria | CTGCATGGCTGTCGTCAGCTCGTGTTGTGAAATGTTGGGTTAAGTCCCGCAACGAGCGCAACCCTTATCCTTTGTTGCCAGCGGTTAGGCCGGGAACTCAAAGGAGACTGCCAGTGATAAACTGGAGGAAGGTGGGGATGACGTCAAGTCATCATGGCCCTTACGAGTAGGGCTACACACGTGCTACAATGGCATATACAAAGAGAAGCGACCTCGCGAGAGCAAGCGGACCTCATAAAGTATGTCGTAGTCCGGATTGGAGTCTGCAACTCGACTCCATGAAGTCGGAATCGCTAGTAATCGTGGATCAGAATGCCACGGTGAATACGTTCCCGGGCCTTGTACACACCGCCCGTCACACCATGGGAGTGGGTTGCAAAAGAAGTAGGTAGCTTAACCTTCGGGAGGGCGCTTACCACTTTGTGATTCATGACTGGGGTGAAGTCGTAACAAGGTAACCGTAGGGGAACCTGCGGTTGGA | 481 | NR_156052.1 | 844 - 1324 |
| 13 | Firmicutes (Bacillus) | GTGACAGGTGGTGCATGGTTGTCGTCAGCTCGTGTCGTGAGATGTTGGGTTAAGTCCCGCAACGAGCGCAACCCTTGATCTTAGTTGCCATCATTAAGTTGGGCACTCTAAGGTGACTGCCGGTGACAAACCGGAGGAAGGTGGGGATGACGTCAAATCATCATGCCCCTTATGACCTGGGCTACACACGTGCTACAATGGACGGTACAAAGAGCTGCAAGACCGCGAGGTGGAGCTAATCTCATAAAACCGTTCTCAGTTCGGATTGTAGGCTGCAACTCGCCTACATGAAGCTGGAATCGCTAGTAATCGCGGATCAGCATGCCGCGGTGAATACGTTCCCGGGCCTTGTACACACCGCCCGTCACACCACGAGAGTTTGTAACACCCGAAGTCGGTGGGGTAACCTTTTTGGAGCCAGCCGCCTAAGGTGGGACAGATGATTGGGGTGAAGTCGTAACAAGGTAGCCGTATCGGAAGG | 481 | NR_115714.1 | 1044 - 1524 |
| 14 | Fibrobacteres | TCGTGTCGTGAGATGTTGGGTTAAGTCCCGCAACGAGCGCAACCCACGTTTCCAGTTGCCACCCGCAAGGGGGCCCTCTGGAGAGACTGCCGGGGACAACCCGGAGGAAGGTGTGGATGACGTCAAGTCCTCATGGCCCTTACATCCTGGGCTACACACGTGCTACAATGGTCGGTACAATGGGTCGCAACGCCGCGAGGCGGAGCCAATCCTCAAAGCCGTCCTCAGTTCGGATCGGAGTCTGCAACTCGACTCCGTGAAGCTGGAATCGCTAGTAATCGTGGGTCAGCACACCACGGTGAATACGTTCCCGGGCCTTGTACACACCGCCCGTCAAGCCATGGGAGAAGGGAGTGCTCTAAGTCGTGCAAGCGCCTAAAGCAAGACCTTTGACTGGGGCTAAGTCGTAACAAGGTAGCCGTACCGGAAGGTGCGGCTGGATTACCT | 447 | NR_074293.1 | 1041 - 1487 |
| 15 | Acidobacteria | GTTAAGTCCCGCAACGAGCGCAACCCTTATCTCCAGTTGCTACCATTTAGTTGAGCACTCTGGCGAAACCGCCTCGGATAACGGGGAGGAAGGTGGGGATGACGTCAAGTCCTCATGGCCTTTATGTCCAGGGCTACACACGTGCTACAATGGCCGGTACAAACCGCTGCAACCCCGCGAGGGTGAGCTAATCGGAAAAAGCCGGCCTCAGTTCGGATTGGAGTCTGCAACTCGACTCCATGAAGCTGGAATCGCTAGTAATCGTGGATCAGCATGCCACGGTGAATACGTTCCCGGGCCTTGTACACACCGCCCGTCACATCACGAAAGTGGGTTGCACTAGAAGTCGGTGCGCTAACCGCAAGGGAGCAGCCGCCCAAGGTGTAATTCATGATTGGGGTGAAGTCGTAACAAGGTAGCCGTAGGAGAACCTGCGGCTGGATCACCT | 448 | NR_074298.1 | 1041 - 1488 |
| 16 | Calditrichaeota | GCACAGGTGCTGCATGGCTGTCGTCAGCTCGTGTCGTGAGATGTTGGGTTAAGTCCCGCAACGAGCGCAACCCCTGCCTCTAGTTACCATCGGTTCAAGCCGGGGACTCTAGAGGGACTGCCGGCGATAAGCTGGAGGAAGGTGGGGATGACGTCAAGTCCTCATGGCCCTTACACCCCGGGCTACACACGTGCTACAATGGCCGGTACAGCGAGTTGCGAAACCGCGAGGTGGAGCCAATCTCTAAAAACCGGTCTCAGTTCGGATTGCAGTCTGCAACTCGACTGCATGAAGTCGGAATCGCTAGTAATCGCGGATCAGCATGCCGCGGTGAATACGTTCCCGGGCCTTGTACACACCGCCCGTCACGCCATGGAAGTCGGCAGTACCCGAAGCCCCCGCATTAGCGGGGTCGAAGGTAAGGCCGATGACTGGGGCGAAGTCGTAACAAGGTAGCCGTACCGGAAGGTGCGG | 474 | NR_028969.1 | 1031 - 1504 |
| 17 | Firmicutes (Calditerricola) | GGTGACAGGTGGTGCATGGTTGTCGTCAGCTCGTGTCGTGAGATGTTGGGTTAAGTCCCGCAACGAGCGCAACCCCTGCCCCTAGTTGCCAGCGGGTCATGCCGGGCACTCTAGGGGGACTGCCGGCGACAAGCCGGAGGAAGGTGGGGATGACGTCAAATCATCATGCCCCTTATGCCCTGGGCTACACACGTGCTACAATGGCCGGTACAAAGGGTTGCGAACCCGCGAGGGGGAGCCAATCCCAAAAAGCCGGTCTCAGTTCGGATTGCAGGCTGCAACTCGCCTGCATGAAGGCGGAATCGCTAGTAATCGCGGATCAGCATGCCGCGGTGAATACGTTCCCGGGCCTTGTACACACCGCCCGTCACACCACGAGAGTCTGCAACACCCGAAGTCGGTGCGCCAACCCCTCACGGGGAGGCAGCCGCCGAAGGTGGGGCAGATGATTGGGGTGAAGTCGTAACAAGGTA | 473 | NR_112684.1 | 1060 - 1532 |
| 18 | Chloroflexi | GCAACGAGCGCAACCCGTGTCGGTAGTTACAGGTGTCTACCGAGACTGCCGCCGTGACCGGCGGAGGAAGGCGCGGATGACGTCAAGTCAGCATGGCCCTTACGTCCGGGGCGACACACACGCTACAATGGCCACGACAATGCGTTGCCAAGCCGCAAGGTGGAGCTAATCGCCTAAACGTGGTCTCAGTGCAGATCGGGGGCTGCAACTCGCCCCCGTGAAGGCGGAGTTGCTAGTAACCGCGTATCAGCCATGGCGCGGTGAATACGTTCCCGGGCCTTGTACACACCGCCCGTCACGTCATGGGAGTGGCCAATGCTTGAAGTCCGTGTGCTAACCCCAGTCGGGGAGGCAGCGGCCGAGGGCAGGGGCCGCGACTGGGACGAAGTCGTAACAAGGTAGCCGTACCGGAAGGTGCGGCTGGATCACCTCCTTT | 436 | NR_074263.1 | 1044 - 1479 |
| 19 | Chrysiogenetes | CAGCTCGCGTCGTGAGATGTTGGGTTAAGTCCCGCAACGAGCGCAACCCCTGCCATTAGTTGCCATCATTAAGTTGGGCACTCTAGTGGGACAGCCGGAGTAATCCGGAGGAAGGTGGGGACGACGTCAAGTCATCATGGCCCTTATGACCAGGGCTACACACGTGCTACAATGGCAAGGACAACGGGATGCGACCTCGCGAGAGTGAGCCAACCTCAAAAACCTTGTCTTAGTTCGGATTGCAGTCTGCAACTCGACTGCATGAAGTCGGAATCGCTAGTAATCGCAGGTCAGCATACTGCGGTGAATACGTTCCCGGGCCTTGTACACACCGCCCGTCACACCACGAAAGTCGGTTTTGCCAGAAGCGGGTGACCGAATCTTCGGATAGGAGCCTTCGAAGGCAGGACTGGTGATTGGGGTGAAGTCGTAACAAGGTAGCCGTATCGGAAGGTGCGGCTGGATCACCTCCTT | 474 | NR_115927.2 | 1046 - 1519 |
| 20 | Bacteroidetes | TGGTTGTCGTCAGCTCGTGCCGTGAGGTGTCGGCTTAAGTGCCATAACGAGCGCAACCCTTATCTTTAGTTACTAACAGGTTATGCTGAGGACTCTAGAGAGACTGCCGTCGTAAGATGTGAGGAAGGTGGGGATGACGTCAAATCAGCACGGCCCTTACGTCCGGGGCTACACACGTGTTACAATGGGGGGTACAGAAGGCAGCTAGCGGGTGACCGTATGCTAATCCCAAAATCCTCTCTCAGTTCGGATCGAAGTCTGCAACCCGACTTCGTGAAGCTGGATTCGCTAGTAATCGCGCATCAGCCACGGCGCGGTGAATACGTTCCCGGGCCTTGTACACACCGCCCGTCAAGCCATGGGAGCCGGGGGTACCTGAAGTACGTAACCGCAAGGATCGTCCTAGGGTAAAACTGGTGACTGGGGCTAAGTCGTAACAAGGTAGCCGTACCGGAAGGTGCGGCTGGAACACCTCCTTT | 479 | NR_074784.2 | 1051 - 1529 |
| 21 | Aquificae | GTGTCGTGAGATGTTGGGTTAAGTCCCGCAACGAGCGCAACCCTTGCCCTGTGTTACCAGCGGGTAAAGCCGGGTACTCACAGGGGACTGCCGGCGATAAGTCGGAGGAAGGAGGGGATGACGTCAGATCAGTATGCCCTTTATGCCCTGGGCTACACAGGCGCTACAGTGGCAGGGACAATGGGACGCAACGCAGCAATGCGGAGCAAATCCCCTAAACCCTGTCGTGGTGCGGATTGGGGGTTGCAACTCACCCCCATGAAGGCGGAATCGGTAGTAATGGCGAATCAGCAATGTCGCCGTGAATACGTTCCCGGGTCTTGTACACACCGCCCGTCACGCCATGGGAGTCGGGTTCATCGGAAGTCCCCGAGCTAACCCGCAAGGGAGGCAGGGGCCGATGATGGGCCTGATGACTGGGGCGAAGTCGTAACAAGGTAGCCCTAGGGGAACCTGGGGCTGGATCACCT | 470 | NR_102858.1 | 1044 - 1513 |
| 22 | Caldiserica/Cryosericota group | ACACCCTATGGCACATGCACAGGTGCTGCATGGTTGTCGTCAGCTCGTGTCGTGAGATGTACGGTTAAGTCCGTGAACGAGCGCAACCCCTGCCCTTAGTTGCTAATAGGCTTCGGCCTATGCACTCTAAGGGGACTGCCAGCGATAAGCTGGAGGAAGGTGGGGATGACGTCAAATCCTCATGGCCCTTATGCCCAGGGCTACACACATGCGACAATGGTCGGGACAATGCGTTGCAAACCAGTAATGGGGAGCTAATCGCAAAAAACCGACCCCAGTACGGATTGAGGGTTGCAACTCACCCTCATGAAGCTGGAGTTGCTAGTAACCGCCGGTCAGCTATACGGCGATGAATACGTTCCCGGGTCTTGTACACACCGCCCGTCACACCACCCGAGTTGCGTGCACCCGAAGTGGCTCGGTGAGTCACGAAGGTGTGCGTGATGAGGAGGGTGAAGTCGTAACAAGGTAGGTGTACGAG | 481 | NR_075015.2 | 1048 - 1528 |
| 23 | Thermotogae | AGCCGGCACAGGTGGTGCACGGCCGTCGTCAGCTCGTGCCGTGAGGTGTTGGGTTAAGTCCCGCAACGAGCGCAACCCCTGCCCCTAGTTGCCAGCGGTTCGGCCGGGCACTCTAGGGGGACTGCCGGCGACGAGCCGGAGGAAGGAGGGGATGACGTCAGGTACTCGTGCCCCTTATGCCCTGGGCGACACACGCGCTACAATGGGCGGTACAATGGGTTGCGACCCCGCGAGGGGGAGCCAATCCCCAAAACCGCCCTCAGTTCGGATCGCAGGCTGCAACCCGCCTGCGTGAAGCCGGAATCGCTAGTAATCGCGGATCAGCCACGCCGCGGTGAATACGTTCCCGGGCCTTGTACACACCGCCCGTCACGCCACCCGAGTCGGGGGCTCCCGAAGACACCTGCCCCAACCCGAAAGGGAGGGGGGGTGTTGAGGGAGAACCTGGTGAGGGGGGCGAAGTCGTAACAAGGTAGCCGTA | 481 | NR_042374.2 | 1051 - 1531 |
| 24 | Tenericutes | TTGTCGTCAGCTCGTGTCGTGAGATGTTGGGTTAAGTCCCGCAACGAGCGCAACCCTTATCGTTAGTTACTTTGTCTAACGAGACTGCCAACGCAAGTTGGAGGAAGGTGGGGATGACGTCAAATCATCATGCCCCTTATGTCTAGGGCTGCAAACGTGCTACAATGGCCAATACAAACAGTTACCAAACCGTAAGGTGGAGTTAATCTGCAAAGTTGGTCTCAGTTCGGATTGAGGGCTGCAATTCGCCCTCATGAAGTCGGAATCACTAGTAATCGCGAATCAGCTATGTCGCGGTGAATACGTTCTCGGGTCTTGTACACACCGCCCGTCAAACTACGAGAGTTGATAGTGTCTAAAACCGTGTTGCTAACCGCAAGGAAGCGCATGTCTAGGACAAGATTAATGATTGGAGTTAAGTCGTAACAAGGTACCCCTACGAGAACGTGGGGGTGGATCACCTCCTTT | 468 | NR_117836.1 | 1036 - 1503 |
| 25 | Nitrospirae | GCCATCACACAGGTGCTGCATGGCTGTCGTCAGCTCGTGCCGTGAGGTGTTGGGTTCAGTCCCGCAACGAGCGCAACCCTCGCCCTTTGTTGCCATCGGGTAAAGCCGGGCACTCTAAGGGGACTGCCAGCGACAAGTTGGAGGAAGGAGAGGATGACGTCAAGTCATCATGGCCTTTATGCCTAGGGCCACACACGTGCAACAATGGCCGGTACAGACGGAGGCAATGCCGAGAGGCGGAGCAAACCCGAGAAAACCGGTCCCAGTTCGGATTGAGGTCTGCAACTCGACCTCATGAAGTCGGAATCGCTAGTAATCGCATATCAGAACGATGCGGTGAATACGTTCCCGGGCCTTGTACACACCGCCCGTCACACCACGAAAGTTTGTTGTACCCGAAGTCGGTGCCTTAACCTCGCAAGAGGAGAGAGCCGCCCAAGGTATGGCCGATGATTGGCGTG | 461 | NR_027216.1 | 1024 - 1484 |
| 26 | Lentisphaerae | GTGTTCGGTTAAGTCCGGCAACGAGCGCAACCCATATCCTTACTTGCTAACAGGTAATGCTGAGAACCTTAAGGAGACTGCCCGTGTTAAGCGGGAGGAAGGTGTGGACGACGTCAAGTCAGTATGGCCCTTACACCCGGGGCTGCACACCGTGCTACAATGGCCGGTACAAAGGGCAGCGACATAGTGATATGGAGCGAATCCCCAAAACCGGTCTCAGTACGGATTGGAGTCTGCAACTCGACTCCATGAAGATGGAATCGCTAGTAAATGGGCATCAGCTACGGCTCATTGAATACGTTCCCGGGCCTTGTACACACCGCCCGTCACATCATGGGAGCTGAGTTCACCCGAAGTCGTTGCGCCAACCTGCTTACAGGAGGCAGACGCCGAAGGTGGGCTTAGTGACTGGGATGAAGTCGTAACAAGGTAGCC | 435 | NR_027571.1 | 1044 - 1478 |
| 27 | Chlamydiae | AGGTGCTGCATGGCTGTCGTCAGCTCGTGCCGTGAGGTGTTGGGTTAAGTCCCGCAACGAGCGCAACCCTTATCGTTAGTTGCCAGCACTTAGGGTGGGAACTCTAACGAGACTGCCTGGGTTAACCAGGAGGAAGGCGAGGATGACGTCAAGTCAGCATGGCCCTTATGCCCAGGGCGACACACGTGCTACAATGGCCAGTACAGAAGGTAGCAAGATCGTGAGATGGAGCAAATCCTTAAAGCTGGCCCCAGTTCGGATTGTAGTCTGCAACTCGACTACATGAAGTCGGAATTGCTAGTAATGGCGTGTCAGCCATAACGCCGTGAATACGTTCCCGGGCCTTGTACACACCGCCCGTCACATCATGGGAGTTGGTTTTACCTTAAGTCGTTGACTCAACCCGCGAGGGGGAGAGGCGCCCAAGGTGAGGCTGATGACTAGGATGAAGTCGTAACAAGGTAGCCCTACCGGAAGGTGG | 481 | NR_029196.1 | 1046 - 1526 |
| 28 | Planctomycetes | GCCGTGAGGTGTTGGGTTAAGTCCCCTAACGAGCGAAACCCCTGTGTCTAGTTGCCAGCGGGTAAAGCCGGGAACTCTAGACAGACCGCCGGCGTTAAGCCGGAGGAAGGCGGGGATGACGTCAAGTCCTCATGGCCCTTATGCTTGGGGCTGCACACGTACTACAATGGGGCGGACAGAGCGTTGCTAGGCTGCAAAGTCATGCTAATCGCAAAAACCGTTCCTCAGTTCGGATTGCGGGCTGCAACCCGCCCGCATGAAGCTGGAATCGCTAGTAATCGCGGATCAGCATGCCGCGGTGAATGTGTTCCTGAGCCTTGTACACACCGCCCGTCAAGCCACCAAAGCGGGGGGCATCCGAAGTCGCCGGAGCCGCAAGGCAGGCGCCGAAGATGAAACCCGTGATGGGGACTAAGTCGTAACAAGGTAACCGTAGGGGAACCTGCGGTTGGATCACCTCCTTT | 464 | NR_102439.1 | 1044 - 1507 |
| 29 | Deferribacteres | GGGAGACAGGTGCTGCATGGCTGTCGTCAGCTCGTGCCGTGAGGTGTTGGGTTAAGTCCCGCAACGAGCGCAACCCCTACCCTTAGTTGCCATCGGTTAGGCCGGGCACTCTAAGGGGACTGCCCCGGATAACGGGGAGGAAGGTGGGGATGACGTCAAGTCATCATGGCCCTTATGTCCAGGGCTACACACGTGCTACAATGGGGCGTACAGAGGGCAGCGAAGCCGCGAGGCTGAGCGAATCTCAGAAAGCGCTCCTCAGTTCGGATCGCAGTCTGCAACTCGACTGCGTGAAGCCGGAATCGCTAGTAATCGCAGGTCAGCAAAACTGCGGTGAATACGTTCCCGGGCCTTGTACACACCGCCCGTCACACCACGGGAGTTGGCTATACCTGAAGCCGGTGGCCCAACCCAGGCAACTGGGGGGGAGCCGTCCATGGTATGGCTGGCGACTGGGGTGAAGTCGTAACAAGGTAGCCGT | 481 | NR_075025.2 | 1051 - 1531 |
| 30 | Cyanobacteria | GCAACCCACGTTTTTAGTTGCCAGCATTTAGTTGGGCACTCTAGAAAGACCGCCGGTGATAAACCGGAGGAAGGTGTGGATGACGTCAAGTCATCATGCCCCTTACACCCTGGGCTACACACGTACTACAATGCTACGGACAAAGGGCAGCAAACTCGCGAGAGCTAGCAAATCCCATAAACCGTGGCTCAGTTCAGATCGTAGGCTGCAACTCGCCTACGTGAAGTAGGAATCGCTAGTAATCGCAGGTCAGCATACTGCGGTGAATACGTTCCCGGGCCTTGTACACACCGCCCGTCACACCATGGAAGTTGGCCATGCCCGAAGTCGTTACTCCAACCCTTGTGGAGGAGGACGCCGAAGGTGGGGCTAATGACTGGGGTGAAGTCGTAACAAGGTAGCCGTACCGGAAGGTGCGGCTGGATCACCTCCTAA | 435 | NR_125480.1 | 1031 - 1465 |
| 31 | Fusobacteria | TGTCGTGAGATGTTGGGTTAAGTCCCGCAACGAGCGCAACCCCTTTCGTATGTTACCATCATTAAGTTGGGGACTCATGCGATACTGCCTACGATGAGTAGGAGGAAGGTGGGGATGACGTCAAGTCATCATGCCCCTTATACGCTGGGCTACACACGTGCTACAATGGGTAGAACAGAGAGTCGCAAAGCTGTGAAGTGGAGCTAATCTCAGAAAACTATTCTTAGTTCGGATTGTACTCTGCAACTCGAGTACATGAAGTTGGAATCGCTAGTAATCGCGAATCAGCAATGTCGCGGTGAATACGTTCTCGGGTCTTGTACACACCGCCCGTCACACCACGAGAGTTGGTTGCACAAGAAGTAGCAGGCCTAACCGTAAGGAGGGATGCTCCGAGGGTGTGATTAGCGATTGGGGTGAAGTCGTAACAAGGTATCCGTACGGGAACGTGCGGCTGGATC | 461 | NR_115068.1 | 1035 - 1495 |

Table 2 Oligomers for OE-PCR synthesis of templates

| Template No | Oligomer Name | Oligomer sequence | Oligomer length |
| --- | --- | --- | --- |
| 1 | Actinobacteria_oe1 | CGTGTCGTGAGATGTTGGGTTAAGTCCCGCAACGAGCGCAACCCTTGTCTTATGTTGCCAGCA | 63 |
|  | Actinobacteria_oe2 | CCTCCGAGTTAACCCCGGCAGTCTCTCATGAGTCCCCACCATCACGTGCTGGCAACATAAGAC | 63 |
|  | Actinobacteria_oe3 | GGGGTTAACTCGGAGGAAGGTGGGGATGACGTCAAATCATCATGCCCCTTATGTCCAGGGCT | 62 |
|  | Actinobacteria_oe4 | TCACAGGCTCGCAGCGCGTTGTACCGACCATTGTAGCATGTGTGAAGCCCTGGACATAAGGG | 62 |
|  | Actinobacteria_oe5 | GCTGCGAGCCTGTGAGGGTGAGCGAATCGCTGAAAGCCGGCCTCAGTTCGGATTGGGGTCT | 61 |
|  | Actinobacteria_oe6 | TGCTGATCTGCGATTACTAGCGACTCCGACTTCATGGGGTCGAGTTGCAGACCCCAATCCGA | 62 |
|  | Actinobacteria_oe7 | AGTAATCGCAGATCAGCAACGCTGCGGTGAATACGTTCCCGGGCCTTGTACACACCGCCCG | 61 |
|  | Actinobacteria_oe8 | CAAGGGTTAGGCCACTGGCTTCGGGTGTTACCAACTTTCATGACGTGACGGGCGGTGTGTA | 61 |
|  | Actinobacteria_oe9 | CAGTGGCCTAACCCTTGTGGGGGGAGCTGTCGAAGGTGGGATCGGCGATTGGGACGAAGTCGTA | 64 |
|  | Actinobacteria_oe10 | AAAGGAGGTGATCCAGCCGCACCTTCCGGTACGGCTACCTTGTTACGACTTCGTCCCAATC | 61 |
| 2 | Spirochaetes_oe1 | TTGGGTTAAGTCCCGCAACGAGCGCAACCCTCACCTTATGTTGCCATCATTCAGTTGGGCAC | 62 |
|  | Spirochaetes_oe2 | TCCCCGCCTTCCTCCGGTTTGTCACCGGCAGTTCCTTACGAGTGCCCAACTGAATGATG | 59 |
|  | Spirochaetes_oe3 | AGGAAGGCGGGGATGACGTCAAATCCTCATGGCCTTTATGTCTAGGGCAACACACGTG | 58 |
|  | Spirochaetes_oe4 | CTCCCCCTCGCGAGTTGGCTACCCTTTGTACCGGCCATTGTAGCACGTGTGTTGCCC | 57 |
|  | Spirochaetes_oe5 | CGCGAGGGGGAGCTAATCTCAAAAATCCGGTCCCAGTTCGGATTGGAGTCTGCAACTC | 58 |
|  | Spirochaetes_oe6 | ATGCTGATCCGCGATTACTAGCGATTCCGACTTCATGGAGTCGAGTTGCAGACTCCAA | 58 |
|  | Spirochaetes_oe7 | CGCGGATCAGCATGCCGCGGTGAATACGTTCCCGGACCTTGTACACACCGCCCGTCA | 57 |
|  | Spirochaetes_oe8 | GCGGTTGGCAAAGACCACTTCGGGTGCTCCCCACTCAGGTGGTGTGACGGGCGGTG | 56 |
|  | Spirochaetes_oe9 | CTTTGCCAACCGCAAGGAAGCAGACTACTAAGGTGAAACTCGTGAAGGGGGTGAAGTCG | 59 |
|  | Spirochaetes_oe10 | AAAGGAGGTGATCCAGCCGCACCTTCCGATACGGCTACCTTGTTACGACTTCACCCCCTTC | 61 |
| 3 | Synergistetes_oe1 | GTCGTGAGATGTTGGGTTAAGTCCCGCAACGAGCGCAACCCCTATTGCCAGTTGCTAACGGGA | 63 |
|  | Synergistetes_oe2 | CTTCCTCCGCCTTGTCGACGGCAGTCTCGCCAGAGTCCTCGGCTCTCCCGTTAGCAACTGG | 61 |
|  | Synergistetes_oe3 | CAAGGCGGAGGAAGGTGGGGACGACGTCAAGTCATCATGGCCTTTATGCCCAGGGCGAC | 59 |
|  | Synergistetes_oe4 | ACCTCGCGGTCTCGCTCCCCTCTGTGCCGGCCATTGTAGCACGTGTGTCGCCCTGGGC | 58 |
|  | Synergistetes_oe5 | GAGACCGCGAGGTGGAGCGAATCCCAGAAAGCCGGTCCCAGTTCGGATTGCAGTCTGCAACT | 62 |
|  | Synergistetes_oe6 | TTGGCTGATCCGCGATTACTAGCGATTCCGGCTTCATGCAGTCGAGTTGCAGACTGCAATCC | 62 |
|  | Synergistetes_oe7 | CGCGGATCAGCCAAGCCGCGGTGAATACGTTCCCGGGCCTTGTACACACCGCCCGTCAC | 59 |
|  | Synergistetes_oe8 | CCTCGCAGGTTAGGCCGGTGACTTCGGGTACACCCAACTCGGGTGGTGTGACGGGCGGT | 59 |
|  | Synergistetes_oe9 | GCCTAACCTGCGAGGGGAGGAGGTGCCTAAGGTATGCTTGGTAAGGGGGGTGAAGTCGTAAC | 62 |
|  | Synergistetes_oe10 | AAAGGAGGTGATCCAGCCGCACCTTCCGGTACGGCTACCTTGTTACGACTTCACCCCC | 58 |
| 4 | Dictyoglomi_oe1 | TGGAGCCTGCACAGGTGGTGCATGGCTGTCGTCAGCTCGTGTCGTGAGATGTTGGGTTAAGTCC | 64 |
|  | Dictyoglomi_oe2 | GGCTTTACCCGCTGGCAACTAAGGGCAGGGGTTGCGCTCGTTGCGGGACTTAACCCAACATC | 62 |
|  | Dictyoglomi_oe3 | AGCGGGTAAAGCCGGGCACTCTAAGGGGACTGCCGGCGAAGAGCCGGAGGAAGGTGGGGAT | 61 |
|  | Dictyoglomi_oe4 | GTAGCGCGTGTGTAGCCCAGGGCATAAGGGGCATACTGACTTGACGTCATCCCCACCTTCCTC | 63 |
|  | Dictyoglomi_oe5 | GCTACACACGCGCTACAATGGGTGGTACAGAGGGGAGCGAAGCCGCGAGGCGGAGCGAATC | 61 |
|  | Dictyoglomi_oe6 | ACGCAGGCGAGTTGCAGCCTGCGATCTGAACTGGGGGTGGCTTTAGGGATTCGCTCCGCC | 60 |
|  | Dictyoglomi_oe7 | AACTCGCCTGCGTGAAGGCGGAATCGCTAGTAACCGCAGATCAGCCACGCTGCGGTGAATA | 61 |
|  | Dictyoglomi_oe8 | GCGGACTCTCGTGGTGTGACGGGCGGTGTGTACAAGGCCCGAGAACGTATTCACCGCAGCGT | 62 |
|  | Dictyoglomi_oe9 | CCACGAGAGTCCGCAACACCCGAAGTCAGGCGAAGAGCCTGCCGAAGGTGGGGCGGATGA | 60 |
|  | Dictyoglomi_oe10 | CCGCACCTTCCGGTACGGCTACCTTGTTACGACTTCACCCCAATCATCCGCCCCAC | 56 |
| 5 | Thermodesulfobacteria_oe1 | TGCCTGGGAGCCCTAGCACAGGTGCTGCATGGCTGTCGTCAGCTCGTGTCGTGAGATGTTGGGTT | 65 |
|  | Thermodesulfobacteria_oe2 | ACCCGCTGGCAACTAAGGGCAAGGGTTGCGCTCGTTGCGGGACTTAACCCAACATCTCACGAC | 63 |
|  | Thermodesulfobacteria_oe3 | AGTTGCCAGCGGGTAGAGCCGGGCACTCTAGGGGGACTGCCGGGGACAACCCGGAGGAAG | 60 |
|  | Thermodesulfobacteria_oe4 | TGTGTAGCCCTGGGCATAAGGGCCATGATGACTTGACGTCATCCCCCCCTTCCTCCGGGTT | 61 |
|  | Thermodesulfobacteria_oe5 | GCCCAGGGCTACACACGTGCTACAATGGGCGGTACAGAGGGAAGCGAACCCGTAAGGGGGAG | 62 |
|  | Thermodesulfobacteria_oe6 | TCGAGTTGCAGACCCCGATCCGTACTGAGAGCGGCTTTCTGGGATTTGCTCCCCCTTACGGG | 62 |
|  | Thermodesulfobacteria_oe7 | GGGGTCTGCAACTCGACCCCGTGAAGCCGGAATCGCTAGTAACGGCGGATCAGCATGCCGC | 61 |
|  | Thermodesulfobacteria_oe8 | CCCGTGGTGTGACGGGCGGTGTGTACAAGGCCCGGGAACGTATTCACGGCGGCATGCTGA | 60 |
|  | Thermodesulfobacteria_oe9 | CGTCACACCACGGGAGCTGGCTCTGCCCGAAGTCGCTATCCCAACCCCCGGAAGGGGGAGG | 61 |
|  | Thermodesulfobacteria_oe10 | TTACGACTTCACCCCAGTCACCAGCCCTGCCGTCGGCGCCTCCCTCCCCCTTCCG | 55 |
| 6 | Elusimicrobia_oe1 | GTCGTCAGCTCGTGTCGTGAGATGTTGGGTTAAGTCCCGCAACGAGCGCAACCCCTATTCTG | 62 |
|  | Elusimicrobia_oe2 | TTATCCGCGGCAGTCTTCTGAGAGATCCTATTGCTAGGCAACACAGAATAGGGGTTGCG | 59 |
|  | Elusimicrobia_oe3 | CTGCCGCGGATAACGTGGAGGAAGGTGGGGATGACGTCAAATCATCATGGCCTTTATGTC | 60 |
|  | Elusimicrobia_oe4 | TATTGCTGCCCTCTGTCTATGCCATTGTATTACGTGTGTAGCCCTGGACATAAAGGCCATGAT | 63 |
|  | Elusimicrobia_oe5 | AGACAGAGGGCAGCAATATCGCGAGATGGAGCCAATCCCTAAACTATGCCCCAGTTCAGATT | 62 |
|  | Elusimicrobia_oe6 | TGCGATTACTAGAGATTCCGGCTTCATGCAGGCGAATTGCAGCCTGCAATCTGAACTGGGGCA | 63 |
|  | Elusimicrobia_oe7 | CGGAATCTCTAGTAATCGCAGATCAGCACGCTGCGGTGAATACGTTCCCGGGTCTTGTACACA | 63 |
|  | Elusimicrobia_oe8 | ACCTGAGCACTTCTGTTGCAATTAACTTTCGTGGTGTGACGGGCGGTGTGTACAAGACCCGGG | 63 |
|  | Elusimicrobia_oe9 | CAACAGAAGTGCTCAGGTCGTCTGGGCCCTAAGTTGTGATTAGTAATTGGGGTGAAGTCGTA | 62 |
|  | Elusimicrobia_oe10 | AAAGGAGGTGATCCAGCCGCACCTTCCGGTACGGCTACCTTGTTACGACTTCACCCCAAT | 60 |
| 7 | Chlorobi_oe1 | GTGTCGTGAGATGTTGGGTTAAGTCCCGCAACGAGCGCAACCCCTACAATTAGTTACTAACAGGT | 65 |
|  | Chlorobi_oe2 | TCACTACTTGCGTAGGCAGTTCAATTAGAGTCCTCAGCTTAACCTGTTAGTAACTAATTGTAGGG | 65 |
|  | Chlorobi_oe3 | TGCCTACGCAAGTAGTGAGGAAGGAGGGGATGACGTCAAGTCCTCATGGCCCTTACGCCT | 60 |
|  | Chlorobi_oe4 | GCTTTGCCCTCTGTAGCTGCCATTGTATCACGTGTGTGGCCCTAGGCGTAAGGGCCA | 57 |
|  | Chlorobi_oe5 | ACAGAGGGCAAAGCCGCGAGGTGGAGGAAATCCCTTAAAAGCTGTCTCAGTCCGGATCG | 59 |
|  | Chlorobi_oe6 | TGCGATTACTAGCGATTCCAACTTCACGGAGTCGAGTTGCAGACTCCGATCCGGACTGAG | 60 |
|  | Chlorobi_oe7 | GGAATCGCTAGTAATCGCAGATCAGCATGCTGCGGTGAATGTGTTCCCGGGCCTTGTACAC | 61 |
|  | Chlorobi_oe8 | GAGCGTCTTTGGGTACTCCTGACTTCCATGACTTGACGGGCGGTGTGTACAAGGCCCGG | 59 |
|  | Chlorobi_oe9 | ACCCAAAGACGCTCGCGCGTTTAAGGTAAGACTGGTAACTGGGACTAAGTCGTAACAAGGTA | 62 |
|  | Chlorobi_oe10 | AAAGGAGGTGATCCAGCCGCACCTTCCGGTACGGCTACCTTGTTACGACTTAGTCC | 56 |
| 8 | Verrucomicrobia_oe1 | AGATGTTTGGTTAAGTCCAGCAACGAGCGCAACCCCTGTTGCCAGTTACCAGCACGTGAAGG | 62 |
|  | Verrucomicrobia_oe2 | CACCTTCCTCCCAGTTGATCTGGGCAGTCTCGCCAGAGTCCCCACCTTCACGTGCTGGTAA | 61 |
|  | Verrucomicrobia_oe3 | ACTGGGAGGAAGGTGGGGACGACGTCAGGTCAGTATGGCCCTTATGCCCAGGGCTGCACA | 60 |
|  | Verrucomicrobia_oe4 | GCCTCGCGGCTTCGGCCCCCTCTGTACTGGGCATTGTAGTACGTGTGCAGCCCTGGG | 57 |
|  | Verrucomicrobia_oe5 | GCCGCGAGGCGGAGGAAATCCTAAAAACTGGGCCCAGTTCGGACTGTAGGCTGCAACC | 58 |
|  | Verrucomicrobia_oe6 | GTAGCTGATGCGCCATTACTAGCGATTCCGGCTTCGTGTAGGCGGGTTGCAGCCTACAGT | 60 |
|  | Verrucomicrobia_oe7 | GGCGCATCAGCTACGGCGCCGTGAATACGTTCCCGGGTCTTGTACACACCGCCCGTCA | 58 |
|  | Verrucomicrobia_oe8 | TTGCGGTTGGCTTCAGATACTTCGGGTGCGACCGGCTTCCATGATGTGACGGGCGGTG | 58 |
|  | Verrucomicrobia_oe9 | GAAGCCAACCGCAAGGAGGCAGGGTCCTAAGGTGAGACTGGTAACTGGGATGAAGTCGTAA | 61 |
|  | Verrucomicrobia_oe10 | AAAGGAGGTGATCCAGCCGCAGGTTCCCCTACGGCTACCTTGTTACGACTTCATCCCAGTT | 61 |
| 9 | Deinococcus-Thermus_oe1 | CTCGTGCCGTGAGGTGTTGGGTTAAGTCCCGCAACGAGCGCAACCCCCGCCGTTAGTTGCCA | 62 |
|  | Deinococcus-Thermus_oe2 | TCCCGCTTTCGCGGGCAGTCCCGTTAGAGTGCCCGGCCGAACCGCTGGCAACTAACGGCG | 60 |
|  | Deinococcus-Thermus_oe3 | CGCGAAAGCGGGAGGAAGGAGGGGACGACGTCTGGTCAGCATGGCCCTTACGGCCTGGG | 59 |
|  | Deinococcus-Thermus_oe4 | GCCGGGTGGCATCGCTTTGTAGTGGGCATTGTAGCACGTGTGTCGCCCAGGCCGTAAG | 58 |
|  | Deinococcus-Thermus_oe5 | TGCCACCCGGCAACGGGGAGCTAATCGCAAAAAGGTGGGCCCAGTTCGGATTGGGGTCT | 59 |
|  | Deinococcus-Thermus_oe6 | CTGATCCGCGATTACTAGCGATTCCGGCTTCATGGGGTCGGGTTGCAGACCCCAATCCGA | 60 |
|  | Deinococcus-Thermus_oe7 | GTAATCGCGGATCAGCCATGCCGCGGTGAATACGTTCCCGGGCCTTGTACACACCGCCCG | 60 |
|  | Deinococcus-Thermus_oe8 | CGTAGGCTCCCGGCGACTTCGGGTAGAGCCCGCTCCCATGGCGTGACGGGCGGTGTGTA | 59 |
|  | Deinococcus-Thermus_oe9 | CCGGGAGCCTACGGGCAGGCGCCGAGGGTAGGGCCCGTGACTGGGGCGAAGTCGTAACAAGG | 62 |
|  | Deinococcus-Thermus_oe10 | AAAGGAGGTGATCCAGCCGCACCTTCCGGTACAGCTACCTTGTTACGACTTCGCC | 55 |
| 10 | Ignavibacteriae_oe1 | TGGCTGTCGTCAGCTCGTGCCGTGAGGTGTTGGGTTAAGTCCCGCAACGAGCGCAACCCCTA | 62 |
|  | Ignavibacteriae_oe2 | GTAGGCAGTCCCATTAGAGTGCCCAGCTTAACCTGATGGCAACTAATGGTAGGGGTTGCGCT | 62 |
|  | Ignavibacteriae_oe3 | CTAATGGGACTGCCTACGCAAGTAGTGAGGAAGGTGGGGATGACGTCAAGTCAGCATGGCCCT | 63 |
|  | Ignavibacteriae_oe4 | GCTACCCGTTGTAGCACCCATTGTAGCACGTGTGTAGCCCTAGGCGTAAGGGCCATGCTGACT | 63 |
|  | Ignavibacteriae_oe5 | TGCTACAACGGGTAGCGAAACCGCGAGGTGGAGCCAATCCCTAAAAAGCATCCTCAGTTCGGA | 63 |
|  | Ignavibacteriae_oe6 | GCGATTACTAGCAATTCCAGCTTCATGGAGTCGGGTTGCAGACTCCAATCCGAACTGAGGATGC | 64 |
|  | Ignavibacteriae_oe7 | GGAATTGCTAGTAATCGCGCATCAGCACGGCGCGGTGAATACGTTCCCGGGCCTTGTACACAC | 63 |
|  | Ignavibacteriae_oe8 | GTTGGGTCACTGACTTCGGGTACCCCCGGCTTCCATGGCTTGACGGGCGGTGTGTACAAGGCCCG | 65 |
|  | Ignavibacteriae_oe9 | CGAAGTCAGTGACCCAACTCCGCCTCGGCGGAGAGGGAGCTGCCGAAGGTAAAACCGGTGACTG | 64 |
|  | Ignavibacteriae_oe10 | GCCGCACCTTCCGGTACGGCTACCTTGTTACGACTTAGCCCCAGTCACCGGTTTTACC | 58 |
| 11 | Gemmatimonadetes_oe1 | GTGTCGTGAGATGTTGGGTTAAGTCCCGCAACGAGCGCAACCCTTGCCCTTAGTTACCAGCGAG | 64 |
|  | Gemmatimonadetes_oe2 | CTCCGGTTTGGCACCGGCAGTCCCCCTAGAGTCCCCGACTTTACTCGCTGGTAACTAAGGG | 61 |
|  | Gemmatimonadetes_oe3 | TGCCAAACCGGAGGAAGGTGGGGACGACGTCAAGTCATCATGGTCCTTACGTCTGGGGCTA | 61 |
|  | Gemmatimonadetes_oe4 | GATTGCTCTTTCGCAGCCCTCTGTACCGGCCATTGTAGCACGTGTGTAGCCCCAGACGTAAGG | 63 |
|  | Gemmatimonadetes_oe5 | GCTGCGAAAGAGCAATCTGGAGCCAATCCCTAAAGCCGGCCTCAGTTCGGATTGTCGTCTGCA | 63 |
|  | Gemmatimonadetes_oe6 | GCTGATCCGCGATTACTAGCGATTCCAGCTTCATGCCGTCGAGTTGCAGACGACAATCCG | 60 |
|  | Gemmatimonadetes_oe7 | TCGCGGATCAGCGACGCCGCGGTGAATACGTTCCCGGGCCTTGTACACACCGCCCGTC | 58 |
|  | Gemmatimonadetes_oe8 | TTGCGGGTTCCTGCACCGACTTCGGGCGCTCACAGCTTCCATGGCGTGACGGGCGGTGT | 59 |
|  | Gemmatimonadetes_oe9 | GCAGGAACCCGCAAGGGGCCAAGCCGCCTAAGGCGAGCGCAGTGACTGGGGCGAAGTCGT | 60 |
|  | Gemmatimonadetes_oe10 | AAAGGAGGTGATCCAGCCGCAGGTTCCCCTACGGCTACCTTGTTACGACTTCGCCCCA | 58 |
| 12 | Proteobacteria_oe1 | CTGCATGGCTGTCGTCAGCTCGTGTTGTGAAATGTTGGGTTAAGTCCCGCAACGAGCGCAAC | 62 |
|  | Proteobacteria_oe2 | GCAGTCTCCTTTGAGTTCCCGGCCTAACCGCTGGCAACAAAGGATAAGGGTTGCGCTCGTTG | 62 |
|  | Proteobacteria_oe3 | AACTCAAAGGAGACTGCCAGTGATAAACTGGAGGAAGGTGGGGATGACGTCAAGTCATCATGGC | 64 |
|  | Proteobacteria_oe4 | TTCTCTTTGTATATGCCATTGTAGCACGTGTGTAGCCCTGGTCGTAAGGGCCATGATGACTTGACG | 66 |
|  | Proteobacteria_oe5 | AATGGCATATACAAAGAGAAGCGACCTCGCGAGAGCAAGCGGACCTCATAAAGTATGTCGTAGTCCG | 67 |
|  | Proteobacteria_oe6 | ATTACTAGCGATTCCGACTTCATGGAGTCGAGTTGCAGACTCCAATCCGGACTACGACATACTTTAT | 67 |
|  | Proteobacteria_oe7 | AAGTCGGAATCGCTAGTAATCGTGGATCAGAATGCCACGGTGAATACGTTCCCGGGCCTTGTAC | 64 |
|  | Proteobacteria_oe8 | TAAGCTACCTACTTCTTTTGCAACCCACTCCCATGGTGTGACGGGCGGTGTGTACAAGGCCCGGG | 65 |
|  | Proteobacteria_oe9 | GCAAAAGAAGTAGGTAGCTTAACCTTCGGGAGGGCGCTTACCACTTTGTGATTCATGACTGGGGT | 65 |
|  | Proteobacteria_oe10 | TCCAACCGCAGGTTCCCCTACGGTTACCTTGTTACGACTTCACCCCAGTCATGAATCA | 58 |
| 13 | Firmicutes_oe1 | GTGACAGGTGGTGCATGGTTGTCGTCAGCTCGTGTCGTGAGATGTTGGGTTAAGTCCCGCAAC | 63 |
|  | Firmicutes_oe2 | CCTTAGAGTGCCCAACTTAATGATGGCAACTAAGATCAAGGGTTGCGCTCGTTGCGGGACTTAAC | 65 |
|  | Firmicutes_oe3 | TTAAGTTGGGCACTCTAAGGTGACTGCCGGTGACAAACCGGAGGAAGGTGGGGATGACGTCAAAT | 65 |
|  | Firmicutes_oe4 | ACCGTCCATTGTAGCACGTGTGTAGCCCAGGTCATAAGGGGCATGATGATTTGACGTCATCCCC | 64 |
|  | Firmicutes_oe5 | CGTGCTACAATGGACGGTACAAAGAGCTGCAAGACCGCGAGGTGGAGCTAATCTCATAAAACCGTT | 66 |
|  | Firmicutes_oe6 | ATTCCAGCTTCATGTAGGCGAGTTGCAGCCTACAATCCGAACTGAGAACGGTTTTATGAGATTAGC | 66 |
|  | Firmicutes_oe7 | GCCTACATGAAGCTGGAATCGCTAGTAATCGCGGATCAGCATGCCGCGGTGAATACGTTCCCGG | 64 |
|  | Firmicutes_oe8 | CTTCGGGTGTTACAAACTCTCGTGGTGTGACGGGCGGTGTGTACAAGGCCCGGGAACGTATTCA | 64 |
|  | Firmicutes_oe9 | GAGTTTGTAACACCCGAAGTCGGTGGGGTAACCTTTTTGGAGCCAGCCGCCTAAGGTGGGACAGAT | 66 |
|  | Firmicutes_oe10 | CCTTCCGATACGGCTACCTTGTTACGACTTCACCCCAATCATCTGTCCCACCTTAGGC | 58 |
| 14 | Fibrobacteres_oe1 | TCGTGTCGTGAGATGTTGGGTTAAGTCCCGCAACGAGCGCAACCCACGTTTCCAGTTGCC | 60 |
|  | Fibrobacteres_oe2 | CGGGTTGTCCCCGGCAGTCTCTCCAGAGGGCCCCCTTGCGGGTGGCAACTGGAAACGT | 58 |
|  | Fibrobacteres_oe3 | GGGGACAACCCGGAGGAAGGTGTGGATGACGTCAAGTCCTCATGGCCCTTACATCCT | 57 |
|  | Fibrobacteres_oe4 | GTTGCGACCCATTGTACCGACCATTGTAGCACGTGTGTAGCCCAGGATGTAAGGGCCA | 58 |
|  | Fibrobacteres_oe5 | AATGGGTCGCAACGCCGCGAGGCGGAGCCAATCCTCAAAGCCGTCCTCAGTTCGGATC | 58 |
|  | Fibrobacteres_oe6 | ACGATTACTAGCGATTCCAGCTTCACGGAGTCGAGTTGCAGACTCCGATCCGAACTGAGGA | 61 |
|  | Fibrobacteres_oe7 | TGGAATCGCTAGTAATCGTGGGTCAGCACACCACGGTGAATACGTTCCCGGGCCTTGTACA | 61 |
|  | Fibrobacteres_oe8 | TGCACGACTTAGAGCACTCCCTTCTCCCATGGCTTGACGGGCGGTGTGTACAAGGCCCGG | 60 |
|  | Fibrobacteres_oe9 | AGTGCTCTAAGTCGTGCAAGCGCCTAAAGCAAGACCTTTGACTGGGGCTAAGTCGTAACAAG | 62 |
|  | Fibrobacteres_oe10 | AAAGGAGGTAATCCAGCCGCACCTTCCGGTACGGCTACCTTGTTACGACTTAGCCC | 56 |
| 15 | Acidobacteria_oe1 | GTTAAGTCCCGCAACGAGCGCAACCCTTATCTCCAGTTGCTACCATTTAGTTGAGCACTCTG | 62 |
|  | Acidobacteria_oe2 | CGTCATCCCCACCTTCCTCCCCGTTATCCGAGGCGGTTTCGCCAGAGTGCTCAACTAAATG | 61 |
|  | Acidobacteria_oe3 | AAGGTGGGGATGACGTCAAGTCCTCATGGCCTTTATGTCCAGGGCTACACACGTGCTACAA | 61 |
|  | Acidobacteria_oe4 | GATTAGCTCACCCTCGCGGGGTTGCAGCGGTTTGTACCGGCCATTGTAGCACGTGTGTAGCC | 62 |
|  | Acidobacteria_oe5 | CGAGGGTGAGCTAATCGGAAAAAGCCGGCCTCAGTTCGGATTGGAGTCTGCAACTCGACTC | 61 |
|  | Acidobacteria_oe6 | GTGGCATGCTGATCCACGATTACTAGCGATTCCAGCTTCATGGAGTCGAGTTGCAGACTC | 60 |
|  | Acidobacteria_oe7 | GGATCAGCATGCCACGGTGAATACGTTCCCGGGCCTTGTACACACCGCCCGTCACATCA | 59 |
|  | Acidobacteria_oe8 | TCCCTTGCGGTTAGCGCACCGACTTCTAGTGCAACCCACTTTCGTGATGTGACGGGCG | 58 |
|  | Acidobacteria_oe9 | GCTAACCGCAAGGGAGCAGCCGCCCAAGGTGTAATTCATGATTGGGGTGAAGTCGTAACAA | 61 |
|  | Acidobacteria_oe10 | AAAGGAGGTGATCCAGCCGCAGGTTCTCCTACGGCTACCTTGTTACGACTTCACCCC | 57 |
| 16 | Calditrichaeota_oe1 | GCACAGGTGCTGCATGGCTGTCGTCAGCTCGTGTCGTGAGATGTTGGGTTAAGTCCCGCAACG | 63 |
|  | Calditrichaeota_oe2 | TAGAGTCCCCGGCTTGAACCGATGGTAACTAGAGGCAGGGGTTGCGCTCGTTGCGGGACTTAA | 63 |
|  | Calditrichaeota_oe3 | CAAGCCGGGGACTCTAGAGGGACTGCCGGCGATAAGCTGGAGGAAGGTGGGGATGACGTCAAG | 63 |
|  | Calditrichaeota_oe4 | GGCCATTGTAGCACGTGTGTAGCCCGGGGTGTAAGGGCCATGAGGACTTGACGTCATCCCCA | 62 |
|  | Calditrichaeota_oe5 | CGTGCTACAATGGCCGGTACAGCGAGTTGCGAAACCGCGAGGTGGAGCCAATCTCTAAAAACCG | 64 |
|  | Calditrichaeota_oe6 | TCCGACTTCATGCAGTCGAGTTGCAGACTGCAATCCGAACTGAGACCGGTTTTTAGAGATTGGC | 64 |
|  | Calditrichaeota_oe7 | GACTGCATGAAGTCGGAATCGCTAGTAATCGCGGATCAGCATGCCGCGGTGAATACGTTCCCG | 63 |
|  | Calditrichaeota_oe8 | CGGGTACTGCCGACTTCCATGGCGTGACGGGCGGTGTGTACAAGGCCCGGGAACGTATTCAC | 62 |
|  | Calditrichaeota_oe9 | GTCGGCAGTACCCGAAGCCCCCGCATTAGCGGGGTCGAAGGTAAGGCCGATGACTGGGGCG | 61 |
|  | Calditrichaeota_oe10 | GATCCAGCCGCACCTTCCGGTACGGCTACCTTGTTACGACTTCGCCCCAGTCATCG | 56 |
| 17 | candidateNC10_oe1 | AGCGTGGAGACAGGTGGTGCATGGCTGTCGTCAGCTCGTGTCGTGAGATGTTGGGTTAAGTCCC | 64 |
|  | candidateNC10_oe2 | TCGGCTTTATCCGGTGGCAACCAGGGGTGAGGGTTGCGCTCGTTGCGGGACTTAACCCAACA | 62 |
|  | candidateNC10_oe3 | ACCGGATAAAGCCGAGCACTCGAGGGGAACTGCCAGCGTCAAGCTGGAGGAAGGCGGGGAT | 61 |
|  | candidateNC10_oe4 | TTGTAGCACGTGTGTAGCCCAGGGCGTAAGGGGCATGATGACTTGACGTCATCCCCGCCTTCC | 63 |
|  | candidateNC10_oe5 | GGCTACACACGTGCTACAATGGCCGGTACAAAGGGACGCGAGACCGCGAGGTGGAGCTAATCCCA | 65 |
|  | candidateNC10_oe6 | TTCATGCAGTCGAGTTGCAGACTGCAATCTGAACTTAGACCGGTTTTTTGGGATTAGCTCCACC | 64 |
|  | candidateNC10_oe7 | GCAACTCGACTGCATGAAGGAGGAATCGCTAGTAATCGCGCATCAGCACGGCGCGGTGAATA | 62 |
|  | candidateNC10_oe8 | ACAGACAGCTTTCGTGGTGTGACGGGCGGTGTGTACAAGGCCCGGGAACGTATTCACCGCGCCG | 64 |
|  | candidateNC10_oe9 | ACCACGAAAGCTGTCTGTACTCGAAGTCGCTGAGCTAACCCGCAAGGGGGGCAGGCGCCGAA | 62 |
|  | candidateNC10_oe10 | ACGGCTACCTTGTTACGACTTCACCCCAATCACTGACCATACCTTCGGCGCCTGC | 55 |
| 18 | Chloroflexi_oe1 | GCAACGAGCGCAACCCGTGTCGGTAGTTACAGGTGTCTACCGAGACTGCCGCCGTGA | 57 |
|  | Chloroflexi_oe2 | AGGGCCATGCTGACTTGACGTCATCCGCGCCTTCCTCCGCCGGTCACGGCGGCAG | 55 |
|  | Chloroflexi_oe3 | AGTCAGCATGGCCCTTACGTCCGGGGCGACACACACGCTACAATGGCCACGACAATG | 57 |
|  | Chloroflexi_oe4 | GACCACGTTTAGGCGATTAGCTCCACCTTGCGGCTTGGCAACGCATTGTCGTGGCCA | 57 |
|  | Chloroflexi_oe5 | TCGCCTAAACGTGGTCTCAGTGCAGATCGGGGGCTGCAACTCGCCCCCGTGAAGGC | 56 |
|  | Chloroflexi_oe6 | TATTCACCGCGCCATGGCTGATACGCGGTTACTAGCAACTCCGCCTTCACGGGG | 54 |
|  | Chloroflexi_oe7 | TGGCGCGGTGAATACGTTCCCGGGCCTTGTACACACCGCCCGTCACGTCATGGGAGT | 57 |
|  | Chloroflexi_oe8 | CTCCCCGACTGGGGTTAGCACACGGACTTCAAGCATTGGCCACTCCCATGACGTGA | 56 |
|  | Chloroflexi_oe9 | CCCAGTCGGGGAGGCAGCGGCCGAGGGCAGGGGCCGCGACTGGGACGAAGTCGTAAC | 57 |
|  | Chloroflexi_oe10 | AAAGGAGGTGATCCAGCCGCACCTTCCGGTACGGCTACCTTGTTACGACTTCGTCCCA | 58 |
| 19 | Chrysiogenetes_oe1 | CAGCTCGTGTCGTGAGATGTTGGGTTAAGTCCCGCAACGAGCGCAACCCCTGCCATTAGTTGC | 63 |
|  | Chrysiogenetes_oe2 | CCGGATTACTCCGGCTGTCCCACTAGAGTGCCCAACTTAATGATGGCAACTAATGGCAGGG | 61 |
|  | Chrysiogenetes_oe3 | GCCGGAGTAATCCGGAGGAAGGTGGGGACGACGTCAAGTCATCATGGCCCTTATGACCAGG | 61 |
|  | Chrysiogenetes_oe4 | CGAGGTCGCATCCCGTTGTCCTTGCCATTGTAGCACGTGTGTAGCCCTGGTCATAAGGGC | 60 |
|  | Chrysiogenetes_oe5 | GGATGCGACCTCGCGAGAGTGAGCCAACCTCAAAAACCTTGTCTTAGTTCGGATTGCAGTC | 61 |
|  | Chrysiogenetes_oe6 | CTGACCTGCGATTACTAGCGATTCCGACTTCATGCAGTCGAGTTGCAGACTGCAATCCGAACTA | 64 |
|  | Chrysiogenetes_oe7 | GCTAGTAATCGCAGGTCAGCATACTGCGGTGAATACGTTCCCGGGCCTTGTACACACCGCCCG | 63 |
|  | Chrysiogenetes_oe8 | GAAGATTCGGTCACCCGCTTCTGGCAAAACCGACTTTCGTGGTGTGACGGGCGGTGTGTAC | 61 |
|  | Chrysiogenetes_oe9 | GGGTGACCGAATCTTCGGATAGGAGCCTTCGAAGGCAGGACTGGTGATTGGGGTGAAGTCGTA | 63 |
|  | Chrysiogenetes_oe10 | AAAGGAGGTGATCCAGCCGCACCTTCCGATACGGCTACCTTGTTACGACTTCACCCCAATC | 61 |
| 20 | Bacteroidetes_oe1 | TGGTTGTCGTCAGCTCGTGCCGTGAGGTGTCGGCTTAAGTGCCATAACGAGCGCAACCCTTATC | 64 |
|  | Bacteroidetes_oe2 | ACGGCAGTCTCTCCAGAGTCCTCAGCATGACCTGTTAGTAACTGAAGATAAGGGTTGCGCTCG | 63 |
|  | Bacteroidetes_oe3 | TGGAGAGACTGCCGTCGTAAGATGTGAGGAAGGTGGGGATGACGTCAAATCAGCACGGCCC | 61 |
|  | Bacteroidetes_oe4 | AGCTGCCTTCTGTACCCCCCATTGTAACACGTGTGTAGCCCCGGACGTAAGGGCCGTGCTGA | 62 |
|  | Bacteroidetes_oe5 | GGGGTACAGAAGGCAGCTAGCGGGTGACCGTATGCTAATCCCAAAATCCTCTCTCAGTTCGGAT | 64 |
|  | Bacteroidetes_oe6 | GCGATTACTAGCGAATCCAGCTTCACGAAGTCGGGTTGCAGACTTCGATCCGAACTGAGAGAGG | 64 |
|  | Bacteroidetes_oe7 | GATTCGCTAGTAATCGCGCATCAGCCACGGCGCGGTGAATACGTTCCCGGGCCTTGTACACAC | 63 |
|  | Bacteroidetes_oe8 | TTGCGGTTACGTACTTCAGGTACCCCCGGCTCCCATGGCTTGACGGGCGGTGTGTACAAGGCCCG | 65 |
|  | Bacteroidetes_oe9 | TGAAGTACGTAACCGCAAGGATCGTCCTAGGGTAAAACTGGTGACTGGGGCTAAGTCGTAACAAG | 65 |
|  | Bacteroidetes_oe10 | AAAGGAGGTGTTCCAGCCGCACCTTCCGGTACGGCTACCTTGTTACGACTTAGCCC | 56 |
| 21 | Aquificae_oe1 | GTGTCGTGAGATGTTGGGTTAAGTCCCGCAACGAGCGCAACCCTTGTCCTGTGTTACCAGCG | 62 |
|  | Aquificae_oe2 | TCCTCCGGCTTATCGCCGGCAGTCTCCTGTGAGTACCCGGCATTACCCGCTGGTAACACAGG | 62 |
|  | Aquificae_oe3 | CGATAAGCCGGAGGAAGGAGGGGATGACGTCAGATCAGTATGCCCTTTATGCCCTGGGCTA | 61 |
|  | Aquificae_oe4 | CTTACGGCGTTGCAGCCCATTGTCCCTGCCACTGTAGCGCCTGTGTAGCCCAGGGCATAAA | 61 |
|  | Aquificae_oe5 | TGCAACGCCGTAAGGCGGAGCTAATCCCCTAAACCCTGTCGTGGTGCAGATTGAGGGTTGC | 61 |
|  | Aquificae_oe6 | GCTGATTCGCCATTACTACCGATTCCGCCTTCATGAGGGTGAGTTGCAACCCTCAATCTGC | 61 |
|  | Aquificae_oe7 | TAATGGCGAATCAGCAATGTCGCCGTGAATACGTTCCCGGGTCTTGTACACACCGCCCGT | 60 |
|  | Aquificae_oe8 | TTGCAGGTTAGCTCGGGGACTTCCGATGAACCCGACTCCCATGGCGTGACGGGCGGTGTG | 60 |
|  | Aquificae_oe9 | CCGAGCTAACCTGCAAAGGAGGCAGGGGCCGATGATGGGCCTGATGACTGGGGCGAAGTC | 60 |
|  | Aquificae_oe10 | AAAGGAGGTGATCCAGCCCCAGGTTCCCCTAGGGCTACCTTGTTACGACTTCGCCCCA | 58 |
| 22 | Caldiserica_oe1 | ACACCCTATGGCACATGCACAGGTGCTGCATGGTTGTCGTCAGCTCGTGTCGTGAGATGTACGGT | 65 |
|  | Caldiserica_oe2 | AAGCCTATTAGCAACTAAGGGCAGGGGTTGCGCTCGTTCACGGACTTAACCGTACATCTCACGACA | 66 |
|  | Caldiserica_oe3 | CCTTAGTTGCTAATAGGCTTCGGCCTATGCACTCTAAGGGGACTGCCAGCGATAAGCTGGAGGAA | 65 |
|  | Caldiserica_oe4 | GTAGCCCTGGGCATAAGGGCCATGAGGATTTGACGTCATCCCCACCTTCCTCCAGCTTATCGC | 63 |
|  | Caldiserica_oe5 | ATGCCCAGGGCTACACACATGCGACAATGGTCGGGACAATGCGTTGCAAACCAGTAATGGGGA | 63 |
|  | Caldiserica_oe6 | AGTTGCAACCCTCAATCCGTACTGGGGTCGGTTTTTTGCGATTAGCTCCCCATTACTGGTTTGC | 64 |
|  | Caldiserica_oe7 | GATTGAGGGTTGCAACTCACCCTCATGAAGCTGGAGTTGCTAGTAACCGCCGGTCAGCTATACG | 64 |
|  | Caldiserica_oe8 | TGGTGTGACGGGCGGTGTGTACAAGACCCGGGAACGTATTCATCGCCGTATAGCTGACCGGC | 62 |
|  | Caldiserica_oe9 | CGCCCGTCACACCACCCGAGTTGCGTGCACCCGAAGTGGCTCGGTGAGTCACGAAGGTGTG | 61 |
|  | Caldiserica_oe10 | CTCGTACACCTACCTTGTTACGACTTCACCCTCCTCATCACGCACACCTTCGTGACT | 57 |
| 23 | Thermotogae_oe1 | AGCCGGCACAGGTGGTGCACGGCCGTCGTCAGCTCGTGCCGTGAGGTGTTGGGTTAAGTCCCG | 63 |
|  | Thermotogae_oe2 | CCCGGCCGAACCGCTGGCAACTAGGGGCAGGGGTTGCGCTCGTTGCGGGACTTAACCCAA | 60 |
|  | Thermotogae_oe3 | TTCGGCCGGGCACTCTAGGGGGACTGCCGGCGACGAGCCGGAGGAAGGAGGGGATGACG | 59 |
|  | Thermotogae_oe4 | ATTGTAGCGCGTGTGTCGCCCAGGGCATAAGGGGCACGAGTACCTGACGTCATCCCCTCCTT | 62 |
|  | Thermotogae_oe5 | CACACGCGCTACAATGGGCGGTACAATGGGTTGCGACCCCGCGAGGGGGAGCCAATCCCCAA | 62 |
|  | Thermotogae_oe6 | CTTCACGCAGGCGGGTTGCAGCCTGCGATCCGAACTGAGGGCGGTTTTGGGGATTGGCTCC | 61 |
|  | Thermotogae_oe7 | CGCCTGCGTGAAGCCGGAATCGCTAGTAATCGCGGATCAGCCACGCCGCGGTGAATACGTTC | 62 |
|  | Thermotogae_oe8 | GCCCCCGACTCGGGTGGCGTGACGGGCGGTGTGTACAAGGCCCGGGAACGTATTCACCGCG | 61 |
|  | Thermotogae_oe9 | CGAGTCGGGGGCTCCCGAAGACACCTGCCCCAACCCGAAAGGGAGGGGGGGTGTTGAGGG | 60 |
|  | Thermotogae_oe10 | TACGGCTACCTTGTTACGACTTCGCCCCCCTCACCAGGTTCTCCCTCAACACCCCC | 56 |
| 24 | Tenericutes_oe1 | TTGTCGTCAGCTCGTGTCGTGAGATGTTGGGTTAAGTCCCGCAACGAGCGCAACCCTTATC | 61 |
|  | Tenericutes_oe2 | CCTTCCTCCAACTTACGTTGGCAGTCTCGTTAGACAAAGTAACTAACGATAAGGGTTGCGC | 61 |
|  | Tenericutes_oe3 | AACGTAAGTTGGAGGAAGGTGGGGATGACGTCAAATCATCATGCCCCTTATGTCTAGGGCTGCA | 64 |
|  | Tenericutes_oe4 | CCTTACGGATTTGCAACTGTTTGTATTGGCCATTGTAGCACGTTTGCAGCCCTAGACATAAGG | 63 |
|  | Tenericutes_oe5 | GTTGCAAATCCGTAAGGTGGAGCTAATCTGTAAAGTTGGTCTCAGTTCGGATTGAGGGCTG | 61 |
|  | Tenericutes_oe6 | CTGATTCGCGATTACTAGTGATTCCGACTTCATGAGGGCGAATTGCAGCCCTCAATCCG | 59 |
|  | Tenericutes_oe7 | GTAATCGCGAATCAGCCATGTCGCGGTGAATACGTTCTCGGGTCTTGTACACACCGCCCG | 60 |
|  | Tenericutes_oe8 | CGGTTAGCAACACGGTTTTAGATATTACCAGCTCTCATAGTTTGACGGGCGGTGTGTAC | 59 |
|  | Tenericutes_oe9 | CCGTGTTGCTAACCGCAAGGAAGCGCATGTCTAGGGTAGGGCCGGTGATTGGAGTTAAGTCGT | 63 |
|  | Tenericutes_oe10 | AAAGGAGGTAATCCACCCCCACGTTCTCGTAGGGGTACCTTGTTACGACTTAACTCCAATCACC | 64 |
| 25 | Nitrospirae_oe1 | GCAATCACACAGGTGCTGCATGGCTGTCGTCAGCTCGTGCCGTGAGGTGTTGGGTTCAGTCCC | 63 |
|  | Nitrospirae_oe2 | CCGGCTTTACCCGATGGCAACAAAGGGCGAGGGTTGCGCTCGTTGCGGGACTGAACCCAA | 60 |
|  | Nitrospirae_oe3 | CGGGTAAAGCCGGGCACTCTAAGGGGACTGCCAGCGACAAGTTGGAGGAAGGAGAGGATGAC | 62 |
|  | Nitrospirae_oe4 | TTGTTGCACGTGTGTGGCCCTAGGCATAAAGGCCATGATGACTTGACGTCATCCTCTCCTTCC | 63 |
|  | Nitrospirae_oe5 | CCACACACGTGCAACAATGGCCGGTACAGACGGAGGCAATGCCGAGAGGCGGAGCAAACCCGA | 63 |
|  | Nitrospirae_oe6 | CTTCATGAGGTCGAGTTGCAGACCTCAATCCGAACTGGGACCGGTTTTCTCGGGTTTGCTCCG | 63 |
|  | Nitrospirae_oe7 | GCAACTCGACCTCATGAAGTCGGAATCGCTAGTAATCGCATATCAGAACGATGCGGTGAATACGT | 65 |
|  | Nitrospirae_oe8 | TACAACAAACTTTCGTGGTGTGACGGGCGGTGTGTACAAGGCCCGGGAACGTATTCACCGCATCG | 65 |
|  | Nitrospirae_oe9 | ACCACGAAAGTTTGTTGTACCCGAAGTCGGTGCCTTAACCTCGCAAGAGGAGAGAGCCGCCCA | 63 |
|  | Nitrospirae_oe10 | CGGCTACCTTGTTACGACTTCACCCCAATCATCGGCCATACCTTGGGCGGCTCTCT | 56 |
| 26 | Lentisphaerae_oe1 | GTGTTCGGTTAAGTCCGGCAACGAGCGCAACCCATATCCTTACTTGCTAACAGGTAATGCTGAG | 64 |
|  | Lentisphaerae_oe2 | CACACCTTCCTCCCGCTTAACACGGGCAGTCTCCTTAAGGTTCTCAGCATTACCTGTTAGCA | 62 |
|  | Lentisphaerae_oe3 | CGGGAGGAAGGTGTGGACGACGTCAAGTCAGTATGGCCCTTACACCCGGGGCTGCACAC | 59 |
|  | Lentisphaerae_oe4 | TCGCTCCATATCACTATGTCGCTGCCCTTTGTACCGGCCATTGTAGCACGTGTGCAGCCCC | 61 |
|  | Lentisphaerae_oe5 | GACATAGTGATATGGAGCGAATCCCCAAAACCGGTCTCAGTACGGATTGGAGTCTGCAACTCG | 63 |
|  | Lentisphaerae_oe6 | CCGTAGCTGATGCCCATTTACTAGCGATTCCATCTTCATGGAGTCGAGTTGCAGACTCCA | 60 |
|  | Lentisphaerae_oe7 | GGCATCAGCTACGGCTCATTGAATACGTTCCCGGGCCTTGTACACACCGCCCGTCACA | 58 |
|  | Lentisphaerae_oe8 | GCAAGCAGGTTGGCGCAACGACTTCGGGTGAACTCAGCTCCCATGATGTGACGGGCGG | 58 |
|  | Lentisphaerae_oe9 | GCCAACCTGCTTGCAGGAGGCAGACGCCGAAGGTGGGCTTAGTGACTGGGATGAAGTCGTA | 61 |
|  | Lentisphaerae_oe10 | AAAGGAGGTGATCCAGCCGCTGGTTCCCCAACGGCTACCTTGTTACGACTTCATCCCAGTCA | 62 |
| 27 | Chlamydiae_oe1 | AGGTGCTGCATGGCTGTCGTCAGCTCGTGCCGTGAGGTGTTGGGTTAAGTCCCGCAACGAGC | 62 |
|  | Chlamydiae_oe2 | GTCTCGTTAGAGTTCCCACCCTAAGTGCTGGCAACTAACGATAAGGGTTGCGCTCGTTGCGGG | 63 |
|  | Chlamydiae_oe3 | GGTGGGAACTCTAACGAGACTGCCTGGGTTAACCAGGAGGAAGGCGAGGATGACGTCAAGTCAGCA | 66 |
|  | Chlamydiae_oe4 | TCTGTACTGGCCATTGTAGCACGTGTGTCGCCCTGGGCATAAGGGCCATGCTGACTTGACGTCATC | 66 |
|  | Chlamydiae_oe5 | GCTACAATGGCCAGTACAGAAGGTAGCAAGATCGTGAGATGGAGCAAATCCTTAAAGCTGGCCC | 64 |
|  | Chlamydiae_oe6 | CTAGCAATTCCGACTTCATGTAGTCGAGTTGCAGACTACAATCCGAACTGGGGCCAGCTTTAAGG | 65 |
|  | Chlamydiae_oe7 | CATGAAGTCGGAATTGCTAGTAATGGCGTGTCAGCCATAACGCCGTGAATACGTTCCCGGGCC | 63 |
|  | Chlamydiae_oe8 | AACGACTTAAGGTAAAACCAACTCCCATGATGTGACGGGCGGTGTGTACAAGGCCCGGGAACG | 63 |
|  | Chlamydiae_oe9 | TGGTTTTACCTTAAGTCGTTGACTCAACCCGCRAGGGRGAGAGGCGCCCAAGGTGAGGCTGATGAC | 66 |
|  | Chlamydiae_oe10 | CCACCTTCCGGTAGGGCTACCTTGTTACGACTTCATCCTAGTCATCAGCCTCACCTTG | 58 |
| 28 | Planctomycetes_oe1 | GCCGTGAGGTGTTGGGTTAAGTCCCCTAACGAGCGAAACCCCTGTGTCTAGTTGCCAGCGG | 61 |
|  | Planctomycetes_oe2 | TCCTCCGGCTTAACGCCGGCGGTCTGTCTAGAGTTCCCGGCACGACCCGCTGGCAACTAG | 60 |
|  | Planctomycetes_oe3 | CGTTAAGCCGGAGGAAGGCGGGGATGACGTCAAGTCCTCATGGCCCTTATGCTTGGGGCT | 60 |
|  | Planctomycetes_oe4 | TTGCAGCCTAGCAACGCTCTGTCCGCCCCATTGTAGTACGTGTGCAGCCCCAAGCATAAGG | 61 |
|  | Planctomycetes_oe5 | CGTTGCTAGGCTGCAAAGTCATGCTAATCGCAAAAACCGTTCCTCAGTTCGGATTGCGG | 59 |
|  | Planctomycetes_oe6 | TGATCCGCGATTACTAGCGATTCCAGCTTCATGCGGGCGGGTTGCAGCCCGCAATCCGAA | 60 |
|  | Planctomycetes_oe7 | GCTAGTAATCGCGGATCAGCATGCCGCGGTGAATGTGTTCCTGAGCCTTGTACACACCGCC | 61 |
|  | Planctomycetes_oe8 | GGCTCCGGCGACTTCGGATGCCCCCCGCTTTGGTGGCTTGACGGGCGGTGTGTACAAG | 58 |
|  | Planctomycetes_oe9 | CGCCGGAGCCGCAAGGCAGGCGCCGAAGATGAAACCCGTGATGGGGACTAAGTCGTAAC | 59 |
|  | Planctomycetes_oe10 | AAAGGAGGTGATCCAACCGCAGGTTCCCCTACGGTTACCTTGTTACGACTTAGTCCCCA | 59 |
| 29 | Deferribacteres_oe1 | GGGAGACAGGTGCTGCATGGCTGTCGTCAGCTCGTGCCGTGAGGTGTTGGGTTAAGTCCCGCAA | 64 |
|  | Deferribacteres_oe2 | GTGCCCGGCCTAACCGATGGCAACTAAGGGTAGGGGTTGCGCTCGTTGCGGGACTTAACCC | 61 |
|  | Deferribacteres_oe3 | TTAGGCCGGGCACTCTAAGGGGACTGCCCCGGATAACGGGGAGGAAGGTGGGGATGACGTC | 61 |
|  | Deferribacteres_oe4 | CCCATTGTAGCACGTGTGTAGCCCTGGACATAAGGGCCATGATGACTTGACGTCATCCCCACC | 63 |
|  | Deferribacteres_oe5 | CACGTGCTACAATGGGGCGTACAGAGGGCAGCGAAGCCGCGAGGCTGAGCGAATCTCAGAAAG | 63 |
|  | Deferribacteres_oe6 | GGCTTCACGCAGTCGAGTTGCAGACTGCGATCCGAACTGAGGAGCGCTTTCTGAGATTCGCTC | 63 |
|  | Deferribacteres_oe7 | GACTGCGTGAAGCCGGAATCGCTAGTAATCGCAGGTCAGCAAAACTGCGGTGAATACGTTCC | 62 |
|  | Deferribacteres_oe8 | AGGTATAGCCAACTCCCGTGGTGTGACGGGCGGTGTGTACAAGGCCCGGGAACGTATTCACCGC | 64 |
|  | Deferribacteres_oe9 | CGGGAGTTGGCTATACCTGAAGCCGGTGGCCCAACCCAGGCAACTGGGGGGGAGCCGTCCAT | 62 |
|  | Deferribacteres_oe10 | ACGGCTACCTTGTTACGACTTCACCCCAGTCGCCAGCCATACCATGGACGGCTCCC | 56 |
| 30 | Cyanobacteria_oe1 | GCAACCCACGTTTTTAGTTGCCAGCATTTAGTTGGGCACTCTAGAAAGACCGCCGGT | 57 |
|  | Cyanobacteria_oe2 | GGCATGATGACTTGACGTCATCCACACCTTCCTCCGGTTTATCACCGGCGGTCTTT | 56 |
|  | Cyanobacteria_oe3 | TCAAGTCATCATGCCCCTTACATCCTGGGCTACACACGTACTACAATGCTACGGACAAA | 59 |
|  | Cyanobacteria_oe4 | ACGGTTTATGGGATTTGCTTGCTCTCGCGAGCTTGCTGCCCTTTGTCCGTAGCATTGTAG | 60 |
|  | Cyanobacteria_oe5 | CAAATCCCATAAACCGTGGCTCAGTTCAGATCGTAGGCTGCAACTCGCCTACGTGAAGTA | 60 |
|  | Cyanobacteria_oe6 | GTATTCACCGCAGTATGCTGACCTGCGATTACTAGCGATTCCTACTTCACGTAGGCGAGTT | 61 |
|  | Cyanobacteria_oe7 | GCATACTGCGGTGAATACGTTCCCGGGCCTTGTACACACCGCCCGTCACACCATGGAAG | 59 |
|  | Cyanobacteria_oe8 | TCCTCCACAAGGGTTGGAGTAACGACTTCGGGCGTGGCCAACTTCCATGGTGTGACGG | 58 |
|  | Cyanobacteria_oe9 | AACCCTTGTGGAGGAGGACGCCGAAGGTGGGGCTAATGACTGGGGTGAAGTCGTAACAA | 59 |
|  | Cyanobacteria_oe10 | TTAGGAGGTGATCCAGCCGCACCTTCCGGTACGGCTACCTTGTTACGACTTCACCCC | 57 |
| 31 | Fusobacteria_oe1 | TGTCGTGAGATGTTGGGTTAAGTCCCGCAACGAGCGCAACCCCTTTCGTATGTTACCATCATTAA | 65 |
|  | Fusobacteria_oe2 | ACCTTCCTCCTACTCATCGTAGGCAGTATCGCATGAGTCCCCAACTTAATGATGGTAACATACGA | 65 |
|  | Fusobacteria_oe3 | CGATGAGTAGGAGGAAGGTGGGGATGACGTCAAGTCATCATGCCCCTTATACGCTGGGCTACAC | 64 |
|  | Fusobacteria_oe4 | CACCTCACGGCTTTGCAACTCTCTGTTCTACCCATTGTAGCACGTGTGTAGCCCAGCGTATA | 62 |
|  | Fusobacteria_oe5 | AAAGCCGTGAGGTGGAGCTAATCTCAGAAAACTATTCTTAGTTCGGATTGTACTCTGCAACT | 62 |
|  | Fusobacteria_oe6 | CATTGCTGATTCGCGATTACTAGCGATTCCAACTTCATGTACTCGAGTTGCAGAGTACAATCCG | 64 |
|  | Fusobacteria_oe7 | CGCGAATCAGCAATGTCGCGGTGAATACGTTCTCGGGTCTTGTACACACCGCCCGTCAC | 59 |
|  | Fusobacteria_oe8 | CTCCTTACGGTTAGGCCTGCTACTTCAGGTGCAACCAACTCTCGTGGTGTGACGGGCGGT | 60 |
|  | Fusobacteria_oe9 | GGCCTAACCGTAAGGAGGGATGTTCCGAGGGTGTGATTAGCGATTGGGGTGAAGTCGTAACAAG | 64 |
|  | Fusobacteria_oe10 | AAAGGAGGTGATCCATCCGCACGTTCCCGTACGGATACCTTGTTACGACTTCACCC | 56 |
