# Supplementary material for: Prediction of PCR amplification from primer and template sequences using recurrent neural network
Source: Sci Rep. 2021 Apr 5;11:7493. doi: 10.1038/s41598-021-86357-1 (PMC8021588; doi:10.1038/s41598-021-86357-1)
Supplement: Supplementary file 2 — Supplementary Information 2. [file 41598_2021_86357_MOESM2_ESM.docx]

Supplement 2 for “Prediction of PCR amplification from Primer and Template Sequences using Recurrent Neural Network” by Kotetsu Kayama, Miyuki Kanno, Naoto Chisaki, Misaki Tanaka, Reika Yao, Kiwamu Hanazono, Gerry Amor Camer and Daiji Endoh

Procedure for creating pseudo-words and pseudo-sentences.

1. Excision of base sequence from 3'-end of primer

Sequences of oligomers were excised from the 3'-side of the primer sequence and all primers were stored in FASTA format. The excision was performed for 6-bases to 20-bases in length. The full length of the template was stored in FASTA format.

2. Determination of the homologous region of the 3’-end series of the primers the template sequence

With respect to the primer partial sequences prepared in the first step, the localization of all the 3’-end partial sequences of the primers on the templates was searched for by the locate function of the Seqkit program (https://bioinf.shenwei.me/seqkit/). On the homology search, the homologous region in the templates with each 3’-end of primers were searched under the condition in which minimum match on the homology (Table 1). For example, if the 3’-end sequence is 8mer, Seqkit searched homologous region with 0,1 or 2 mutations because minimum number of match is 6 when the base length 8 in Table1. The search results were output as tab-delimited text.

Table 1 Oligomer homology to be analyzed

| Base length of oligomer | Minimum number of homologous bases |
| --- | --- |
| 5 | 4 |
| 6 | 5 |
| 7 | 6 |
| 8 | 6 |
| 9 | 7 |
| 10 | 8 |
| 11 | 8 |
| 12 | 9 |
| 13 | 10 |
| 14 | 10 |
| 15 | 11 |
| 16 | 12 |
| 17 | 13 |
| 18 | 14 |
| 19 | 15 |
| 20 | 16 |
| 21 | 17 |
| 22 | 18 |

3. Generate pentacode

Based on the homology of the base, a homologous code was generated for the homology site of the oligomer selected in the step 3 including the primer set PCR target position, and the code was extracted from the 3 ′ position by 5 characters (pentacode). The pentacode generated the numbers shown in the table according to the number of bases from the 3 'end position of the homologous oligomer at its 3' end position (Table 3). In the generation, the pentacode at the primer set PCR target position was converted to upper case.

Table 3 Number of pentacodes generated according to the 3' terminal position of the pentacode

| Length homology | Position of left-end of pentacode in oligomer | | | | | | | | | | | | | | | | | |
| --- | --- | --- | --- | --- | --- | --- | --- | --- | --- | --- | --- | --- | --- | --- | --- | --- | --- | --- |
|  | 1 | 2 | 3 | 4 | 5 | 6 | 7 | 8 | 9 | 10 | 11 | 12 | 13 | 14 | 15 | 16 | 17 | 18 |
| 5 | 1 |  |  |  |  |  |  |  |  |  |  |  |  |  |  |  |  |  |
| 6 | 2 | 1 |  |  |  |  |  |  |  |  |  |  |  |  |  |  |  |  |
| 7 | 2 | 1 | 1 |  |  |  |  |  |  |  |  |  |  |  |  |  |  |  |
| 8 | 2 | 2 | 1 | 1 |  |  |  |  |  |  |  |  |  |  |  |  |  |  |
| 9 | 3 | 2 | 1 | 1 | 1 |  |  |  |  |  |  |  |  |  |  |  |  |  |
| 10 | 3 | 2 | 2 | 1 | 1 | 1 |  |  |  |  |  |  |  |  |  |  |  |  |
| 11 | 3 | 2 | 2 | 2 | 1 | 1 | 1 |  |  |  |  |  |  |  |  |  |  |  |
| 12 | 3 | 2 | 2 | 2 | 1 | 1 | 1 | 1 |  |  |  |  |  |  |  |  |  |  |
| 13 | 4 | 3 | 2 | 2 | 1 | 1 | 1 | 1 | 1 |  |  |  |  |  |  |  |  |  |
| 14 | 4 | 3 | 2 | 2 | 1 | 1 | 1 | 1 | 1 | 1 |  |  |  |  |  |  |  |  |
| 15 | 4 | 3 | 3 | 2 | 2 | 1 | 1 | 1 | 1 | 1 | 1 |  |  |  |  |  |  |  |
| 16 | 4 | 3 | 3 | 2 | 2 | 2 | 1 | 1 | 1 | 1 | 1 | 1 |  |  |  |  |  |  |
| 17 | 4 | 3 | 3 | 3 | 2 | 2 | 2 | 1 | 1 | 1 | 1 | 1 | 1 |  |  |  |  |  |
| 18 | 5 | 4 | 3 | 3 | 2 | 2 | 2 | 2 | 1 | 1 | 1 | 1 | 1 | 1 |  |  |  |  |
| 19 | 5 | 4 | 3 | 3 | 3 | 2 | 2 | 2 | 2 | 1 | 1 | 1 | 1 | 1 | 1 |  |  |  |
| 20 | 5 | 4 | 4 | 3 | 3 | 3 | 2 | 2 | 2 | 1 | 1 | 1 | 1 | 1 | 1 | 1 |  |  |
| 21 | 5 | 4 | 4 | 3 | 3 | 3 | 2 | 2 | 2 | 2 | 1 | 1 | 1 | 1 | 1 | 1 | 1 |  |
| 22 | 5 | 4 | 4 | 3 | 3 | 3 | 2 | 2 | 2 | 2 | 2 | 1 | 1 | 1 | 1 | 1 | 1 | 1 |

4. Enthalpy and Entropies partially calculated from Horne et al. (2006)

(Horne, M. T., D. J. Fish, and A. S. Benight. "Statistical thermodynamics and kinetics of DNA multiplex hybridization reactions." *Biophysical journal* 91.11 (2006): 4133-4153.)

Enthalpy and Entropy on each dimer of double strand – DNA were calculated by Horne et al. (2006). It is assumed that the values are the same even if the order of the base sequences of the dimers are changed, and the increase due to a mismatch of 1 base would increase as the same manner on 2 bases-mismatch. The extrapolation value for Enthalpy and Entropy were calculated on the assumption that influence of a mismatch is the same as the same base as homologous dimer. For example: Enthalpy and Entropy on AA / AA are predicted to (13.8 kcal/kmol and 36.8 cal/kmol) referring to AA / TT -> (Enthalpy=-7.9 kcal/kmol, Entropy=-22.2 cal/kmol) were changed to AA / TA -> (Enthalpy=1.2 kcal/kmol, Entropy=1.7 cal/kmol).

Table 4 Sequence-dependent thermodynamic parameters for all dimers

| Dimer | | Enthalpy (kcal/kmol) | Entropy (cal/kmol) |
| --- | --- | --- | --- |
| Forward | Reverse |  |  |
| AA | AA | 13.8 | 36.8 |
| AA | AC | 14.9 | 39.7 |
| AA | AG | 12 | 32.8 |
| AA | AT | 4.7 | 12.9 |
| AA | CA | 16.7 | 44.1 |
| AA | CC | 17.8 | 47 |
| AA | CG | 14.9 | 40.1 |
| AA | CT | 7.6 | 20.2 |
| AA | GA | 12.1 | 31.3 |
| AA | GC | 13.2 | 34.2 |
| AA | GG | 10.3 | 27.3 |
| AA | GT | 3 | 7.4 |
| AA | TA | 1.2 | 1.7 |
| AA | TC | 2.3 | 4.6 |
| AA | TG | -0.6 | -2.3 |
| AA | TT | -7.9 | -22.2 |
| AC | AA | 10.8 | 27.2 |
| AC | AC | 5.5 | 8.2 |
| AC | AG | -2.9 | -9.8 |
| AC | AT | 6.2 | 12.8 |
| AC | CA | 13 | 33.2 |
| AC | CC | 7.7 | 14.2 |
| AC | CG | -0.7 | -3.8 |
| AC | CT | 8.4 | 18.8 |
| AC | GA | 14.2 | 40.2 |
| AC | GC | 8.9 | 21.2 |
| AC | GG | 0.5 | 3.2 |
| AC | GT | 9.6 | 25.8 |
| AC | TA | 5.3 | 14.6 |
| AC | TC | 0 | -4.4 |
| AC | TG | -8.4 | -22.4 |
| AC | TT | 0.7 | 0.2 |
| AG | AA | 6.2 | 14.5 |
| AG | AC | -0.9 | -4.2 |
| AG | AG | 3.8 | 7.3 |
| AG | AT | 7.9 | 17.7 |
| AG | CA | 7.7 | 18.1 |
| AG | CC | 0.6 | -0.6 |
| AG | CG | 5.3 | 10.9 |
| AG | CT | 9.4 | 21.3 |
| AG | GA | 3.1 | 5.5 |
| AG | GC | -4 | -13.2 |
| AG | GG | 0.7 | -1.7 |
| AG | GT | 4.8 | 8.7 |
| AG | TA | -0.7 | -2.3 |
| AG | TC | -7.8 | -21 |
| AG | TG | -3.1 | -9.5 |
| AG | TT | 1 | 0.9 |
| AT | AA | 4.7 | 12.9 |
| AT | AC | 10.7 | 27.1 |
| AT | AG | 9.4 | 25 |
| AT | AT | 9.2 | 22.5 |
| AT | CA | 3.4 | 8 |
| AT | CC | 9.4 | 22.2 |
| AT | CG | 8.1 | 20.1 |
| AT | CT | 7.9 | 17.6 |
| AT | GA | 0.7 | 0.7 |
| AT | GC | 6.7 | 14.9 |
| AT | GG | 5.4 | 12.8 |
| AT | GT | 5.2 | 10.3 |
| AT | TA | -7.2 | -20.4 |
| AT | TC | -1.2 | -6.2 |
| AT | TG | -2.5 | -8.3 |
| AT | TT | -2.7 | -10.8 |
| CA | AA | 9 | 20.9 |
| CA | AC | 13.8 | 34.4 |
| CA | AG | 11.2 | 28.4 |
| CA | AT | 3.4 | 8 |
| CA | CA | 11.7 | 29.3 |
| CA | CC | 16.5 | 42.8 |
| CA | CG | 13.9 | 36.8 |
| CA | CT | 6.1 | 16.4 |
| CA | GA | -2.9 | -9.8 |
| CA | GC | 1.9 | 3.7 |
| CA | GG | -0.7 | -2.3 |
| CA | GT | -8.5 | -22.7 |
| CA | TA | 6.3 | 13.1 |
| CA | TC | 11.1 | 26.6 |
| CA | TG | 8.5 | 20.6 |
| CA | TT | 0.7 | 0.2 |
| CC | AA | 18.4 | 48.3 |
| CC | AC | 11.7 | 26.9 |
| CC | AG | 5.2 | 14.2 |
| CC | AT | 12.4 | 29.6 |
| CC | CA | 16.8 | 43 |
| CC | CC | 10.1 | 21.6 |
| CC | CG | 3.6 | 8.9 |
| CC | CT | 10.8 | 24.3 |
| CC | GA | 5.2 | 14.2 |
| CC | GC | -1.5 | -7.2 |
| CC | GG | -8 | -19.9 |
| CC | GT | -0.8 | -4.5 |
| CC | TA | 18.4 | 47.6 |
| CC | TC | 11.7 | 26.2 |
| CC | TG | 5.2 | 13.5 |
| CC | TT | 12.4 | 28.9 |
| CG | AA | 9.3 | 22.4 |
| CG | AC | -0.7 | -3.8 |
| CG | AG | 5 | 8.1 |
| CG | AT | 5.8 | 11.7 |
| CG | CA | 13.6 | 35.1 |
| CG | CC | 3.6 | 8.9 |
| CG | CG | 9.3 | 20.8 |
| CG | CT | 10.1 | 24.4 |
| CG | GA | -0.6 | -1 |
| CG | GC | -10.6 | -27.2 |
| CG | GG | -4.9 | -15.3 |
| CG | GT | -4.1 | -11.7 |
| CG | TA | 8.5 | 20.1 |
| CG | TC | -1.5 | -6.1 |
| CG | TG | 4.2 | 5.8 |
| CG | TT | 5 | 9.4 |
| CT | AA | 7.6 | 20.2 |
| CT | AC | 18.1 | 47.8 |
| CT | AG | 13 | 34.4 |
| CT | AT | 10.8 | 26.6 |
| CT | CA | 6.1 | 16.4 |
| CT | CC | 16.6 | 44 |
| CT | CG | 11.5 | 30.6 |
| CT | CT | 9.3 | 22.8 |
| CT | GA | -8.2 | -22.2 |
| CT | GC | 2.3 | 5.4 |
| CT | GG | -2.8 | -8 |
| CT | GT | -5 | -15.8 |
| CT | TA | 1.2 | 0.7 |
| CT | TC | 11.7 | 28.3 |
| CT | TG | 6.6 | 14.9 |
| CT | TT | 4.4 | 7.1 |
| GA | AA | 8 | 18.7 |
| GA | AC | 14.1 | 37.1 |
| GA | AG | 8.3 | 21.9 |
| GA | AT | 0.7 | 0.7 |
| GA | CA | -0.9 | -4.2 |
| GA | CC | 5.2 | 14.2 |
| GA | CG | -0.6 | -1 |
| GA | CT | -8.2 | -22.2 |
| GA | GA | 8.9 | 21.6 |
| GA | GC | 15 | 40 |
| GA | GG | 9.2 | 24.8 |
| GA | GT | 1.6 | 3.6 |
| GA | TA | 8.3 | 18.9 |
| GA | TC | 14.4 | 37.3 |
| GA | TG | 8.6 | 22.1 |
| GA | TT | 1 | 0.9 |
| GC | AA | 7.7 | 14.9 |
| GC | AC | 4.3 | 4 |
| GC | AG | -4 | -13.2 |
| GC | AT | 8.1 | 16.6 |
| GC | CA | 1.9 | 3.7 |
| GC | CC | -1.5 | -7.2 |
| GC | CG | -9.8 | -24.4 |
| GC | CT | 2.3 | 5.4 |
| GC | GA | 5.7 | 12.3 |
| GC | GC | 2.3 | 1.4 |
| GC | GG | -6 | -15.8 |
| GC | GT | 6.1 | 14 |
| GC | TA | 7.3 | 15.8 |
| GC | TC | 3.9 | 4.9 |
| GC | TG | -4.4 | -12.3 |
| GC | TT | 7.7 | 17.5 |
| GG | AA | 7.8 | 20.8 |
| GG | AC | 0.5 | 3.2 |
| GG | AG | 3.6 | 7.8 |
| GG | AT | 11.8 | 33.5 |
| GG | CA | -0.7 | -2.3 |
| GG | CC | -8 | -19.9 |
| GG | CG | -4.9 | -15.3 |
| GG | CT | 3.3 | 10.4 |
| GG | GA | 1.3 | 1.8 |
| GG | GC | -6 | -15.8 |
| GG | GG | -2.9 | -11.2 |
| GG | GT | 5.3 | 14.5 |
| GG | TA | 10.6 | 28 |
| GG | TC | 3.3 | 10.4 |
| GG | TG | 6.4 | 15 |
| GG | TT | 14.6 | 40.7 |
| GT | AA | 3 | 7.4 |
| GT | AC | 10.7 | 25.6 |
| GT | AG | 7.4 | 18.4 |
| GT | AT | 9.3 | 21.7 |
| GT | CA | -8.5 | -22.7 |
| GT | CC | -0.8 | -4.5 |
| GT | CG | -4.1 | -11.7 |
| GT | CT | -2.2 | -8.4 |
| GT | GA | 1.6 | 3.6 |
| GT | GC | 9.3 | 21.8 |
| GT | GG | 6 | 14.6 |
| GT | GT | 7.9 | 17.9 |
| GT | TA | -0.1 | -1.7 |
| GT | TC | 7.6 | 16.5 |
| GT | TG | 4.3 | 9.3 |
| GT | TT | 6.2 | 12.6 |
| TA | AA | 1.2 | 1.7 |
| TA | AC | 5.3 | 14.6 |
| TA | AG | -0.7 | -2.3 |
| TA | AT | -7.2 | -21.3 |
| TA | CA | 9.6 | 23.7 |
| TA | CC | 13.7 | 36.6 |
| TA | CG | 7.7 | 19.7 |
| TA | CT | 1.2 | 0.7 |
| TA | GA | 8.3 | 21.3 |
| TA | GC | 12.4 | 34.2 |
| TA | GG | 6.4 | 17.3 |
| TA | GT | -0.1 | -1.7 |
| TA | TA | 8.6 | 21.5 |
| TA | TC | 12.7 | 34.4 |
| TA | TG | 6.7 | 17.5 |
| TA | TT | 0.2 | -1.5 |
| TC | AA | 2.3 | 4.6 |
| TC | AC | 0 | -4.4 |
| TC | AG | -7.8 | -21 |
| TC | AT | -1.2 | -6.2 |
| TC | CA | 8.6 | 19.5 |
| TC | CC | 6.3 | 10.5 |
| TC | CG | -1.5 | -6.1 |
| TC | CT | 5.1 | 8.7 |
| TC | GA | 7.3 | 17.6 |
| TC | GC | 5 | 8.6 |
| TC | GG | -2.8 | -8 |
| TC | GT | 3.8 | 6.8 |
| TC | TA | 5.1 | 9.8 |
| TC | TC | 2.8 | 0.8 |
| TC | TG | -5 | -15.8 |
| TC | TT | 1.6 | -1 |
| TG | AA | -0.6 | -2.3 |
| TG | AC | -8.4 | -22.4 |
| TG | AG | -3.1 | -9.5 |
| TG | AT | -2.5 | -8.3 |
| TG | CA | 13 | 33.6 |
| TG | CC | 5.2 | 13.5 |
| TG | CG | 10.5 | 26.4 |
| TG | CT | 11.1 | 27.6 |
| TG | GA | 3.4 | 7.8 |
| TG | GC | -4.4 | -12.3 |
| TG | GG | 0.9 | 0.6 |
| TG | GT | 1.5 | 1.8 |
| TG | TA | 5.6 | 11.7 |
| TG | TC | -2.2 | -8.4 |
| TG | TG | 3.1 | 4.5 |
| TG | TT | 3.7 | 5.7 |
| TT | AA | -7.9 | -22.2 |
| TT | AC | 1 | 0.7 |
| TT | AG | -1.3 | -5.3 |
| TT | AT | -2.7 | -10.8 |
| TT | CA | 1 | 0.7 |
| TT | CC | 9.9 | 23.6 |
| TT | CG | 7.6 | 17.6 |
| TT | CT | 6.2 | 12.1 |
| TT | GA | -1.3 | -5.3 |
| TT | GC | 7.6 | 17.6 |
| TT | GG | 5.3 | 11.6 |
| TT | GT | 3.9 | 6.1 |
| TT | TA | 0.2 | -1.5 |
| TT | TC | 9.1 | 21.4 |
| TT | TG | 6.8 | 15.4 |
| TT | TT | 5.4 | 9.9 |
