# Supplementary material for: Prediction of PCR amplification from primer and template sequences using recurrent neural network
Source: Sci Rep. 2021 Apr 5;11:7493. doi: 10.1038/s41598-021-86357-1 (PMC8021588; doi:10.1038/s41598-021-86357-1)
Supplement: Supplementary file 3 — Supplementary Information 3. [file 41598_2021_86357_MOESM3_ESM.docx]

Supplement3 for “Prediction of PCR amplification from Primer and Template Sequences using Recurrent Neural Network” by Kotetsu Kayama, Miyuki Kanno, Naoto Chisaki, Misaki Tanaka, Reika Yao, Kiwamu Hanazono, Gerry Amor Camer and Daiji Endoh

Ruby and Python scripts for constructing pseudo-words and pseudo-sentences, learning and testing RNN of PyTorch and for analyzing predictions.

These scripts are open under MIT licence (https://opensource.org/licenses/mit-license.php).

A. Set up primer sequences, primer pairs and template seq

**Construction step of pseudo-words and pseudo-sentences**

Data processing was carried out based on Fig.2.

A. Setup primer sequences, primer pairs and template sequence in the proper format.

**Primer and template data**

1) primer.csv : primer name, nucleotide sequence

2) primer_set: number of primer set, primer name

3) template: template number, template name, nucleotide sequence

B. Search extendable hairpins and predict extended sequence for each primer.

**hairpin_derivatives.rb**

**------------------------------------------------------**

# setting prerequisite packages

require 'bio'

require 'csv'

require 'oj'

def hash_to_csv(tgthash, hashname)

CSV.open("#{hashname}.csv","w") do |csv|

tgthash.each{|k,v|

csv << [k,v].flatten

}

end

end

primer_directory="primer_data"

user_data="user_data"

preset_data="preset_data"

compl={"a"=>"t","t"=>"a","g"=>"c","c"=>"g"}

bpair=Oj.load_file("#{preset_data}/basepair_compl.oj",:mode=>:compat)

#read primer set data

primer_set=Hash.new

CSV.foreach("#{user_data}/primer_set.csv") do |row|

unless primer_set[row[0].to_i] then

primer_set[row[0].to_i] = Array.new

end

primer_set[row[0].to_i] << row[1]

end

primer_set_2=primer_set

# p primer_set

# make hash from primer_set data

pname_set=Hash.new

primer_set.each{|setno,pnames|

pnames.each{|pname|

unless pname_set[pname] then

pname_set[pname]=Array.new

end

pname_set[pname] << setno

}

}

# Read primer-sequences

primers=Hash.new # sequence of primers

CSV.foreach("#{user_data}/primer.csv") do |row|

primers[row[0]]=row[1]

end

# create new sequence as the result of elongation from hairpin dimer

hp_elongation=Hash.new # Elongation bases from hairpin form

primers.each{|pname,pseq|

bseq=Bio::Sequence::NA.new(pseq)

# p pseq

# hphead: 5'-end of the primer sequence: 0 to 11-base length

# hptail 3'-end of the primer sequence: 0 to 5-base length

hphead= bseq[0,11]

hptail=bseq.reverse[0,5]

(1..6).to_a.each{|st|

# p hphead[st,5]

bpairzip= hphead[st,5].split("").zip(hptail.split("")) # Candidates for hairpin-stem

matchidx= bpairzip.map{|x| x.join("")}.map{|x| bpair[x]} # Complementarity for hairpin-stem

# p "#{pname} #{matchidx}"

if matchidx[0]==1 && matchidx.inject(:+)>3 then # if complementary-nucleotide > 3 then

p "#{pname} #{st} #{hphead} #{hptail}"

p "#{hphead[st,5]} #{hptail} #{matchidx}"

topcompl= hphead[0..(st-1)].split("").map{|n| compl[n]} #synthesized sequence from hairpin-stem on stem

elongsynth=topcompl.join("").reverse

hp_elongation[pname]=[st,matchidx,topcompl,elongsynth]

end

}

}

hpelg_primer=Hash.new # Derived primer from the synthesis on the stem of hairpin

# Add the synthesized primers to the original data

hp_elongation.each{|pname,d1|

drv_pname="#{pname}_hp"

p "#{pname} #{drv_pname}"

p d1

p "#{primers[pname]} #{pname_set[pname]}"

derive_primer=primers[pname].downcase.concat(d1[3])

hpelg_primer[drv_pname]=derive_primer

pname_set[pname].each{|setno|

primer_set_2[setno]<< drv_pname

}

}

primer_seq_2=Hash.new # Primer sequences of original and hairpin-drived primers

primers.each{|pn,seq|

primer_seq_2[pn]=seq

}

hpelg_primer.each{|pn,seq|

primer_seq_2[pn]=seq

}

# Store data as oj

Oj.to_file("#{primer_directory}/hp_elongation.oj",hp_elongation,:mode=>:compat)

hash_to_csv(hp_elongation, "#{primer_directory}/hp_elongation")

Oj.to_file("#{primer_directory}/hpelg_primer.oj",hpelg_primer,:mode=>:compat)

hash_to_csv(hpelg_primer, "#{primer_directory}/hpelg_primer")

Oj.to_file("#{primer_directory}/primer_seq_2.oj",primer_seq_2,:mode=>:compat)

hash_to_csv(primer_seq_2, "#{primer_directory}/primer_seq_2")

Oj.to_file("#{primer_directory}/primer_set_2.oj",primer_set_2,:mode=>:compat)

hash_to_csv(primer_set_2, "#{primer_directory}/primer_set_2")

----------------------------------------------------------

C. Search extendable dimers and predict extended sequences for each primer set.

**dimer_derivatives.rb**

----------------------------------------------------------------------

require 'bio'

require 'csv'

require 'oj'

def hash_to_csv(tgthash, hashname)

CSV.open("#{hashname}.csv","w") do |csv|

tgthash.each{|k,v|

csv << [k,v].flatten

}

end

end

def dimer_search(ppoint,primer1,primer2)

seq1=primer1

seq2=primer2

dimer_arr=Array.new

fpri=seq1.upcase.split("")

bseq=Bio::Sequence.auto(seq2)

rpri=bseq.reverse.upcase.split("")

# p fpri

# p rpri

len_p=fpri.length

(0..fpri.length-5).each{|i|

dimers= fpri[i..-1].zip(rpri[0..(len_p-i-1)])

# p dimers

dpoint= dimers.map{|pp| ppoint[pp.sort]}.inject(:+)

# p dpoint

cmdpoint=dpoint/(len_p-i-1).to_f

if cmdpoint > 0.8 then

# p "#{i} #{dimers} #{dpoint} #{len_p-i-1} #{cmdpoint}"

dimer_arr << [i,dimers,dpoint,len_p-i-1,cmdpoint]

end

}

return dimer_arr

end

def elongation_primer_2(compl,overhang1,primer1,primer2)

top_seq=primer1[0,overhang1].downcase.split("").map{|n| compl[n]}

p top_seq

deriv_primer2=(top_seq+primer2.split("").reverse).reverse.join("")

return deriv_primer2

end

def elongation_primer_1(compl,overhang2,primer1,primer2)

end_seq=primer2.reverse[overhang2..-1].downcase.split("").map{|n| compl[n]}

deriv_primer1=(primer1.split("")+end_seq).join("")

return deriv_primer1

end

##################################################

primer_directory="primer_data"

user_data="user_data"

preset_data="preset_data"

templ_primer="templ_primer"

# Read preset data

compl={"a"=>"t","t"=>"a","g"=>"c","c"=>"g"}

pair_pt=Hash.new

CSV.foreach("#{preset_data}/pair_pt_compl.csv") do |row|

pair_pt[row[0]]=row[1].to_i

end

ppoint=Hash.new

pair_pt.each{|pair,pt|

ppoint[pair.split("|")]=pt

}

# p ppoint

primer_sq0=Oj.load_file("#{primer_directory}/primer_seq_2.oj",:mode=>:compat)

primer_seq=Hash.new

primer_sq0.each{|pname,seq|

unless pname=="Primer Name" then

primer_seq[pname]=seq

end

}

primer_seq_3=Hash.new

primer_s0=Oj.load_file("#{primer_directory}/primer_set_2.oj",:mode=>:compat)

primer_set=Hash.new

primer_s0.each{|setno,primers|

if setno.to_i > 0 then

primer_set[setno.to_i]=primers

end

}

primer_set_3=Hash.new

# Search dimer and elongate strands on overhang

# Add new elongated primers to the original and hairpin-elongated primers

primer_set.each{|psetno,primers|

p psetno

primer_set_3[psetno]=Array.new

primers.each_with_index{|pname1,idx1|

primer_seq_3[pname1]=primer_seq[pname1]

primer_set_3[psetno] << pname1

primers.each_with_index{|pname2,idx2|

if idx1 <= idx2

primer1=primer_seq[pname1]

primer2=primer_seq[pname2]

dimerinf=dimer_search(ppoint,primer1,primer2)

if dimerinf.length > 0 then

p "#{pname1} #{pname2}"

p "#{primer1} #{primer2}"

p dimerinf

dimerinf.each{|dm|

overhang1=dm[0]

overhang2=primer1.length-dm[0]

if overhang1 > 0 && overhang2 > 0 then # both ends of primer dimer are stickey

p "overhang 1 2"

# additionally synthesized primer from primer2

deriv_primer2=elongation_primer_2(compl,overhang1,primer1,primer2)

# additionally sythesized primer from primer1

p deriv_primer2

primer_seq_3["#{pname1}_dm#{overhang1}"]=deriv_primer2

primer_set_3[psetno] << "#{pname1}_dm#{overhang1}"

elsif overhang1 > 0 && overhang2==0 then

p "overhang 1"

deriv_primer2=elongation_primer_2(compl,overhang1,primer1,primer2)

p deriv_primer2

primer_seq_3["#{pname2}_dm#{overhang1}"]=deriv_primer2

primer_set_3[psetno] << "#{pname2}_dm#{overhang1}"

elsif overhang1 ==0 && overhang2>0 then

p "overhang 2"

deriv_primer1=elongation_primer_1(compl,overhang2,primer1,primer2)

p deriv_primer1

primer_seq_3["#{pname1}_dm#{overhang1}"]=deriv_primer1

primer_set_3[psetno] << "#{pname1}_dm#{overhang1}"

end

}

end

end

}

}

}

# p primer_seq_3

# Store data as oj files

# primer_set_3[psetno] << "#{pname1}_dm#{overhang1}"

Oj.to_file("#{primer_directory}/primer_set_3.oj",primer_set_3,:mode=>:compat)

hash_to_csv(primer_set_3, "#{primer_directory}/primer_set_3")

p primer_set_3

# primer_seq_3[pname1]=primer_seq[pname1]

Oj.to_file("#{primer_directory}/primer_seq_3.oj",primer_seq_3,:mode=>:compat)

hash_to_csv(primer_seq_3, "#{primer_directory}/primer_seq_3")

----------------------------------------------------------

D. Search homologous regions between primers and the template for each primer-set region which length is longer than 6 and number of unmatched bases is fewer than Table 4.

E. Assign pseudo-letters for each selected primer-template homologous regions. The pseudo-letters were determined referring to Table 4.

F. Calculate predicting Gibbs energy on each primer-template homologous region. The Gibbs energy for each homologous region are calculated from divided dimers and predicted dimer-Enthalpy and Entropy (Table 5).

Summary: Make fasta files for homology search using SeqKit (serialize_prim_templ.rb). Homology search using SeqKit and write results in tsv-format (seqkit_locate_primer_serial.rb). And pickup, make homology code and calculate Gibbs energy on each homology (max_homol_seqkit_loc.rb).

**serialize_prim_templ.rb**

**seqkit_locate_primer_serial.rb**

**max_homol_seqkit_loc.rb**

**serialize_prim_templ.rb**

**----------------------------------------------------------------------**

require 'csv'

require 'oj'

require 'fileutils'

primer_directory="primer_data"

user_data="user_data"

preset_data="preset_data"

templ_primer_directory="templ_primer"

# primer_seq and primer_set => seq_no and set_no

# Primer seq

# primer_seq_3[pname1]=primer_seq[pname1]

# primer_seq: aim_1f, GTCCAGGGCTTCACACATGCTA

primer_seq=Oj.load_file("#{primer_directory}/primer_seq_3.oj",:mode=>:compat)

pno_name=Hash.new

pno_seq=Hash.new

pname_no=Hash.new

i=1

primer_seq.each{|pname,seq|

pno_name[i]=pname

pno_seq[i]=seq

pname_no[pname]=i

i+=1

}

Oj.to_file("#{templ_primer_directory}/pname_no.oj",pname_no,:mode=>:compat)

Oj.to_file("#{templ_primer_directory}/pno_name.oj",pno_name,:mode=>:compat)

Oj.to_file("#{templ_primer_directory}/pno_seq.oj",pno_seq,:mode=>:compat)

# Primer set

# primer_set_3[psetno] << "#{pname1}_dm#{overhang1}"

primer_set=Oj.load_file("primer_data/primer_set_3.oj",:mode=>:compat)

pset_pno=Hash.new

primer_set.each{|psetno,pnames|

pset_pno[psetno.to_i]= pnames.map{|x| pname_no[x]}

}

# "1":[1,2],"2":[3,4]

Oj.to_file("#{templ_primer_directory}/pset_pno.oj",pset_pno,:mode=>:compat)

CSV.open("#{templ_primer_directory}/pset_pno.csv","w") do |csv|

pset_pno.each{|psetno,pnos|

csv << [psetno,pnos].flatten

}

end

pno_pset=Hash.new

pset_pno.each{|pset,apno|

apno.each{|pno|

unless pno_pset[pno] then

pno_pset[pno]=Array.new

end

pno_pset[pno] << pset

}

}

p pno_pset

Oj.to_file("#{templ_primer_directory}/pno_pset.oj",pno_pset,:mode=>:compat)

CSV.open("#{templ_primer_directory}/pno_pset.csv","w") do |csv|

pno_pset.each{|psetno,pnos|

csv << [psetno,pnos].flatten

}

end

# Template

tno_seq=Hash.new

tno_name=Hash.new

CSV.foreach("#{user_data}/templ_no_seq.csv") do |row|

tno_seq[row[0].to_i]=row[2]

tno_name[row[0].to_i]=row[1]

end

# Make fasta files for template, primer and primer_serial

File.open("#{templ_primer_directory}/template.fasta","w") do |file|

tno_seq.each{|tno,seq|

file.puts(">#{tno}")

file.puts(seq.upcase)

}

end

# Write primer and 3' part of sequences in fasta-format

prlen=Array.new

File.open("#{templ_primer_directory}/primer.fasta","w") do |file|

pno_seq.each{|pno,seq|

file.puts(">#{pno}")

file.puts(seq.upcase)

prlen << seq.length

}

end

max_len=prlen.max

# p max_len

p0rimer_3end=Hash.new

(5..max_len).to_a.each{|pend|

p0rimer_3end[pend]=Hash.new

pno_seq.each{|pno,seq|

# 3'-end pend-length

p0rimer_3end[pend][pno]=seq.reverse[0,pend].reverse.upcase

}

}

p1rimer_3end=Hash.new

p0rimer_3end.each{|pend,d1|

p1rimer_3end[pend]=Hash.new

d1.each{|pno,seq|

unless p1rimer_3end[pend][seq] then

p1rimer_3end[pend][seq]=Array.new

end

p1rimer_3end[pend][seq] << pno

}

}

p2rimerno_3end=Hash.new

p1rimer_3end.each{|pend,d1|

p2rimerno_3end[pend]=Hash.new

d1.sort_by{|k,v| k}.each_with_index{|d2,idx|

seq=d2[0]

a_pno=d2[1]

p2rimerno_3end[pend][idx]=[seq,a_pno]

}

}

Oj.to_file("#{templ_primer_directory}/p2rimerno_3end.oj",p2rimerno_3end,:mode=>:compat)

p2rimerno_3end.each{|pend,d1|

File.open("#{templ_primer_directory}/primer3end_#{pend}.fasta","w") do |file|

d1.each{|idx,d2|

a_pno=d2[1]

seq=d2[0]

file.puts(">#{idx}_#{a_pno.join("|")}")

file.puts(seq)

}

end

}

**----------------------------------------------------------------------**

**seqkit_locate_primer_serial.rb**

**-----------------------------------------------------------------------**

require "csv"

require "fileutils"

primer_directory="primer_data"

user_data="user_data"

preset_data="preset_data"

templ_primer_directory="templ_primer"

# FileUtils.mkdir_p("locate_train")

# FileUtils.rm(Dir.glob("locate_train/*"))

tolerance=Hash.new

CSV.foreach("#{preset_data}/tolerance.csv") do |row|

tolerance[row[0].to_i]=row[1].to_i

end

primer_serial_files=Dir.glob("#{templ_primer_directory}/primer3end_*.fasta")

# p primer_serial_files

primer_serial_files.each{|psfile|

pserlength=psfile.sub("#{templ_primer_directory}/primer3end_","").sub(".fasta","").to_i

seqkitcommnd= "cat #{user_data}/template.fasta | seqkit locate -f #{psfile} -m #{tolerance[pserlength]} -j 4 -o locate/ploc_#{pserlength}.tsv"

if tolerance[pserlength] then

p seqkitcommnd

system("#{seqkitcommnd} &")

else

p "no tolerance"

end

}

**-----------------------------------------------------------------------**

**max_homol_seqkit_loc.rb**

**-----------------------------------------------------------------------**

require 'csv'

require 'oj'

require 'bio'

def dimer_array(seq)

a_seq=Array.new

(0..seq.length-2).to_a.each{|i|

a_seq << seq[i,2]

}

return a_seq

end

def calc_sum_gibbs(gibbs,pseq,fcompl_tseq)

dimer_set= dimer_array(pseq).zip(dimer_array(fcompl_tseq)).map{|arr| arr.join("|")}

a_gibbs=dimer_set.map{|x| gibbs[x]}

return a_gibbs.inject(:+)

end

########################################################

primer_directory="primer_data"

user_data="user_data"

preset_data="preset_data"

templ_primer_directory="templ_primer"

pos_homol="pos_homol"

loc_files=Dir.glob("locate/ploc_*.tsv") # location on template from seqkit-locate

# Read location from homology results files

p loc_files

loc=Hash.new

loc_files.each{|lf|

homollen=lf.sub("locate/ploc_","").sub(".tsv","").to_i

p homollen

i=0

CSV.foreach(lf,col_sep: "\t") do |row|

# p row

# Hash: loc[templno][primerno][serial_length]=[[direction,start,end],[primer_seq, template_seq]]

if i>0 then

tno=row[0].to_i

sno_pids=row[1]

sno=sno_pids.split("_")[0].to_i

pids=sno_pids.split("_")[1]

# if homollen==5 then

# p "#{homollen} #{pids}"

# end

a_pno=Array.new

if pids=~/\|/ then

# p "#{homollen} #{sno} #{pids}"

a_pno=pids.split("|").map{|x| x.to_i}

else

# p "#{homollen} #{sno} #{pids}"

a_pno << pids.to_i

end

hdir=row[3]

hst=row[4].to_i

hed=row[5].to_i

pseq=row[2]

tseq=row[6]

e3pos=0

if hdir=="+" then

e3pos=hed

else

e3pos=hst

end

a_pno.each{|pno|

unless loc[tno] then

loc[tno]=Hash.new

end

unless loc[tno][pno] then

loc[tno][pno]=Hash.new

end

unless loc[tno][pno][hdir] then

loc[tno][pno][hdir]=Hash.new

end

unless loc[tno][pno][hdir][e3pos] then

loc[tno][pno][hdir][e3pos]=Hash.new

end

if hst then

loc[tno][pno][hdir][e3pos][homollen] = [hst,hed,pseq,tseq] #location of homologies

end

}

end

i+=1

end

}

# loc.each{|tno,d1|

# d1.each{|pno,d2|

# d2.each{|hdir,d3|

# d3.each{|e3pos,d4|

# if tno==1 && pno<3 then

# p "#{hdir} #{e3pos} #{d4}"

# end

# }

# }

# }

# }

pno_pset0=Oj.load_file("#{templ_primer_directory}/pno_pset.oj",:mode=>:compat)

pno_pset=Hash.new

pno_pset0.each{|pno,d1|

pno_pset[pno.to_i]=d1.map{|x| x.to_i}

}

# p pno_pset

loc3end=Hash.new

loc.each{|tno,d1|

loc3end[tno]=Hash.new

d1.each{|pno,d2|

a_pset=pno_pset[pno]

a_pset.each{|pset|

# p "#{tno} #{pno} #{pset}"

unless loc3end[tno][pset] then

loc3end[tno][pset]=Hash.new

end

d2.each{|hdir,d3|

unless loc3end[tno][pset][hdir] then

loc3end[tno][pset][hdir]=Hash.new

end

d3.each{|e3pos,d4|

unless loc3end[tno][pset][hdir][e3pos] then

loc3end[tno][pset][hdir][e3pos]=Hash.new

end

if d4 then

d4.each{|homollen,d5|

if d5[3].length==homollen then

pno_len="#{pno}_#{homollen}"

loc3end[tno][pset][hdir][e3pos][pno_len]=d5

end

}

end

}

}

}

}

}

# loc3end.each{|tno,d1|

# d1.each{|pset,d2|

# d2.each{|hdir,d3|

# d3.each{|e3pos,d4|

# if tno==1 && pset < 3 then

# p "#{tno} #{pset} #{e3pos} #{hdir}"

# p d4

# end

# }

# }

# }

# }

Oj.to_file("#{pos_homol}/loc.oj",loc,:mode=>:compat)

Oj.to_file("#{pos_homol}/loc3end.oj",loc3end,:mode=>:compat)

# p loc3end

# Select longest homology

loc3end_lgt=Hash.new # longest homologies in loc3end

# loc3end[tno][pset][hdir][e3pos][pno_len]=d5

loc3end.each{|tno,d1|

loc3end_lgt[tno]=Hash.new

d1.each{|pset,d2|

loc3end_lgt[tno][pset]=Hash.new

d2.each{|hdir,d3|

loc3end_lgt[tno][pset][hdir]=Hash.new

d3.each{|e3pos,d4|

loc3end_lgt[tno][pset][hdir][e3pos]=d4.sort_by{|k,v| -k.split("_")[1].to_i}[0]

}

}

}

}

# loc3end_lgt.each{|tno,d1|

# d1.each{|pset,d2|

# d2.each{|hdir,d3|

# if tno==1 && pset==1 then

# p "#{tno} #{pset} #{hdir}"

# p d3

# end

# }

# }

# }

Oj.to_file("#{pos_homol}/loc3end_lgt.oj",loc3end_lgt,:mode=>:compat)

# Select smallest free energy

dimer_set_ee=Hash.new

i=0

CSV.foreach("#{preset_data}/entropy_and_enthalpy_all_dimers.csv") do |row|

if i>0 then

dimer_set_ee["#{row[0]}|#{row[1]}"]=[row[2].to_f,row[3].to_f]

end

i+=1

end

absT=56.0+273.15 # set absolute temprature

gibbs=Hash.new

i=0

dimer_set_ee.each{|bset,ee|

gibbs[bset]=ee[0]-absT*ee[1]/1000 # Gibbs energies for all complementary dimers

}

# p gibbs

# loc3end[tno][pset][hdir][e3pos][pno_len]=d5

loc3gibbs=Hash.new

loc3end.each{|tno,d1|

loc3gibbs[tno]=Hash.new

d1.each{|pset,d2|

loc3gibbs[tno][pset]=Hash.new

d2.each{|hdir,d3|

loc3gibbs[tno][pset][hdir]=Hash.new

d3.each{|e3pos,d4|

loc3gibbs[tno][pset][hdir][e3pos]=Hash.new

d4.each{|pno_len,d5|

hst,hed,pseq,tseq=d5[0],d5[1],d5[2],d5[3]

# change a degenerate nucleotide to a normal base

if tseq=~/(R|W|V|H|D|N)/ then

tseq=tseq.gsub(/(R|W|V|H|D|N)/,"A")

elsif tseq=~/(Y|K|B)/ then

tseq=tseq.gsub(/(Y|K)/,"T")

else

tseq=tseq.gsub(/(S|B)/,"G")

end

fcompl_tseq=Bio::Sequence.auto(tseq).forward_complement.upcase

# p d5

s_gibbs= calc_sum_gibbs(gibbs,pseq,fcompl_tseq)

loc3gibbs[tno][pset][hdir][e3pos][pno_len] = [s_gibbs,hst,hed,pseq,fcompl_tseq]

# p "#{tno} #{pno} #{e3pos} #{d4.sort_by{|x| -x[0]}[0]}"

}

}

}

}

}

# loc3gibbs.each{|tno,d1|

# d1.each{|pset,d2|

# d2.each{|hdir,d3|

# d3.each{|e3pos,d4|

# if tno==1 && pset==1 then

# # p "#{tno} #{pset} #{hdir} #{e3pos}"

# # p d4

# end

# }

# }

# }

# }

Oj.to_file("#{pos_homol}/l3gibbs.oj",loc3gibbs,:mode=>:compat)

# loc3gibbs[tno][pset][hdir][e3pos][pno_len] = [s_gibbs,hst,hed,pseq,fcompl_tseq]

l3g_smlst=Hash.new

loc3gibbs.each{|tno,d1|

l3g_smlst[tno]=Hash.new

d1.each{|pset,d2|

l3g_smlst[tno][pset]=Hash.new

d2.each{|hdir,d3|

l3g_smlst[tno][pset][hdir]=Hash.new

d3.each{|e3pos,d4|

mingibbs=d4.sort_by{|k,v| v[0]}[0]

if mingibbs then

l3g_smlst[tno][pset][hdir][e3pos]=d4.sort_by{|k,v| v[0]}[0]

end

}

}

}

}

l3g_smlst.each{|tno,d1|

d1.each{|pset,d2|

d2.each{|hdir,d3|

d3.each{|e3pos,d4|

if tno==1 && pset==1 then

p "#{tno} #{pset} #{e3pos} "

p d4

end

}

}

}

}

# l3g_smlst[tno][pset][hdir][e3pos]=min_gibbs[s_gibbs,hst,hed,pseq,fcompl_tseq]

Oj.to_file("#{pos_homol}/l3g_smlst.oj",l3g_smlst,:mode=>:compat)

# loc3g_smlst: template number=>homology_directory=>position of homology = most important homology data

**-----------------------------------------------------------------------**

G. Select priming sites on each primer-set-template and capitalize the pseudo-letters on each homologous region which Gibbs energy is smallest in each direction.

I. Create penta-codes and repeated basically according to the length of homologous region.

J. Assign pseudo-letters to entire primers which suggested to prime PCR

Repeat number of primer-template penta-codes are determined according to base-distance from 3’-end of the homologous region (Table 6).

**assumed_pcr_position.rb**

**-----------------------------------------------------------------------**

require 'csv'

require 'oj'

require 'bio'

def dimer_array(seq)

a_seq=Array.new

(0..seq.length-2).to_a.each{|i|

a_seq << seq[i,2]

}

return a_seq

end

def calc_sum_gibbs(gibbs,pseq,fcompl_tseq)

dimer_set= dimer_array(pseq).zip(dimer_array(fcompl_tseq)).map{|arr| arr.join("|")}

a_gibbs=dimer_set.map{|x| gibbs[x]}

return a_gibbs.inject(:+)

end

######################################

user_data="user_data"

preset_data="preset_data"

primer_directory="primer_data"

templ_primer_directory="templ_primer"

pos_homol="pos_homol"

pseudo_sentense="pseudo_sentense"

pair_cd=Oj.load_file("#{preset_data}/pair_cd.oj",:mode=>:compat)

# p pair_cd["mr"]

# l3g_smlst[tno][pset][hdir][e3pos]=min_gibbs[s_gibbs,hst,hed,pseq,fcompl_tseq]

l3g_smlst=Oj.load_file("#{pos_homol}/l3g_smlst.oj",:mode=>:compat)

pno_seq=Oj.load_file("templ_primer/pno_seq.oj",:mode=>:compat)

primercd_mf={"A"=>"p","T"=>"p","G"=>"q","C"=>"q"}

primercd_mr={"A"=>"u","T"=>"u","G"=>"v","C"=>"v"}

# Reconstruct data in a hash for analysis

# l3g_smlst[tno][pset][hdir][e3pos]=min_gibbs([pno_len,[s_gibbs,hst,hed,pseq,fcompl_tseq]])

loc3_wd=Hash.new

l3g_smlst.each{|tno,d1|

loc3_wd[tno.to_i]=Hash.new

d1.each{|psetno,d2|

loc3_wd[tno.to_i][psetno.to_i]=Hash.new

d2.each{|hdir,d3|

loc3_wd[tno.to_i][psetno.to_i][hdir]=Hash.new

d3.each{|e3pos,d4|

# p d4

pno_len=d4[0]

pno=pno_len.split("_")[0].to_i

len_homol=pno_len.split("_")[1].to_i

hominf=d4[1]

# p hominf

mingibbs,pseq,fcompl_tseq=hominf[0].to_f,hominf[3],hominf[4]

if mingibbs < -1.0 then

unless loc3_wd[tno.to_i][psetno.to_i][hdir][e3pos.to_i] then

loc3_wd[tno.to_i][psetno.to_i][hdir][e3pos.to_i]=Hash.new

end

# p mingibbs

a_pairseq=pseq.split("").zip(fcompl_tseq.split("")).map{|x| x.join("|")}

if hdir=="+" then

ps_letters=a_pairseq.map{|x| pair_cd["f"][x]}.join("")

loc3_wd[tno.to_i][psetno.to_i][hdir][e3pos.to_i]=[pno,len_homol,hominf,ps_letters].flatten

else

# p "#{tno} #{psetno} #{hdir} #{d4[4]} #{d4[5]}"

ps_letters=a_pairseq.map{|x| pair_cd["r"][x]}.reverse.join("")

loc3_wd[tno.to_i][psetno.to_i][hdir][e3pos.to_i]=[pno,len_homol,hominf,ps_letters].flatten

end

end

# loc3_wd[tno][psetno][e3pos][hdir]=

}

}

}

}

Oj.to_file("#{pseudo_sentense}/loc3_wd.oj",loc3_wd,:mode=>:compat)

loc3_wd.each{|tno,d1|

d1.each{|psetno,d2|

d2.each{|hdir,d3|

# p "#{tno} #{psetno} #{hdir} #{d3.length}"

d3.each{|e3pos,d4|

if tno==23 && psetno==59 then

p "#{tno} #{psetno} #{hdir} #{e3pos} #{d4}"

end

}

}

}

}

# Record primer positions on assumed PCR

pcr_cand1=Hash.new

loc3_wd.each{|tno,d1|

pcr_cand1[tno]=Hash.new

d1.each{|psetno,d2|

pcr_cand1[tno][psetno]=Hash.new

# p "#{tno} #{psetno}"

d2["+"].each{|e3pos_pl,d4_p|

d2["-"].each{|e3pos_mn,d4_m|

if e3pos_mn-e3pos_pl > 99 then

# p "#{e3pos_pl} #{e3pos_mn}"

pcr_cand1[tno][psetno][[e3pos_pl,e3pos_mn]]=[d4_p[2]+d4_m[2],[d4_p,d4_m]]

end

}

}

}

}

# Make data for gibbs plot

gibbs_plot=Hash.new

p2cr_cand2=Hash.new

pcr_cand1.each{|tno,d1|

p2cr_cand2[tno]=Hash.new

d1.each{|psetno,d2|

# p "#{tno} #{psetno} #{d2.length}"

if d2.length > 0 then

# p "#{tno} #{psetno} #{d2.length}"

min_set=d2.sort_by{|k,v| v[0]}[0]

p2cr_cand2[tno][psetno]= min_set

# select pair sum of which gibbs energy is minimum

# p d2.sort_by{|k,v| v[0]}[0]

unless gibbs_plot[1] then

gibbs_plot[1]=Hash.new

end

gibbs_plot[1]["#{tno}_#{psetno}"]=min_set

else

p "No targets #{tno} #{psetno} #{d2}"

p l3g_smlst[tno.to_s][psetno.to_s]

unless gibbs_plot[0] then

gibbs_plot[0]=Hash.new

end

gibbs_plot[0]["#{tno}_#{psetno}"]=l3g_smlst[tno.to_s][psetno.to_s]

end

}

}

tmppset_pcr=Hash.new

p2cr_cand2.each{|tno,d1|

d1.each{|psetno,d2|

# p "#{tno} #{psetno}"

if d2 then

e3pos_plus=d2[0][0]

e3pos_minus=d2[0][1]

tmppset_pcr[[tno,psetno,e3pos_plus]]="+"

tmppset_pcr[[tno,psetno,e3pos_minus]]="-"

else

unless gibbs_plot[0] then

gibbs_plot[0]=Hash.new

end

p "no target"

# l3g_smlst[tno][psetno][hdir][e3pos]=min_gibbs([pno_len,[s_gibbs,hst,hed,pseq,fcompl_tseq]])

end

}

}

Oj.to_file("#{pseudo_sentense}/pcr_cand1.oj",pcr_cand1,:mode=>:compat)

Oj.to_file("#{pseudo_sentense}/p2cr_cand2.oj",p2cr_cand2,:mode=>:compat)

Oj.to_file("#{pseudo_sentense}/tmppset_pcr.oj",tmppset_pcr,:mode=>:compat)

# gibbs plot: data for scatter plot

Oj.to_file("#{pseudo_sentense}/gibbs_plot.oj",gibbs_plot,:mode=>:compat)

# loc3_wd.each{|tno,d1|

# d1.each{|psetno,d2|

# d2.each{|hdir,d3|

# d3.each{|e3pos,d4|

# if tmppset_pcr[[tno,psetno,e3pos]] then

# p "#{tno} #{psetno} #{hdir} #{e3pos}"

# end

# }

# }

# }

# }

# loc3_wd[tno.to_i][psetno.to_i][hdir][e3pos.to_i]=[pno,len_homol,s_gibbs,hst,hed,pseq,fcompl_tseq,ps_letters]

# [132, 9, -0.38759300000000048e1, 113, 121, "ACGACATAC", "TGCAGTAGG", "ghffghggf"]

# Realign pseudo-words

pcr_wd1=Hash.new

loc3_wd.each{|tno,d1|

pcr_wd1[tno]=Hash.new

d1.each{|psetno,d2|

pcr_wd1[tno][psetno]=Array.new

d2.each{|hdir,d3|

d3.each{|e3pos,d4|

pno=d4[0]

ps_code=d4[7]

gibbs=d4[2]

if tmppset_pcr[[tno,psetno,e3pos]]=="+" then

pcr_wd1[tno][psetno] << [hdir,pno,e3pos,ps_code.upcase,gibbs]

elsif tmppset_pcr[[tno,psetno,e3pos]]=="-" then

pcr_wd1[tno][psetno] << [hdir,pno,e3pos,ps_code.upcase,gibbs]

else

pcr_wd1[tno][psetno] << [hdir,pno,e3pos,ps_code,gibbs]

end

}

}

}

}

# pcr_wd1.each{|tno,d1|

# d1.each{|psetno,d2|

# p "#{tno} #{psetno} #{d2}"

# }

# }

# d2

# [["+", 103, 30, "bbebaa"], ["+", 100, 35, "baacbebb"], ["-", 100, 40, "hgggfff"],

#["-", 103, 67, "ffghggf"], ["-", 103, 94, "hfghgifggfggf"], ["-", 100, 133, "ggggjff"],

#["-", 100, 134, "hgggff"], ["+", 103, 157, "badbabac"], ["+", 103, 166, "beabaa"],

#["+", 100, 171, "ABAACBBBE"], ["-", 103, 193, "hfghggiggfg"], ["-", 103, 196, "hhgfggfg"],

#["-", 100, 329, "GGGGHF"], ["+", 103, 344, "bbabea"], ["-", 103, 412, "ffghgg"]]

Oj.to_file("#{pseudo_sentense}/pcr_wd1.oj",pcr_wd1,:mode=>:compat)

# Make codes and pseuro-words for middle step of PCR

# Reconstruct pseudo-words to pseudo-sentences

pcr_wd2=Hash.new

pcr_wd1.each{|tno,d1|

pcr_wd2[tno]=Hash.new

d1.each{|psetno,d2|

pcr_wd2[tno][psetno]=Array.new

d2.sort_by{|wdd| wdd[2]}.each{|wdd|

# p wdd

if wdd[3][0]=~/[A-Z]/ then

pcr_wd2[tno][psetno] << wdd[3]

if wdd[0]=="+" then

tpgdata= loc3_wd[tno][psetno][wdd[0]][wdd[2]]

mid_cd=tpgdata[5].split("").map{|n| primercd_mf[n]}.join("")

# p mid_cd

pcr_wd2[tno][psetno] << mid_cd

elsif wdd[0]=="-" then

tpgdata= loc3_wd[tno][psetno][wdd[0]][wdd[2]]

mid_cd=tpgdata[5].split("").map{|n| primercd_mr[n]}.join("")

pcr_wd2[tno][psetno] << mid_cd

end

else

pcr_wd2[tno][psetno] << wdd[3]

end

}

}

}

# {"1":{"1":["abcbbab","jfggfjgfg","gfggfhffgj","cabbab","hfggfgf","aababbac","BABBABBBBAABABABAABBAA",

# "qpqqpqqqqppqpqpqppqqpp","hfggifgfg","baabadbac","giggfgf","GFGGFGFFGFFFGFFGGFFFGF","uvuuuvvuuvuuuvuuvuvvuv"],

# "2":["daabaa","bbaaeacaa","ABBBBAABBBAEEEBAAAABAA","pqqqqppqqqpqpqqppppqpp","cbbaaaabae","ghfffg","HFFFFGHGF",

# "vvvuvvvvuuuuuuvuvuuuuv","abaaeab","aaebaa","figjhffggfgigg"],

# pcr_wd2.each{|tno,d1|

# d1.each{|psetno,d2|

# p "#{tno} #{psetno}"

# p d2

# }

# }

Oj.to_file("#{pseudo_sentense}/pcr_wd2.oj",pcr_wd2,:mode=>:compat)

**-----------------------------------------------------------------------**

H. Search hairpin and dimers on each extended primer-sets and assign pseudo-letters for each hairpin or dimer.

Summary: Prepare fasta files for SeqKit-derived homology search to search hairpin and dimers (**primer_fasta_for_hairpin_dimer.rb**). Predict hairpin and dimers to prepare pseudo-words (**hairpin_dimer_cds_for_ps.rb**).

**primer_fasta_for_hairpin_dimer.rb**

**-----------------------------------------------------------------------**

require 'csv'

require 'bio'

require 'oj'

user_data="user_data"

preset_data="preset_data"

primer_directory="primer_data"

templ_primer="templ_primer"

pos_homol="pos_homol"

pseudo_sentense="pseudo_sentense"

hairpin_dimer="hairpin_dimer"

tolerance=Hash.new

CSV.foreach("#{preset_data}/tolerance.csv") do |row|

tolerance[row[0].to_i]=row[1].to_i

end

pset_pno=Oj.load_file("#{templ_primer}/pset_pno.oj")

pno_seq=Oj.load_file("#{templ_primer}/pno_seq.oj",:mode=>:compat)

# Make fasta from 5'end sequence of primers

File.open("#{hairpin_dimer}/pno_seq_top.fasta","w") do |file|

pno_seq.each{|pno,seq|

file.puts(">#{pno}_top")

stemlen=(seq.length-6)/2

file.puts(seq.upcase[0,stemlen])

}

end

# Make fasta from 3'end sequence of primers

File.open("#{hairpin_dimer}/pno_seq_end.fasta","w") do |file|

pno_seq.each{|pno,seq|

file.puts(">#{pno}_end")

stemlen=(seq.length-6)/2

file.puts(seq.upcase[-1*stemlen,stemlen])

}

end

**-----------------------------------------------------------------------**

**hairpin_dimer_cds_for_ps.rb**

**-----------------------------------------------------------------------**

require 'csv'

require 'bio'

require 'oj'

user_data="user_data"

preset_data="preset_data"

primer_data="primer_data"

templ_primer="templ_primer"

pos_homol="pos_homol"

pseudo_sentense="pseudo_sentense"

hairpin_dimer="hairpin_dimer"

# SeqKit homology search on 5'end and 3'end of primers including derived primers

pair_cd=Oj.load_file("#{preset_data}/pair_cd.oj",:mode=>:compat)

p pair_cd["p"]

seqkitcommnd= "cat #{hairpin_dimer}/pno_seq_top.fasta | seqkit locate -f #{hairpin_dimer}/pno_seq_end.fasta -m 2 -j 4 -o #{hairpin_dimer}/primer_homol_topend.tsv"

system(seqkitcommnd)

# Read SeqKit homology data

homolreg=Hash.new

CSV.foreach("#{hairpin_dimer}/primer_homol_topend.tsv",col_sep: "\t") do |row|

if row[3]=="-" then

top_pno=row[0].sub("_top","").to_i

end_pno=row[1].sub("_end","").to_i

top_compl=row[2]

end_fow=row[6]

homolreg[[top_pno,end_pno]]=[top_compl,end_fow]

end

end

# Create codes for hairpin

hairpin_code=Hash.new

homolreg.each{|k,v|

if k[0]==k[1] then

# p k[0]

bseq=Bio::Sequence.auto(v[1])

a_seq_compl=v[0].split("").zip(bseq.forward_complement.upcase.split(""))

hairpin_code[k[0].to_i]= a_seq_compl.map{|n| pair_cd["p"][n.join("|")]}.join("")

end

}

# Create codes for dimers

pset_pno=Oj.load_file("#{templ_primer}/pset_pno.oj")

# p pset_pno

hpdm_code=Hash.new

dimer_code=Hash.new

pset_pno.each{|setno,pset|

pset.each{|p1|

if hairpin_code[p1] then

unless hpdm_code[setno] then

hpdm_code[setno] =Array.new

end

hpdm_code[setno] << hairpin_code[p1].upcase

end

pset.each{|p2|

if p1 != p2 then

if homolreg[[p1,p2]] then

p "#{setno} #{p1} #{p2}"

stm=homolreg[[p1,p2]]

bseq=Bio::Sequence.auto(stm[1])

a_seq_compl=stm[0].split("").zip(bseq.forward_complement.upcase.split(""))

dimer_code[[p1,p2]]= a_seq_compl.map{|n| pair_cd["p"][n.join("|")]}.join("")

unless hpdm_code[setno] then

hpdm_code[setno] =Array.new

end

hpdm_code[setno] << dimer_code[[p1,p2]]

end

end

}

}

}

hpdm_code.each{|setno,code|

p setno

p code

}

Oj.to_file("#{pseudo_sentense}/hpdm_code.oj",hpdm_code,:mode=>:compat)

# p pair_cd["p"]

**-----------------------------------------------------------------------**

Repeat number of hairpin and dimer penta-codes are determined according to the base-length of the hairpin stem or dimer-region (Table 7).

K. Assemble penta-codes on each primer-set-template in order of hairpin, dimer and positions of the homologous region on the template

**reconstruct_words_sentences.rb**

**-----------------------------------------------------------------------**

require 'csv'

require 'bio'

require 'oj'

user_data="user_data"

preset_data="preset_data"

primer_data="primer_data"

templ_primer="templ_primer"

pos_homol="pos_homol"

pseudo_sentense="pseudo_sentense"

hairpin_dimer="hairpin_dimer"

pair_cd=Oj.load_file("#{preset_data}/pair_cd.oj",:mode=>:compat)

hpdm_code=Oj.load_file("#{pseudo_sentense}/hpdm_code.oj",:mode=>:compat)

pcr_wd2=Oj.load_file("#{pseudo_sentense}/pcr_wd2.oj",:mode=>:compat)

i=0

end_position=Array.new

penta_multiplicity=Hash.new

CSV.foreach("#{preset_data}/factor_npenta.csv") do |row|

if i==0 then

end_position=row[1..-1].map{|x| x.to_i}

else

penta_multiplicity[row[0].to_i]=row[1..-1].compact.map{|x| x.to_i}

end

i+=1

end

penta_multi_hd=Hash.new

penta_multiplicity.each{|opsw,d1|

penta_multi_hd[opsw]=d1[0]

}

penta_hairpin_dimer=Hash.new

penta_words=Hash.new

p hpdm_code

(1..31).to_a.each{|tno|

penta_words[tno.to_i]=Hash.new

penta_hairpin_dimer[tno.to_i]=Hash.new

hpdm_code.each{|psetno,d1|

unless penta_words[tno.to_i][psetno.to_i] then

penta_words[tno.to_i][psetno.to_i]=Array.new

end

unless penta_hairpin_dimer[tno.to_i][psetno.to_i] then

penta_hairpin_dimer[tno.to_i][psetno.to_i]=Array.new

end

if d1 then

d1.each{|wd|

# p wd

lwd=wd.length

wdr= wd.reverse

(0..lwd-5).to_a.each_with_index{|i,idx|

p "#{lwd} #{penta_multi_hd[lwd]}"

penta_multi_hd[lwd].times{

penta_words[tno.to_i][psetno.to_i] << wdr[i,5].reverse

penta_hairpin_dimer[tno.to_i][psetno.to_i] << wdr[i,5].reverse

}

}

}

end

}

}

Oj.to_file("#{pseudo_sentense}/penta_hairpin_dimer.oj",penta_hairpin_dimer,:mode=>:compat)

# penta_words.each{|tno,d1|

# d1.each{|psetno,d2|

# p "#{tno} #{psetno}"

# p d2

# }

# }

# divide data (template no - primer set number) into 5 groups

penta_words_pbind=Hash.new

pcr_wd2.each{|tno,d1|

unless penta_words[tno.to_i] then

penta_words[tno.to_i]=Hash.new

end

penta_words_pbind[tno.to_i]=Hash.new

d1.each{|psetno,d2|

unless penta_words[tno.to_i][psetno.to_i] then

penta_words[tno.to_i][psetno.to_i]=Array.new

end

penta_words_pbind[tno.to_i][psetno.to_i]=Array.new

d2.each{|wd|

lwd=wd.length

wdr= wd.reverse

(0..lwd-5).to_a.each_with_index{|i,idx|

penta_multiplicity[lwd][idx].times{

penta_words[tno.to_i][psetno.to_i] << wdr[i,5].reverse

penta_words_pbind[tno.to_i][psetno.to_i] << wdr[i,5].reverse

}

}

}

}

}

CSV.open("#{pseudo_sentense}/panta_words.csv","w") do |csv|

penta_words.each{|tno,d1|

d1.each{|psetno,d2|

p psetno

p d2

csv << [tno,psetno,d2.join(" ")]

}

}

end

Oj.to_file("#{pseudo_sentense}/penta_words.oj",penta_words,:mode=>:compat)

Oj.to_file("#{pseudo_sentense}/penta_words_pbind.oj",penta_words_pbind,:mode=>:compat)

**-----------------------------------------------------------------------**

**Placement of pseudo-sentence for split validation and RNN learning and validation**

Summary: Divide the pseudo-sentence evenly for the template (**construct_training_data.rb**) and then place it in the folder specified by the RNN for validation and RNN learning and validation (**store_training_data.rb**).

**construct_training_data.rb**

**-----------------------------------------------------------------------**

require 'csv'

require 'oj'

user_data="user_data"

preset_data="preset_data"

primer_directory="primer_data"

templ_primer_directory="templ_primer"

pos_homol="pos_homol"

pseudo_sentense="pseudo_sentense"

########################################3

# Read pcr results from csv

i=0

a_templ=Array.new

pcr_r=Hash.new

CSV.foreach("#{user_data}/pcr_results.csv") do |row|

if i==0 then

a_templ=row[1..-1].map{|x| x.to_i}

else

psetno=row[0].to_i

pcr_r[psetno]=Hash.new

row[1..-1].map{|x| x.to_i}.each_with_index{|r,idx|

pcr_r[psetno][a_templ[idx]]=r

}

end

i+=1

end

# Make a Hash

pcr_ts=Hash.new

pcr_r.each{|psetno,d1|

d1.each{|tno,r|

unless pcr_ts[tno] then

pcr_ts[tno]=Hash.new

end

pcr_ts[tno][psetno]=r

}

}

# Set group on each template

k_grp_0=Hash.new

grp=[0,1,2,3,4] # Set groups to 5

pcr_ts.each{|tno,d1|

k_grp_0[tno]=Hash.new

[0,1].each{|pn|

k_grp_0[tno][pn]=Hash.new

tgt_grp=d1.select{|k,v| v==pn}

if tgt_grp then

d1.select{|k,v| v==pn}.to_a.shuffle.each_with_index{|e,idx| # e -> primer-set number

grpno=idx.modulo(5)

unless k_grp_0[tno][pn][grpno] then

k_grp_0[tno][pn][grpno]=Array.new

end

psetno=e[0]

k_grp_0[tno][pn][grpno] << psetno # groups were set on positive/negative -> group number

}

end

}

}

# Make data for grouping of primer pair-template

k1grp=Hash.new

res_k1grp=Hash.new

k_grp_0.each{|tno,d1|

d1.each{|pn,d2|

p d2

unless k1grp[pn] then

k1grp[pn]=Hash.new

end

# p (0..4).to_a.zip((0..4).to_a.shuffle)

(0..4).to_a.zip((0..4).to_a.shuffle).each{|gg|

grpno=gg[0]

unless k1grp[pn][grpno] then

k1grp[pn][grpno]=Array.new

end

d2[gg[1]].each{|psetno|

k1grp[pn][grpno] << "#{tno}_#{psetno}"

}

}

}

}

k1grp.each{|pn,d1|

d1.each{|grp,d2|

p "#{pn} #{grp} #{d2.length}"

}

}

Oj.to_file("training_data/construction/k1grp.oj",k1grp,:mode=>:compat)

**-----------------------------------------------------------------------**

**store_training_data.rb**

**-----------------------------------------------------------------------**

require 'csv'

require 'oj'

require 'fileutils'

user_data="user_data"

preset_data="preset_data"

primer_directory="primer_data"

templ_primer_directory="templ_primer"

pos_homol="pos_homol"

pseudo_sentense="pseudo_sentense"

def tp_to_tpno(tp)

tno=tp.split("_")[0].to_i

psetno=tp.split("_")[1].to_i

tpno=sprintf("%10.2d",tno).strip.concat(sprintf("%10.3d",psetno).strip)

return tpno

end

def delete_pss_files(sno,traintest,posneg)

FileUtils.rm(Dir.glob("training_data/set_#{sno}/#{traintest}/#{posneg}/*.txt"))

end

def save_pseudosentence(setno,traintest,posneg,tp,pss)

tpno=tp_to_tpno(tp)

File.open("training_data/set_#{setno}/#{traintest}/#{posneg}/#{tpno}.txt","w") do |file|

# p "training_data/set_#{setno}/#{traintest}/#{posneg}/#{tpno}.txt"

file.puts(pss)

end

end

def save_pss(setno,test_train,pos_neg,tp_pss)

p "save #{setno}/#{test_train}/#{pos_neg}"

tp_pss.each{|tp,ps|

save_pseudosentence(setno,test_train,pos_neg,tp,ps)

}

end

########################################3

# template_no-primer_set_numer data in groups for learing RNN

# k1grp[pn][grpno] << "#{tno}_#{psetno}"

k1grp=Oj.load_file("training_data/construction/k1grp.oj",:mode=>:compat)

# Hash of pseudo-sentences with the key template_no => primer_set_numer

penta_words=Oj.load_file("pseudo_sentense/penta_words.oj",:mode=>:compat)

# k1grp: positive/negative -> group_number -> pseudo-sentence

# p penta_words

# Make directories for training and test

arr_sets=[1,2,3,4,5]

tt_sets=["test","train"]

pn_sets=["pos","neg"]

arr_sets.each{|sno|

tt_sets.each{|tt|

pn_sets.each{|pn|

p "#{sno} #{tt} #{pn}"

p "training_data/set_#{sno}/#{tt}/#{pn}"

dirname2 = "training_data/set_#{sno}/#{tt}/#{pn}"

unless File.directory?(dirname2)

p "making dir training_data/set_#{sno}/#{tt}/#{pn}"

FileUtils.mkdir_p(dirname2)

end

}

}

}

pseudo_sentence=Hash.new

penta_words.each{|tno,d1|

d1.each{|psetno,d2|

# p "#{tno} #{psetno} #{d2.length}"

pseudo_sentence["#{tno}_#{psetno}"]=d2

}

}

Oj.to_file("training_data/construction/pseudo_sentence.oj",pseudo_sentence,:mode=>:compat)

# Make a Hash: positive/negative=>group_no for group validation=>template-primer_set numbers=>pseudosentences

k2grp=Hash.new

# k2grp[pn][grp][tp]=pseudo_sentence[tp].join(" ")

vocab0=Array.new

# k1grp[pn][grpno] << "#{tno}_#{psetno}"

k1grp.each{|pn,d1|

k2grp[pn]=Hash.new

d1.each{|grp,d2|

k2grp[pn][grp]=Hash.new

# p "#{pn} #{grp}"

d2.each{|tp|

k2grp[pn][grp][tp]=pseudo_sentence[tp].join(" ")

# tp: template number_primer set number

vocab0 << pseudo_sentence[tp]

}

}

}

k2grp.each{|pn,d1|

d1.each{|grp,d2|

p "#{pn} #{grp} #{d2.length}"

}

}

Oj.to_file("training_data/construction/k2grp.oj",k2grp,:mode=>:compat)

# delete files in training folder

traintest=["train","test"]

posneg=["pos","neg"]

arr_sets.each{|sno|

traintest.each{|tt|

posneg.each{|pn|

delete_pss_files(sno,tt,pn)

}

}

}

# reconstruct groups for cross-validation

# Group numbers => 1 to 5

k3grp=Hash.new

k2grp.each{|pn,d1|

d1.each{|grp,d2|

unless k3grp[grp.to_i+1] then

k3grp[grp.to_i+1]=Hash.new

end

k3grp[grp.to_i+1][pn]=d2

}

}

#check k3grp

k3grp.each{|grp,d1|

d1.each{|pn,d2|

d2.each{|tp,ps|

# p "#{grp} #{pn} #{tp} #{ps}"

p "#{grp} #{pn}"

# pn="0" or "1"

}

}

}

# test group in cross-validation

a_test_group=(1..5).to_a

test_train=""

pos_neg=""

set_no=0

a_test_group.each{|ttgrp|

setno=ttgrp

k3grp.each{|grp,d1|

d1.each{|pn,d2|

p pn

if grp==ttgrp then

test_train="test"

else

test_train="train"

end

if pn=="0" then

pos_neg="neg"

else

pos_neg="pos"

end

tp_pss=d2

p "#{setno} #{test_train} #{pos_neg}"

save_pss(setno,test_train,pos_neg,tp_pss)

}

}

}

# Make hash for order for training or test

pss_order0=Hash.new # order of templat-prime_set number and pseudosenteces in each group

a_test_group.each{|ttgrp|

setno=ttgrp

pss_order0[setno]=Hash.new

k3grp.each{|grp,d1|

d1.each{|pn,d2|

if grp==ttgrp then

test_train="test"

else

test_train="train"

end

if pn=="0" then

pos_neg="neg"

else

pos_neg="pos"

end

unless pss_order0[setno][test_train] then

pss_order0[setno][test_train]=Hash.new

end

unless pss_order0[setno][test_train][pos_neg] then

pss_order0[setno][test_train][pos_neg]=Array.new

end

d2.each{|tp,pss|

tpno=tp_to_tpno(tp)

pss_order0[setno][test_train][pos_neg] << [tpno,tp]

}

}

}

}

# Make files entitled template_number and primer_set_numer

p2ss_order=Hash.new

pss_order0.each{|setno,d1|

p2ss_order[setno]=Hash.new

d1.each{|traintest,d2|

p2ss_order[setno][traintest]=Hash.new

d2.each{|posneg,tps|

p "#{posneg} #{tps[0]}"

p2ss_order[setno][traintest][posneg]=tps.sort_by{|x| x[0]}

# p2ss_order[set_no][traintest][posneg]=tps.sort_by{|x| x[0]}

}

}

}

Oj.to_file("training_data/construction/pss_order.oj",p2ss_order,:mode=>:compat)

# Save vocaborary in appropriate position

vocab_u=vocab0.flatten.uniq

a_test_group.each{|ttgrp|

File.open("training_data/set_#{ttgrp}/imdb.vocab","w") do |file|

vocab_u.each{|w|

file.puts(w)

}

end

}

**-----------------------------------------------------------------------**

**RNN learning with split validation data and evaluate predictions**

Summary: Learning RNN by pseudo-sentence, at the same time, the validation history about each pseudo-sentence is stored (**train_pcr.py**). Make evaluation data from stored history-data during validation by RNN (**evaluate_prediction.rb**).

**train_pcr.py**

**-----------------------------------------------------------------------**

import glob

import pathlib

import re

from statistics import mean

import csv

import pprint

import torch

from torch import nn, optim

from torch.utils.data import (Dataset, DataLoader, TensorDataset)

import tqdm

import numpy as np

import os

import json

remove_marks_regex = re.compile("[,\.\(\)\[\]\*:;]|<.*?>")

shift_marks_regex = re.compile("([?!])")

def text2ids(text, vocab_dict):

# remove marks other than !?

text = remove_marks_regex.sub("", text)

# Insert spaces between !?

text = shift_marks_regex.sub(r" \1 ", text)

tokens = text.split()

return [vocab_dict.get(token, 0) for token in tokens]

def list2tensor(token_idxes, max_len=100, padding=True):

if len(token_idxes) > max_len:

token_idxes = token_idxes[:max_len]

n_tokens = len(token_idxes)

if padding:

token_idxes = token_idxes \

+ [0] * (max_len - len(token_idxes))

return torch.tensor(token_idxes, dtype=torch.int64), n_tokens

import torch

from torch import nn, optim

from torch.utils.data import (Dataset, DataLoader, TensorDataset)

import tqdm

class IMDBDataset(Dataset):

def __init__(self, dir_path, train=True, max_len=100, padding=True):

self.max_len = max_len

self.padding = padding

path = pathlib.Path(dir_path)

vocab_path = path.joinpath("imdb.vocab")

print(vocab_path)

# Read vocabulary files

self.vocab_array = vocab_path.open().read().strip().splitlines()

#

self.vocab_dict = dict((w, i+1) for (i, w) in enumerate(self.vocab_array))

if train:

target_path = path.joinpath("train")

else:

target_path = path.joinpath("test")

pos_files = sorted(glob.glob(str(target_path.joinpath("pos/*.txt"))))

print(pos_files)

neg_files = sorted(glob.glob(str(target_path.joinpath("neg/*.txt"))))

# lable: neg→ 0, pos→ 1

#

self.labeled_files = list(zip([0]*len(neg_files), neg_files )) + list(zip([1]*len(pos_files), pos_files))

@property

def vocab_size(self):

return len(self.vocab_array)

def __len__(self):

return len(self.labeled_files)

def __getitem__(self, idx):

label, f = self.labeled_files[idx]

#

# data = open(f).read().lower()

#

data = open(f).read()

#

data = text2ids(data, self.vocab_dict)

#

data, n_tokens = list2tensor(data, self.max_len, self.padding)

return data, label, n_tokens

class SequenceTaggingNet(nn.Module):

def __init__(self, num_embeddings, embedding_dim=50, hidden_size=50, num_layers=1, dropout=0.2):

super().__init__()

self.emb = nn.Embedding(num_embeddings, embedding_dim, padding_idx=0)

self.lstm = nn.LSTM(embedding_dim, hidden_size, num_layers, batch_first=True, dropout=dropout)

self.linear = nn.Linear(hidden_size, 1)

def forward(self, x, h0=None, l=None):

#

# xは(batch_size, step_size)

# -> (batch_size, step_size, embedding_dim)

x = self.emb(x)

#

# -> (batch_size, step_size, hidden_dim)

x, h = self.lstm(x, h0)

#

# -> (batch_size, 1)

if l is not None:

#

x = x[list(range(len(x))), l-1, :]

else:

#

x = x[:, -1, :]

#

x = self.linear(x)

#

# (batch_size, 1) -> (batch_size, )

x = x.squeeze()

return x

def eval_net(net, data_loader, device="cpu"):

net.eval()

ys = []

ypreds = []

for x, y, l in data_loader:

# print('x %s' % x)

# print("y %s" % y)

# print("l %s" % l)

x = x.to(device)

y = y.to(device)

l = l.to(device)

with torch.no_grad():

y_pred = net(x, l=l)

y_pred = (y_pred > 0).long()

# print('y_pred %s' % y_pred)

if len(list(y_pred.size()))>0:

ys.append(y)

ypreds.append(y_pred)

# ys.append(y)

# ypreds.append(y_pred)

ys = torch.cat(ys)

# print('ys %s' % ys)

ypreds = torch.cat(ypreds)

# print('ypreds %s' % ypreds)

acc = (ys == ypreds).float().sum() / len(ys)

# print("acc %s" % acc)

return acc.item()

def eval_net_test(net, data_loader, device="cpu"):

net.eval()

ys = []

ypreds = []

for x, y, l in data_loader:

x = x.to(device)

y = y.to(device)

l = l.to(device)

# print(x)

# print(y)

# print(l)

with torch.no_grad():

y_pred = net(x, l=l)

# print(y_pred)

y_pred = (y_pred > 0).long()

# print(y_pred)

if len(list(y_pred.size()))>0:

ys.append(y)

ypreds.append(y_pred)

#ys.append(y)

#ypreds.append(y_pred)

ys = torch.cat(ys)

ypreds = torch.cat(ypreds)

# print(ys)

# print(ypreds)

acc = (ys == ypreds).float().sum() / len(ys)

return acc.item(),ys,ypreds

def my_makedirs(path):

if not os.path.isdir(path):

os.makedirs(path)

################################################################################

#torch.save(net, 'learned_net_%s.pth' % setno) # Store learned network

tmser=[56]

# tmser=[60]

for tmtmp in tmser:

print("tmtemp = %s" % tmtmp)

epochs={}

for s0 in range(5):

setno=s0+1

rec_path="learned_sets/set_%s" % setno

my_makedirs(rec_path)

epochs[setno]={}

print("template_no=%s" % setno)

train_data = IMDBDataset("training_data/set_%s/" % setno)

test_data = IMDBDataset("training_data/set_%s/" % setno, train=False)

train_loader = DataLoader(train_data, batch_size=32, shuffle=True, num_workers=4)

test_loader = DataLoader(test_data, batch_size=32, shuffle=False, num_workers=4)

#

net = SequenceTaggingNet(train_data.vocab_size+1, num_layers=2)

net.to("cuda:0")

opt = optim.Adam(net.parameters())

loss_f = nn.BCEWithLogitsLoss()

# print(setno)

ys_ypreds={}

for epoch in range(200):

losses = []

net.train()

for x, y, l in tqdm.tqdm(train_loader):

x = x.to("cuda:0")

y = y.to("cuda:0")

l = l.to("cuda:0")

y_pred = net(x, l=l)

# print(y_pred)

loss = loss_f(y_pred, y.float())

net.zero_grad()

loss.backward()

opt.step()

losses.append(loss.item())

train_acc = eval_net(net, train_loader, "cuda:0")

val_acc,ys,y_pred = eval_net_test(net, test_loader, "cuda:0")

ys_list=ys.tolist()

ypred_list=y_pred.tolist()

ys_ypreds[epoch]=[ys_list,ypred_list]

print(epoch, mean(losses), train_acc, val_acc)

epochs[setno][epoch]=[mean(losses), train_acc, val_acc]

torch.save(SequenceTaggingNet, 'learned_sets/set_%s/learned_net.pth' % setno) #

torch.save(opt.state_dict(), 'learned_sets/set_%s/optimizer.pth' % setno)

torch.save(net, "learned_sets/set_%s/net.pkl" % setno)

file_path="%s/ys_ypreds.json" % rec_path

with open(file_path, 'w') as outfile:

json.dump(ys_ypreds, outfile)

print("End of cycle")

# print(epochs)

save_dir="training_data/set_%s/" % setno

with open('training_data/set_%s/epoch_results.csv' % setno, 'w') as f:

writer = csv.writer(f)

for setno,data1 in epochs.items():

for epoch,d2 in data1.items():

writer.writerow([setno,epoch,d2[0],d2[1],d2[2]])

**-----------------------------------------------------------------------**

**evaluate_prediction.rb**

**-----------------------------------------------------------------------**

require 'json'

require 'oj'

require 'csv'

pseudo_sentense="pseudo_sentense"

# p2ss_order[setno][traintest][posneg]=tps.sort_by{|x| x[0]}

pss_order=Oj.load_file("training_data/construction/pss_order.oj",:mode=>:compat)

pss_l0=Hash.new

pss_order.each{|setno,v1|

pss_l0[setno]=Hash.new

v1["test"].each{|v2|

if v2[0]=="neg" then

pss_l0[setno]["neg"]=Array.new

v2[1].each{|v3|

pss_l0[setno]["neg"] << v3[0]

}

elsif v2[0]=="pos" then

pss_l0[setno]["pos"]=Array.new

v2[1].each{|v3|

pss_l0[setno]["pos"] << v3[0]

}

end

# v2[1]["neg"].each{|v3|

# p v3

# pss_learning_set[setno] << v3[0]

# }

# v2[1]["pos"].each{|v3|

# pss_learning_set[setno] << v3[0]

# }

}

}

# p pss_l0

p1ss_learn=Hash.new

pss_l0.each{|setno,d1|

p1ss_learn[setno.to_i]=[d1["neg"],d1["pos"]].flatten

}

# p p1ss_learn

ys_ypreds=Hash.new

(1..5).to_a.each{|setno|

File.open("learned_sets/set_#{setno}/ys_ypreds.json") do |j|

ys_ypreds[setno] = JSON.load(j)

end

}

ys_ypreds.each{|k1,v1|

# p "#{k1} #{v1.length}"

}

evaluate_sets=Hash.new

p1ss_learn.each{|setno,d1|

# p setno

# p ys_ypreds[setno]

tgt_yyset=ys_ypreds[setno]["9"]

rset= tgt_yyset[0].zip(tgt_yyset[1])

evaluate_sets[setno]= p1ss_learn[setno].zip(rset)

}

list_eval=Hash.new

evaluate_sets.each{|setno,res|

res.each{|v|

tpset=v[0]

list_eval[tpset]=v[1]

}

}

CSV.open("learned_sets/train_results.csv","w") do |csv|

list_eval.sort_by{|x| x[0]}.each{|v|

tno=v[0][0,2].to_i

psetno=v[0][3,3].to_i

csv << ["#{tno}_#{psetno}",v].flatten

}

end

gibbs_plot=Oj.load_file("#{pseudo_sentense}/gibbs_plot.oj",:mode=>:compat)

gibbs_data=Hash.new

gibbs_plot[1.to_s].each{|k1,v1|

gibbs_data[k1]=v1

}

tp_pred=Hash.new

list_eval.sort_by{|x| x[0]}.each{|v|

tno=v[0][0,2].to_i

psetno=v[0][3,3].to_i

# p v[1]

tp_pred["#{tno}_#{psetno}"]=v[1]

# p gibbs_data["#{tno}_#{psetno}"]

}

g_plot=Hash.new

a_x=Array.new

a_y=Array.new

tp_pred.each{|tp,pred|

if gibbs_data[tp] then

gibbsdata= gibbs_data[tp]

gibbs_x=gibbsdata[1][1][0][2].to_f

a_x << gibbs_x

gibbs_y=gibbsdata[1][1][1][2].to_f

a_y << gibbs_y

p "#{gibbs_x} #{gibbs_y}"

unless g_plot[pred] then

g_plot[pred]=Hash.new

end

g_plot[pred][tp]=[gibbs_x,gibbs_y]

end

}

g_plot.each{|pred,d1|

d1.each{|tp,d2|

p "#{tp} #{d2}"

}

}

p a_x.min

p a_y.min

# p g_plot[[0,0]]

File.open("learned_sets/pr_gibbs_plot.txt","w") do |file|

file.puts("# Negative result Negative pred")

file.puts("# x\ty")

g_plot[[0,0]].each{|tp,eset|

file.puts("#{eset[0]}\t#{eset[1]}")

}

file.puts("")

file.puts("")

file.puts("")

file.puts("# Negative result Positive pred")

file.puts("# x\ty")

g_plot[[0,1]].each{|tp,eset|

file.puts("#{eset[0]}\t#{eset[1]}")

}

file.puts("")

file.puts("")

file.puts("")

file.puts("# Positive result Positive pred")

file.puts("# x\ty")

g_plot[[1,1]].each{|tp,eset|

file.puts("#{eset[0]}\t#{eset[1]}")

}

file.puts("")

file.puts("")

file.puts("")

file.puts("# Positive result Negative pred")

file.puts("# x\ty")

g_plot[[1,0]].each{|tp,eset|

file.puts("#{eset[0]}\t#{eset[1]}")

}

end

**-----------------------------------------------------------------------**

**Test the pseudo-sentences of test-data by learned RNN**

Summary: The pseudo-sentences for test was made by the same process as training and set them in a data set folder (**construct_test_data.rb**) and then test the data with RNN which had learned with learning data (**test_template_primerset.py**).

**construct_test_data.rb**

**-----------------------------------------------------------------------**

require 'csv'

require 'oj'

user_data="user_data"

preset_data="preset_data"

primer_directory="primer_data"

templ_primer_directory="templ_primer"

pos_homol="pos_homol"

pseudo_sentense="pseudo_sentense"

########################################3

i=0

a_templ=Array.new

pcr_r=Hash.new

CSV.foreach("#{user_data}/pcr_results.csv") do |row|

if i==0 then

a_templ=row[1..-1].map{|x| x.to_i}

else

psetno=row[0].to_i

pcr_r[psetno]=Hash.new

row[1..-1].map{|x| x.to_i}.each_with_index{|r,idx|

pcr_r[psetno][a_templ[idx]]=r

}

end

i+=1

end

# Make a Hash

pcr_ts=Hash.new

pcr_r.each{|psetno,d1|

d1.each{|tno,r|

unless pcr_ts[tno] then

pcr_ts[tno]=Hash.new

end

pcr_ts[tno][psetno]=r

}

}

# Set group

k_grp_0=Hash.new

grp=[0] # Set groups to 1

pcr_ts.each{|tno,d1|

k_grp_0[tno]=Hash.new

[0,1].each{|pn|

k_grp_0[tno][pn]=Hash.new

tgt_grp=d1.select{|k,v| v==pn}

p "#{tno} #{tgt_grp}"

if tgt_grp then

d1.select{|k,v| v==pn}.to_a.each_with_index{|e,idx| # e -> primer-set number

grpno=0

unless k_grp_0[tno][pn][grpno] then

k_grp_0[tno][pn][grpno]=Array.new

end

psetno=e[0]

k_grp_0[tno][pn][grpno] << psetno # groups were set on positive/negative -> group number

}

end

}

}

k_grp_0.each{|tno,d1|

d1.each{|pn,d2|

p pn

p d2

}

}

k1grp=Hash.new

k_grp_0.each{|tno,d1|

d1.each{|pn,d2|

p "#{tno} #{d2}"

unless k1grp[pn] then

k1grp[pn]=Hash.new

end

grpno=0

unless k1grp[pn][grpno] then

k1grp[pn][grpno]=Array.new

end

if d2[0] then

d2[0].each{|psetno|

k1grp[pn][grpno] << "#{tno}_#{psetno}"

}

end

}

}

k1grp.each{|pn,d1|

d1.each{|grpno,tp|

p "#{pn} #{grpno}"

p tp

}

}

# p k_grp_0

k1grp.each{|pn,d1|

d1.each{|grp,d2|

p "#{pn} #{grp} #{d2.length}"

}

}

Dir.mkdir("training_data/construction") unless Dir.exist?("training_data/construction")

Oj.to_file("training_data/construction/k1grp.oj",k1grp,:mode=>:compat)

**-----------------------------------------------------------------------**

**test_template_primerset.py**

**-----------------------------------------------------------------------**

import glob

import pathlib

import re

from statistics import mean

import csv

import pprint

import torch

from torch import nn, optim

from torch.utils.data import (Dataset, DataLoader, TensorDataset)

import tqdm

import numpy as np

import json

remove_marks_regex = re.compile("[,\.\(\)\[\]\*:;]|<.*?>")

shift_marks_regex = re.compile("([?!])")

def text2ids(text, vocab_dict):

# delete letters other than !?

text = remove_marks_regex.sub("", text)

text = shift_marks_regex.sub(r" \1 ", text)

tokens = text.split()

return [vocab_dict.get(token, 0) for token in tokens]

def list2tensor(token_idxes, max_len=100, padding=True):

if len(token_idxes) > max_len:

token_idxes = token_idxes[:max_len]

n_tokens = len(token_idxes)

if padding:

token_idxes = token_idxes \

+ [0] * (max_len - len(token_idxes))

return torch.tensor(token_idxes, dtype=torch.int64), n_tokens

import torch

from torch import nn, optim

from torch.utils.data import (Dataset, DataLoader, TensorDataset)

import tqdm

class IMDBDataset(Dataset):

def __init__(self, dir_path, train=True, max_len=100, padding=True):

self.max_len = max_len

self.padding = padding

path = pathlib.Path(dir_path)

vocab_path = path.joinpath("imdb.vocab")

print(vocab_path)

# make vocabulary file

self.vocab_array = vocab_path.open().read().strip().splitlines()

#

self.vocab_dict = dict((w, i+1) for (i, w) in enumerate(self.vocab_array))

if train:

target_path = path.joinpath("train")

else:

target_path = path.joinpath("test")

pos_files = sorted(glob.glob(str(target_path.joinpath("pos/*.txt"))))

print(pos_files)

neg_files = sorted(glob.glob(str(target_path.joinpath("neg/*.txt"))))

# posは1, negは0のlabelを付けて

# (file_path, label) as tuple

self.labeled_files = list(zip([0]*len(neg_files), neg_files )) + list(zip([1]*len(pos_files), pos_files))

@property

def vocab_size(self):

return len(self.vocab_array)

def __len__(self):

return len(self.labeled_files)

def __getitem__(self, idx):

label, f = self.labeled_files[idx]

#

data = open(f).read()

#

data = text2ids(data, self.vocab_dict)

#

data, n_tokens = list2tensor(data, self.max_len, self.padding)

return data, label, n_tokens

class SequenceTaggingNet(nn.Module):

def __init__(self, num_embeddings, embedding_dim=50, hidden_size=50, num_layers=1, dropout=0.2):

super().__init__()

self.emb = nn.Embedding(num_embeddings, embedding_dim, padding_idx=0)

self.lstm = nn.LSTM(embedding_dim, hidden_size, num_layers, batch_first=True, dropout=dropout)

self.linear = nn.Linear(hidden_size, 1)

def forward(self, x, h0=None, l=None):

#

# xは(batch_size, step_size)

# -> (batch_size, step_size, embedding_dim)

x = self.emb(x)

#

# xは(batch_size, step_size, embedding_dim)

# -> (batch_size, step_size, hidden_dim)

x, h = self.lstm(x, h0)

#

# xは(batch_size, step_size, hidden_dim)

# -> (batch_size, 1)

if l is not None:

#

x = x[list(range(len(x))), l-1, :]

else:

#

x = x[:, -1, :]

#

x = self.linear(x)

#

# (batch_size, 1) -> (batch_size, )

x = x.squeeze()

return x

def eval_net(net, data_loader, device="cpu"):

net.eval()

ys = []

ypreds = []

for x, y, l in data_loader:

# print('x %s' % x)

# print("y %s" % y)

# print("l %s" % l)

x = x.to(device)

y = y.to(device)

l = l.to(device)

with torch.no_grad():

y_pred = net(x, l=l)

y_pred = (y_pred > 0).long()

# print('y_pred %s' % y_pred)

ys.append(y)

ypreds.append(y_pred)

ys = torch.cat(ys)

# print('ys %s' % ys)

ypreds = torch.cat(ypreds)

# print('ypreds %s' % ypreds)

acc = (ys == ypreds).float().sum() / len(ys)

# print("acc %s" % acc)

return acc.item()

def eval_net_test(net, data_loader, device="cpu"):

net.eval()

ys = []

ypreds = []

for x, y, l in data_loader:

x = x.to(device)

y = y.to(device)

l = l.to(device)

# print(x)

# print(y)

# print(l)

with torch.no_grad():

y_pred = net(x, l=l)

# print(y_pred)

y_pred = (y_pred > 0).long()

# print(y_pred)

ys.append(y)

ypreds.append(y_pred)

ys = torch.cat(ys)

ypreds = torch.cat(ypreds)

# print(ys)

# print(ypreds)

acc = (ys == ypreds).float().sum() / len(ys)

return acc.item(),ys,ypreds

#torch.save(net, 'learned_net_%s.pth' % setno) #

tmser=[56]

# tmser=[60]

for tmtmp in tmser:

print("tmtemp = %s" % tmtmp)

epochs={}

for s0 in range(5):

setno=s0+1

net=torch.load("learned_sets/set_%s/net.pkl" % setno)

# train_data = IMDBDataset("training_data/set_%s/" % setno)

test_data = IMDBDataset("training_data/set_1/", train=False)

# train_loader = DataLoader(train_data, batch_size=32, shuffle=True, num_workers=4)

test_loader = DataLoader(test_data, batch_size=32, shuffle=False, num_workers=4)

#

test_acc,ys,ypreds = eval_net_test(net, test_loader, "cuda:0")

print(test_acc)

# print(ys)

# print(ypreds)

with open('test_results/set_%s/test_acc.json' % setno,'w') as f:

json.dump(test_acc, f)

with open('test_results/set_%s/test_files.json' % setno,'w') as f:

ys_list=ys.tolist()

# print(ys_list)

json.dump(ys_list, f)

with open('test_results/set_%s/predict_results.json' % setno,'w') as f:

ypreds_list=ypreds.tolist()

# print(ypreds_list)

json.dump(ypreds_list, f)

**-----------------------------------------------------------------------**

**Make cross tables, color-excel files and graphs**

Summary: Prepare processed data from train_PCR.py (**prediction_line_graph.rb**). Then analyse for cross table (**cross_tab_199.rb**), colored excel, (**cross_tab_199.rb**) line graph (**line_graph_accuracy.py**) and scatter graph (**gibbs_plot_prediction.rb**).

**prediction_line_graph.rb**

**-----------------------------------------------------------------------**

require 'json'

require 'oj'

require 'csv'

require 'fileutils'

class Array

def sum

reduce(:+)

end

def mean

sum.to_f / size

end

def var

m = mean

reduce(0) { |a,b| a + (b - m) ** 2 } / (size - 1)

end

def sd

Math.sqrt(var)

end

end

############################################

pseudo_sentense="pseudo_sentense"

# p2ss_order[setno][traintest][posneg]=tps.sort_by{|x| x[0]}

pss_order=Oj.load_file("training_data/construction/pss_order.oj",:mode=>:compat)

pss_l0=Hash.new

pss_order.each{|setno,v1|

pss_l0[setno]=Hash.new

v1["test"].each{|v2|

if v2[0]=="neg" then

pss_l0[setno]["neg"]=Array.new

v2[1].each{|v3|

pss_l0[setno]["neg"] << v3[0]

}

elsif v2[0]=="pos" then

pss_l0[setno]["pos"]=Array.new

v2[1].each{|v3|

pss_l0[setno]["pos"] << v3[0]

}

end

# v2[1]["neg"].each{|v3|

# p v3

# pss_learning_set[setno] << v3[0]

# }

# v2[1]["pos"].each{|v3|

# pss_learning_set[setno] << v3[0]

# }

}

}

# p pss_l0

p1ss_learn=Hash.new

pss_l0.each{|setno,d1|

p1ss_learn[setno.to_i]=[d1["neg"],d1["pos"]].flatten

}

# p p1ss_learn

ys_ypreds=Hash.new

(1..5).to_a.each{|setno|

File.open("learned_sets/set_#{setno}/ys_ypreds.json") do |j|

ys_ypreds[setno] = JSON.load(j)

end

}

# ys_ypreds[epoch]=[ys_list,ypred_list]

ser_pred=Hash.new

ys_ypreds.each{|k1,v1|

p "#{k1} "

ser_pred[k1.to_i]=Hash.new

v1.each{|epoch,ylist|

# p epoch

prset= ylist[0].zip(ylist[1])

n_hit=prset.select{|x| x.uniq.length==1}.length

n_all=prset.length

a_pos=prset.select{|x| x[0]==1}

a_pos_hit=a_pos.select{|x| x[1]==1}

a_neg=prset.select{|x| x[0]==0}

a_neg_hit=a_neg.select{|x| x[1]==0}

accuracy=n_hit/n_all.to_f

acc_pos=a_pos_hit.length/a_pos.length.to_f

acc_neg=a_neg_hit.length/a_neg.length.to_f

p "#{epoch} #{accuracy} #{acc_pos} #{acc_neg}"

ser_pred[k1.to_i][epoch.to_i]=[accuracy,acc_pos,acc_neg]

}

}

epoch_accuracy=Hash.new

ser_pred.each{|serno,d1|

d1.each{|epoch,d2|

unless epoch_accuracy[epoch] then

epoch_accuracy[epoch]=Hash.new

end

unless epoch_accuracy[epoch]["all"] then

epoch_accuracy[epoch]["all"]=Array.new

end

unless epoch_accuracy[epoch]["pos"] then

epoch_accuracy[epoch]["pos"]=Array.new

end

unless epoch_accuracy[epoch]["neg"] then

epoch_accuracy[epoch]["neg"]=Array.new

end

epoch_accuracy[epoch]["all"] << d2[0]

epoch_accuracy[epoch]["pos"] << d2[1]

epoch_accuracy[epoch]["neg"] << d2[2]

}

}

e1epoch_macc=Hash.new

epoch_accuracy.each{|epoch,d1|

e1epoch_macc[epoch]=Hash.new

d1.each{|pn,d2|

e1epoch_macc[epoch][pn]=[d2.mean,d2.sd]

}

}

ep_graph=[0,1,3,10,33,100,133,199]

ep_graph.each{|e|

p "#{e} #{e1epoch_macc[e]}"

}

FileUtils.mkdir_p("analysis_prediction")

# e1epoch_macc[epoch][pn]=[d2.mean,d2.sd]

File.open("analysis_prediction/epoch_accuracy1.json", 'w') do |file|

str = JSON.dump(e1epoch_macc, file)

end

# evaluate_sets=Hash.new

# p1ss_learn.each{|setno,d1|

# # p setno

# # p ys_ypreds[setno]

# tgt_yyset=ys_ypreds[setno]["9"]

# rset= tgt_yyset[0].zip(tgt_yyset[1])

# evaluate_sets[setno]= p1ss_learn[setno].zip(rset)

# }

# list_eval=Hash.new

# evaluate_sets.each{|setno,res|

# res.each{|v|

# tpset=v[0]

# list_eval[tpset]=v[1]

# }

# }

**-----------------------------------------------------------------------**

**cross_tab_199.rb**

**-----------------------------------------------------------------------**

require 'json'

require 'oj'

require 'csv'

require 'fileutils'

require 'axlsx'

class Array

def sum

reduce(:+)

end

def mean

sum.to_f / size

end

def var

m = mean

reduce(0) { |a,b| a + (b - m) ** 2 } / (size - 1)

end

def sd

Math.sqrt(var)

end

end

############################################

pseudo_sentense="pseudo_sentense"

# p2ss_order[setno][traintest][posneg]=tps.sort_by{|x| x[0]}

pss_order=Oj.load_file("training_data/construction/pss_order.oj",:mode=>:compat)

pss_l0=Hash.new

pss_order.each{|setno,v1|

pss_l0[setno]=Hash.new

v1["test"].each{|v2|

if v2[0]=="neg" then

pss_l0[setno]["neg"]=Array.new

v2[1].each{|v3|

pss_l0[setno]["neg"] << v3[0]

}

elsif v2[0]=="pos" then

pss_l0[setno]["pos"]=Array.new

v2[1].each{|v3|

pss_l0[setno]["pos"] << v3[0]

}

end

# v2[1]["neg"].each{|v3|

# p v3

# pss_learning_set[setno] << v3[0]

# }

# v2[1]["pos"].each{|v3|

# pss_learning_set[setno] << v3[0]

# }

}

}

p1ss_learn=Hash.new

pss_l0.each{|setno,d1|

p1ss_learn[setno.to_i]=[d1["neg"],d1["pos"]].flatten

}

# p p1ss_learn

ys_ypreds=Hash.new

(1..5).to_a.each{|setno|

File.open("learned_sets/set_#{setno}/ys_ypreds.json") do |j|

ys_ypreds[setno] = JSON.load(j)

end

}

# ys_ypreds[group][epoch]=[ys_list,ypred_list]

pred_all=Array.new

pred_grp=Hash.new

ys_ypreds.each{|group,d1|

rspr=d1["199"]

ziprs=rspr[0].zip(rspr[1])

pred_grp[group]=ziprs

ziprs.each{|rp|

pred_all << rp

}

}

p pred_grp

rcase=Array.new

[0,1].each{|e1|

[0,1].each{|e2|

rcase << [e1,e2]

}

}

# Cross table for each set

crosstbl=Hash.new

pred_grp.each{|grp,zpred|

crosstbl[grp]=Hash.new

rcase.each{|e|

crosstbl[grp][e]=zpred.select{|x| x==e}.length

}

}

ssa=Hash.new

CSV.open("analysis_prediction/group_cross_tbls.csv","w") do |csv|

crosstbl.each{|grp,d1|

tp=d1[[1,1]]

fp=d1[[0,1]]

tn=d1[[0,0]]

fn=d1[[1,0]]

csv << [grp]

csv << ["prediction result", "negative", "positive"]

csv << ["negative", tn, fn]

csv << ["positive", fp, tp]

csv << []

sensitivity=tp/(tp+fn).to_f

specificity=tn/(fp+tn).to_f

accuracy=(tp+tn)/(tp+fp+tn+fn).to_f

# csv << ["Sensitivity=",sensitivity,"","Specificity=",specificity,"","Accuracy=",accuracy]

unless ssa["sensitvity"] then

ssa["sensitvity"]=Array.new

end

unless ssa["specificity"] then

ssa["specificity"]=Array.new

end

unless ssa["accuracy"] then

ssa["accuracy"]=Array.new

end

ssa["sensitvity"]<<sensitivity

ssa["specificity"]<<specificity

ssa["accuracy"]<<accuracy

}

csv << ["Sensitivity",ssa["sensitvity"]].flatten

csv << ["Specificity",ssa["specificity"]].flatten

csv << ["Accuracy",ssa["accuracy"]].flatten

end

clrs=Hash.new

rcase.each{|e|

# p e

clrs[e]=pred_all.select{|x| x==e}.length

}

CSV.open("analysis_prediction/cross_tbl.csv","w") do |csv|

csv << ["result prediction", "negative", "positive"]

csv << ["negative", clrs[[0,0]], clrs[[0,1]]]

csv << ["positive", clrs[[1,0]], clrs[[1,1]]]

end

s0set_res=Hash.new

a_np=[["neg",0],["pos",1]]

pred_grp.each{|serno,d1|

s0set_res[serno]=Hash.new

a_np.each{|np|

psno=pss_l0[serno.to_s][np[0]]

rspair=d1.select{|x| x[0]==np[1]}

ps_res=psno.zip(rspair)

s0set_res[serno][np]=ps_res

}

}

tpset_rp=Hash.new

s0set_res.each{|serno,d1|

d1.each{|np,d2|

# p "#{serno} #{np}"

d2.each{|ps|

# p ps

templ=ps[0][0,2].to_i

pset=ps[0][2,3].to_i

unless tpset_rp[pset] then

tpset_rp[pset]=Hash.new

end

rp=ps[1]

result_pred="#{rp[0]}-#{rp[1]}"

tpset_rp[pset][templ]=result_pred

}

}

}

# alteration to excel record

t2tpset=Hash.new

tpset_rp.sort_by{|k,v| k}.each{|x|

pset=x[0].to_i

d1=x[1]

t2tpset[pset]=Array.new

d1.sort_by{|k,v| k}.each{|x2|

templ=x2[0].to_i

ps=x2[1]

# p "#{pset} #{templ} #{ps}"

t2tpset[pset][templ]=ps

}

}

p t2tpset

# Make Color excel

p = Axlsx::Package.new

wb = p.workbook

sheet = wb.add_worksheet(:name => "Prediction_result")

styles=p.workbook.styles

cstyle=Hash.new

cstyle["1-1"] = sheet.styles.add_style(:bg_color => "FF0000", :fg_color => "FFFFFF")

cstyle["1-0"] = sheet.styles.add_style(:bg_color => "FFC0CB", :fg_color => "000000")

cstyle["0-0"] = sheet.styles.add_style(:bg_color => "00008B", :fg_color => "FFFFFF")

cstyle["0-1"] = sheet.styles.add_style(:bg_color => "87CEFA", :fg_color => "000000")

cstyle["n"] = sheet.styles.add_style(:bg_color => "FFFFFF", :fg_color => "FFFFFF")

psets=(1..72).to_a

templs=(1..31).to_a

sheet.add_row ["pset",templs].flatten

psets.each{|pset|

row_arr=[]

row_arr << pset

templs.each{|tmpl|

if t2tpset[pset][tmpl] then

row_arr << t2tpset[pset][tmpl]

else

row_arr << "n"

end

}

sheet.add_row row_arr

templs.each{|tmpl|

p "#{pset} #{tmpl}"

rp=""

if t2tpset[pset][tmpl] then

rp=t2tpset[pset][tmpl]

else

rp="n"

end

# Set values each cells and then apply any style

sheet.rows[pset].cells[tmpl].value=rp

sheet.rows[pset].cells[tmpl].style=cstyle[rp]

}

}

p.serialize("analysis_prediction/prediction_cross_table.xlsx")

**-----------------------------------------------------------------------**

**line_graph_accuracy.py**

**-----------------------------------------------------------------------**

import json

import numpy as np

import matplotlib.pyplot as plt

json_open = open('analysis_prediction/epoch_accuracy1.json', 'r')

epoch_accuracy = json.load(json_open)

# e1epoch_macc[epoch][pn]=[d2.mean,d2.sd]

gplot={}

axises=["x","y","yerr"]

for k,v in epoch_accuracy.items():

for k2,v2 in v.items():

if k2 not in gplot.keys():

gplot[k2]={}

for x in axises:

if x not in gplot[k2].keys():

gplot[k2][x]=[]

gplot[k2]["x"].append(int(k))

gplot[k2]["y"].append(float(v2[0]))

gplot[k2]["yerr"].append(float(v2[1]))

plt.xlabel("Number of epochs")

plt.ylabel("Accuracy")

plt.errorbar(gplot["all"]["x"],gplot["all"]["y"],yerr=gplot["all"]["yerr"])

plt.errorbar(gplot["pos"]["x"],gplot["pos"]["y"],yerr=gplot["pos"]["yerr"])

plt.errorbar(gplot["neg"]["x"],gplot["neg"]["y"],yerr=gplot["neg"]["yerr"])

plt.show()

**-----------------------------------------------------------------------**

**gibbs_plot_prediction.rb**

**-----------------------------------------------------------------------**

require 'json'

require 'oj'

require 'csv'

pseudo_sentense="pseudo_sentense"

# p2ss_order[setno][traintest][posneg]=tps.sort_by{|x| x[0]}

pss_order=Oj.load_file("training_data/construction/pss_order.oj",:mode=>:compat)

pss_l0=Hash.new

pss_order.each{|setno,v1|

pss_l0[setno]=Hash.new

v1["test"].each{|v2|

if v2[0]=="neg" then

pss_l0[setno]["neg"]=Array.new

v2[1].each{|v3|

pss_l0[setno]["neg"] << v3[0]

}

elsif v2[0]=="pos" then

pss_l0[setno]["pos"]=Array.new

v2[1].each{|v3|

pss_l0[setno]["pos"] << v3[0]

}

end

# v2[1]["neg"].each{|v3|

# p v3

# pss_learning_set[setno] << v3[0]

# }

# v2[1]["pos"].each{|v3|

# pss_learning_set[setno] << v3[0]

# }

}

}

# p pss_l0

p1ss_learn=Hash.new

pss_l0.each{|setno,d1|

p1ss_learn[setno.to_i]=[d1["neg"],d1["pos"]].flatten

}

# p p1ss_learn

ys_ypreds=Hash.new

(1..5).to_a.each{|setno|

File.open("learned_sets/set_#{setno}/ys_ypreds.json") do |j|

ys_ypreds[setno] = JSON.load(j)

end

}

ys_ypreds.each{|k1,v1|

# p "#{k1} #{v1.length}"

}

# ys_ypreds[group][epoch]=[ys_list,ypred_list]

pred_all=Array.new

pred_grp=Hash.new

ys_ypreds.each{|group,d1|

rspr=d1["199"]

ziprs=rspr[0].zip(rspr[1])

pred_grp[group]=ziprs

ziprs.each{|rp|

pred_all << rp

}

}

evaluate_sets=Hash.new

p1ss_learn.each{|setno,d1|

# p setno

# p ys_ypreds[setno]

tgt_yyset=ys_ypreds[setno]["9"]

rset= tgt_yyset[0].zip(tgt_yyset[1])

evaluate_sets[setno]= p1ss_learn[setno].zip(rset)

}

list_eval=Hash.new

evaluate_sets.each{|setno,res|

res.each{|v|

tpset=v[0]

list_eval[tpset]=v[1]

}

}

p list_eval

CSV.open("analysis_prediction/train_results.csv","w") do |csv|

list_eval.sort_by{|x| x[0]}.each{|v|

tno=v[0][0,2].to_i

psetno=v[0][3,3].to_i

csv << ["#{tno}_#{psetno}",v].flatten

}

end

gibbs_plot=Oj.load_file("#{pseudo_sentense}/gibbs_plot.oj",:mode=>:compat)

gibbs_data=Hash.new

gibbs_plot[1.to_s].each{|k1,v1|

gibbs_data[k1]=v1

}

tp_pred=Hash.new

list_eval.sort_by{|x| x[0]}.each{|v|

tno=v[0][0,2].to_i

psetno=v[0][3,3].to_i

# p v[1]

tp_pred["#{tno}_#{psetno}"]=v[1]

# p gibbs_data["#{tno}_#{psetno}"]

}

g_plot=Hash.new

a_x=Array.new

a_y=Array.new

tp_pred.each{|tp,pred|

if gibbs_data[tp] then

gibbsdata= gibbs_data[tp]

gibbs_x=gibbsdata[1][1][0][2].to_f

a_x << gibbs_x

gibbs_y=gibbsdata[1][1][1][2].to_f

a_y << gibbs_y

p "#{gibbs_x} #{gibbs_y}"

unless g_plot[pred] then

g_plot[pred]=Hash.new

end

g_plot[pred][tp]=[gibbs_x,gibbs_y]

end

}

g_plot.each{|pred,d1|

d1.each{|tp,d2|

p "#{tp} #{d2}"

}

}

p a_x.min

p a_y.min

# p g_plot[[0,0]]

File.open("analysis_prediction/pr_gibbs_plot.txt","w") do |file|

file.puts("# Negative result Negative pred")

file.puts("# x\ty")

g_plot[[0,0]].each{|tp,eset|

file.puts("#{eset[0]}\t#{eset[1]}")

}

file.puts("")

file.puts("")

file.puts("")

file.puts("# Negative result Positive pred")

file.puts("# x\ty")

g_plot[[0,1]].each{|tp,eset|

file.puts("#{eset[0]}\t#{eset[1]}")

}

file.puts("")

file.puts("")

file.puts("")

file.puts("# Positive result Positive pred")

file.puts("# x\ty")

g_plot[[1,1]].each{|tp,eset|

file.puts("#{eset[0]}\t#{eset[1]}")

}

file.puts("")

file.puts("")

file.puts("")

file.puts("# Positive result Negative pred")

file.puts("# x\ty")

g_plot[[1,0]].each{|tp,eset|

file.puts("#{eset[0]}\t#{eset[1]}")

}

end

**-----------------------------------------------------------------------**
